# Two Chromogranin A-Derived Peptides Induce Calcium Entry in Human Neutrophils by Calmodulin-Regulated Calcium Independent Phospholipase A_2_

**DOI:** 10.1371/journal.pone.0004501

**Published:** 2009-02-19

**Authors:** Dan Zhang, Peiman Shooshtarizadeh, Benoît-Joseph Laventie, Didier André Colin, Jean-François Chich, Jasmina Vidic, Jean de Barry, Sylvette Chasserot-Golaz, François Delalande, Alain Van Dorsselaer, Francis Schneider, Karen Helle, Dominique Aunis, Gilles Prévost, Marie-Hélène Metz-Boutigue

**Affiliations:** 1 INSERM U575, Physiopathologie du Système Nerveux, Strasbourg, France; 2 UPRES-EA 3432, Institut de Bactériologie de la Faculté de Médecine, Université Louis Pasteur, Hôpitaux Universitaires de Strasbourg, Strasbourg, France; 3 INRA, Virologie et Immunologie Moléculaires, Jouy-en-Josas, France; 4 Institut des Neurosciences Cellulaires et Intégratives, UMR 7168 CNRS-Université Louis Pasteur, Strasbourg, France; 5 Département de Réanimation Médicale, Hôpital de Hautepierre, Strasbourg, France; 6 Laboratoire de spectrométrie de masse BioOrganique, IPHC-DSA, ULP, CNRS, UMR7178, Strasbourg, France; 7 Department of Biomedicine, University of Bergen, Bergen, Norway; 8 First Hospital, Chongqing University of Medical Sciences, Chongqing, China; Emory University, United States of America

## Abstract

**Background:**

Antimicrobial peptides derived from the natural processing of chromogranin A (CgA) are co-secreted with catecholamines upon stimulation of chromaffin cells. Since PMNs play a central role in innate immunity, we examine responses by PMNs following stimulation by two antimicrobial CgA-derived peptides.

**Methodology/Principal Findings:**

PMNs were treated with different concentrations of CgA-derived peptides in presence of several drugs. Calcium mobilization was observed by using flow cytometry and calcium imaging experiments. Immunocytochemistry and confocal microscopy have shown the intracellular localization of the peptides. The calmodulin-binding and iPLA2 activating properties of the peptides were shown by Surface Plasmon Resonance and iPLA2 activity assays. Finally, a proteomic analysis of the material released after PMNs treatment with CgA-derived peptides was performed by using HPLC and Nano-LC MS-MS. By using flow cytometry we first observed that after 15 s, in presence of extracellular calcium, Chromofungin (CHR) or Catestatin (CAT) induce a concentration-dependent transient increase of intracellular calcium. In contrast, in absence of extra cellular calcium the peptides are unable to induce calcium depletion from the stores after 10 minutes exposure. Treatment with 2-APB (2-aminoethoxydiphenyl borate), a store operated channels (SOCs) blocker, inhibits completely the calcium entry, as shown by calcium imaging. We also showed that they activate iPLA2 as the two CaM-binding factors (W7 and CMZ) and that the two sequences can be aligned with the two CaM-binding domains reported for iPLA2. We finally analyzed by HPLC and Nano-LC MS-MS the material released by PMNs following stimulation by CHR and CAT. We characterized several factors important for inflammation and innate immunity.

**Conclusions/Significance:**

For the first time, we demonstrate that CHR and CAT, penetrate into PMNs, inducing extracellular calcium entry by a CaM-regulated iPLA2 pathway. Our study highlights the role of two CgA-derived peptides in the active communication between neuroendocrine and immune systems.

## Introduction

Chromogranin A (CgA) is a well-studied member of the chromogranin/secretogranin family, present in secretory cells of the nervous, endocrine and immune systems [1]. CgA was the first chromogranin to be characterized as an acidic protein co-stored and co-released with the catecholamine hormones from the chromaffin cells of the adrenal medulla. The discovery that Pancreastatin, a CgA-derived peptide (bCGA_248–293_) was able to inhibit the glucose-evoked insulin secretion from pancreatic beta-cells [2] initiated the concept of a prohormone function for this protein [3]. Numerous endogenous cleavage products of CgA have since been identified in the intragranular matrix of chromaffin cells, resulting from the proteolytic digestion at 13 sites [4] by intragranular enzymes, such as prohormone convertases PC1/3, PC2, neuroendocrine-specific carboxypeptidase E/H, Lys and Arg-aminopeptidases [5]. Among the CGA derived fragments, several induce biological activities [1] an their *in vitro* actions strongly suggest involvement in homeostatic processes, such as calcium and glucose metabolisms [6], cardiovascular functions [7]–[11], inflammatory reactions [12], [13], pain relief, tissue repair [14], gastrointestinal motility [15], [16] and in the first line of defence against invading microorganisms [17]–[19]. The possible implication of CgA and some of its derived-peptides in human diseases has also been reviewed [20], [21].

We have identified a range of antimicrobial peptides deriving from the natural processing, not only of CgA but also Chromogranin B, Proenkephalin-A and Ubiquitin co-secreted with catecholamines upon stimulation of chromaffin cells from the adrenal medulla [17], [18], [22]–[25]. These new antimicrobial peptides are integrated in the concept that the adrenal medulla plays an important role in innate immunity [26]. Furthermore, when polymorphonuclear neutrophils (PMNs), known to accumulate at sites of inflammation are stimulated by lipopolysaccharide or other bacterial agents, such as Panton-Valentine leucocidin (PVL) [27], [28], these cells produce and secrete intact and processed forms of CgA, such as Vasostatin-I and -II (residues 1–76 and 1–113) [18] and Cateslytin (residues 344–358) [17], [29]–[31], the N-terminal fragment of Catestatin (residues 344–364) [32]. In view of the established function of PMNs as central effectors cells in innate immune responses to inflammatory stimuli, it is of a great importance to understand the implications of the production and secretion of CgA-derived peptides for the regulation of PMNs responses to external stimuli.

In the present study, we have investigated the effects of two of the potent antimicrobial CgA-derived peptides on activation of PMNs release, *i.e.*: Chromofungin (CHR, bovine/human CgA_47–66_) [24] and Catestatin (CAT, bovine CgA_344–364_) [17]. By use of biochemical techniques, confocal microscopy, flow cytometry, calcium imaging, surface plasmon resonance and proteomic analyses, these two CgA peptides have now been demonstrated to stimulate exocytosis from PMNs by provoking a transient Ca^2+^ entry independent of release from internal stores. The mechanism by which CHR and CAT may induce Ca^2+^ influx appears to involve calmodulin (CaM) binding. The subsequent activation of the calcium-independent phospholipase A2 (iPLA2) in the complex regulation of store-operated channels (SOCs) [33]–[37], seemingly potentiates the release of a range of PMNs factors of importance identified by use of nanoLC-MS/MS.

## Results

Since PMNs are central effectors in innate immune response to inflammatory stimuli, we investigated the effects induced by two highly conserved antimicrobial CgA derived peptides on these cells. Chromofungin (CHR, RILSILRHQNLLKELQDLAL) corresponds to the active antifungal domain CgA_47–66_ of bovine and human Vasostatin-1 CgA_1–76_ [24] while Catestatin (CAT, RSMRLSFRARGYGFRGPGLQL) corresponds to a potent antimicrobial peptide CgA_344–364_ conserved in both cattle and man. These two peptides are not lytic for mammalian cells and display antimicrobial activities at the micromolar range [17], [24].

### CHR and CAT provoked Ca^2+^ entry in PMNs

**Figure 1:**
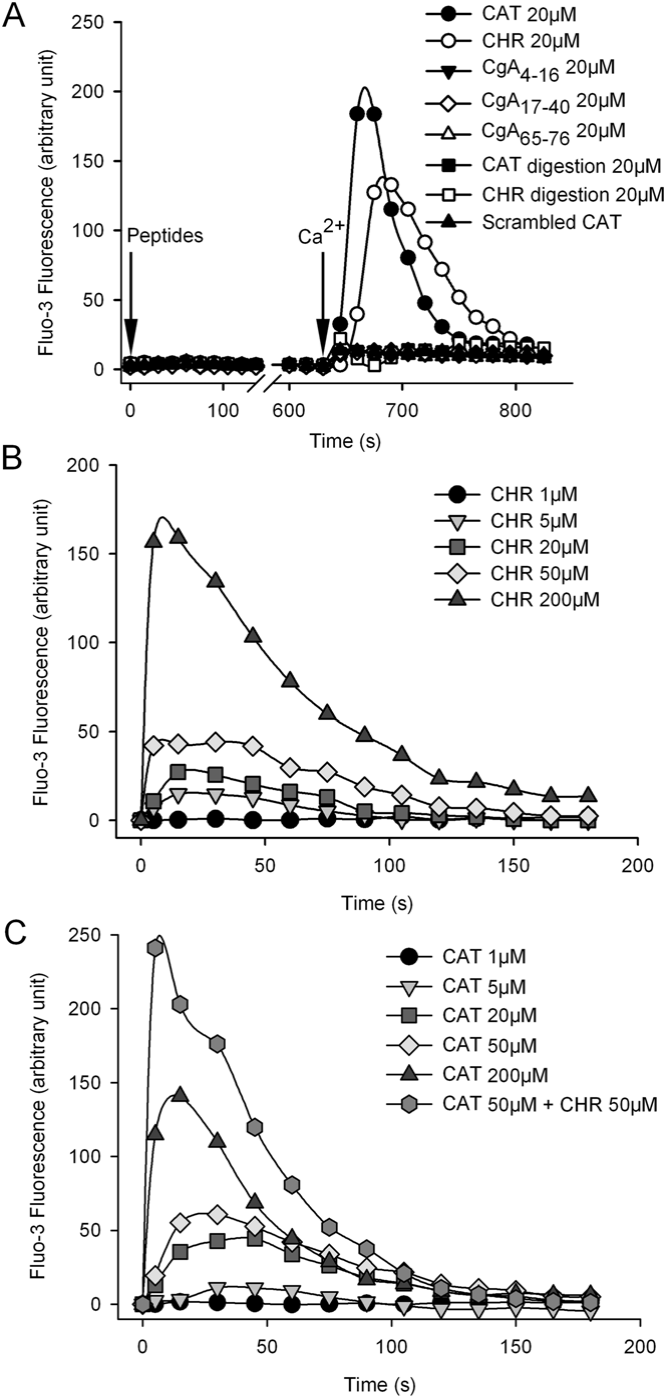
Ca^2+^ influx evoked in human PMNs by CGA-derived peptides. For flow cytometry analysis, human PMNs (5.10^5^ cells/ml) were loaded with Fluo-3AM probe (5 µM) and its fluorescence intensity of Fluo-3 was monitored at λ_Em530 nm_. Traces were obtained from 3000 PMNs and are averaged from triplicates at least. Peptides were added in the Ca^2+^-free EGTA buffer at t = 0 and 1.1 mM CaCl_2_ at t = 620 s. A) Time course variations of the intracellular Ca^2+^ in human PMNs in response to 20 µM CAT or 20 µM CHR and compared with other CgA-derived peptides (20 µM of CgA_4–16_, CgA_17–40_ or CgA_65–76_), tryptic digests of either 20 µM CAT, CHR or scrambled CAT. B, C) Time course variations of the intracellular Ca^2+^ in human PMNs in response to increasing concentrations (1–200 µM) of CHR (B), CAT (C) or a mixture of 50 µM CAT and 50 µM CHR (C).

In PMNs loaded with Fluo-3, a transient Ca^2+^ influx was induced by 20 µM of either CHR or CAT (20 µM) in the presence, but not in the absence, of 1 mM free extracellular Ca^2+^ (Figure 1A). Several other CgA-derived peptides (20 µM) were compared with CHR and CAT for influx of Ca^2+^. Three peptides derived from the N-terminal CgA domain failed to affect the Ca^2+^ influx (Figure 1A). These peptides were: CgA_4–16_ (NSPMNKGDTEVMK), the disulfide loop CgA_17–40_ (CIVEVISDTLSKPSPMPVSKEC) and the natural peptide CgA_65–76_ (ALQGAKERTHQQ) previously reported to be without antimicrobial activities [5]. Furthermore, mixtures of peptides resulting from the tryptic digest of CHR (R, ILSILR, HQNLLK, ELQDLAL) and CAT (SSMK, LSFR, ARAYGFR, GPGPQL) were without effect on Ca^2+^ influx^,^ nor was scrambled CAT peptide (SLPRRQLPSSAGMRGGKFAYF) of any effect (Figure 1A). Hence, the complete sequences of CHR and CAT appear to be essential for the induction of the transient Ca^2+^ influx.

The inducing effects of CHR and CAT on the Ca^2+^ influx were concentration-dependent in the 5–200 µM range (Figure 1B, C). Interestingly, the simultaneous addition of CHR and CAT at equal concentrations provoked a higher Ca^2+^ increase (Figure 1C) than the sum of effects observed by separately added peptides (Figure 1B and C), suggesting that CHR and CAT may have synergic effects on the transient Ca^2+^ entry in PMNs.

**Figure 2:**
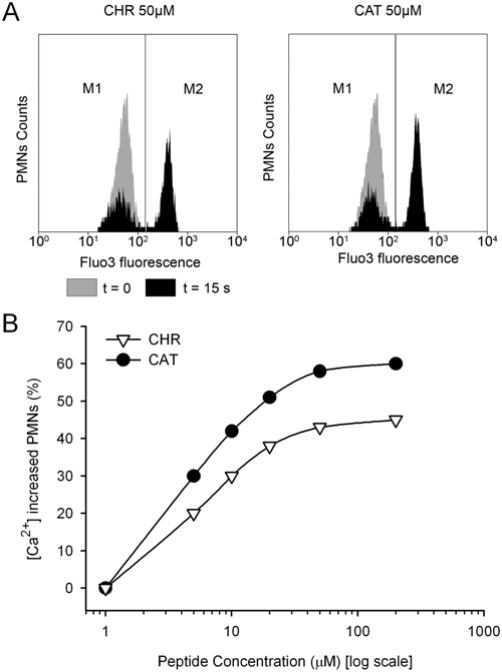
Concentration-dependent Ca^2+^-influx after stimulation of human PMNs by CHR and CAT. A) Representative diagrams of flow cytometry determination of the Ca^2+^ influx in the basic (M1) and peptide-activated (M2) windows, respectively. These influxes (in black) were obtained 15 s after the addition of 50 µM CHR or 50 µM CAT at t = 0 (in grey). PMNs activated by peptide-induced Ca^2+^ influx correspond to the M2 population. B) Flow cytometry quantification of PMNs in the M2 window in percent of the total initial population of PMNs, after 15 s exposure to increasing concentrations of CHR or CAT (1–200 µM).

An immediate increase of activated PMNs (M1/M2) was observed 15 s after the application of either CHR or CAT (Figure 2A). With different concentrations of CHR or CAT (0–200 µM), the percentage of activated PMNs was concentration dependent, being maximal at 50 µM for CHR (45%) and CAT (60%), respectively (Figure 2B).

### Ca^2+^ entry evoked by CHR and CAT via Ca^2+^ selective Store Operated Calcium channels (SOCs)

**Figure 3:**
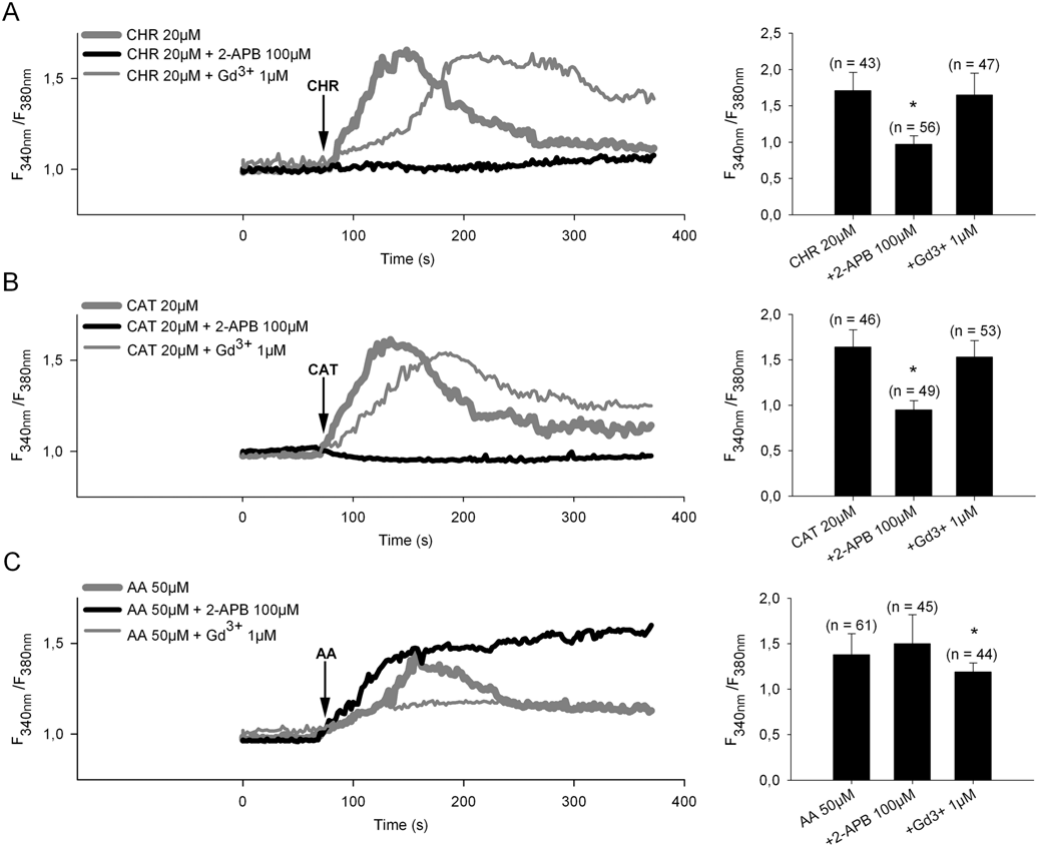
Ca^2+^ influx in PMNs induced by CHR, and CAT and arachidonic acid (AA). Time-lapse intracellular calcium imaging was performed on cells loaded with 1 µM Fura-2-AM and then further treated with 20 µM CHR, 20 µM CAT and 50 µM (AA) in Hepes-buffered Krebs medium in presence of 2.5 mM CaCl_2_. A–C) Left panels, Representative traces showing average change in [Ca^2+^]_i_ (F_340nm_/F_380nm_) recorded in the ratio mode from individual PMNs loaded with Fura-2. The arrow indicates the time of addition of 20 µM CHR, 20 µM CAT and 50 µM AA. In separate assays (as indicated) either 100 µM 2-aminoethyl diphenylborinate (2-APB) or 1 µM gadolinium chloride (Gd^3+^) were applied 2 min before addition of peptides or AA. Traces are averaged from triplicates. Right panels, Summary of data in left panels, showing the maximal Ca^2+^ influx (mean ratio±S.E.) obtained with the different conditions as indicated below bars. (n), number of PMNs used for each experiment; *, indicate significant differences (*p*<0.05), significance for difference from effect of CHR, CAT or AA alone.

Pretreatment of PMNs with the specific blocker of SOCs, 2-aminoethyl diphenylborinate (2-APB) for 2 min before addition of peptides, completely blocked the entry of extracellular Ca^2+^ by CHR or CAT (Figure 3A and 3B), suggesting that the two peptides were involved in the activation of Store Operated Calcium Entry (SOCE) through SOCs. Ca^2+^ entry in response to CHR and CAT was also compared to that induced by Arachidonic Acid (AA), acting through the Arachidonic-Regulated Channels (ARCs) (Fig 3C). The calcium influx induced by 50 µM AA was insensitive to 2-APB, while inhibited by gadolinium chloride (Gd^3+^), in marked contrast to the influx induced by CHR or CAT (Figure 3A–C). Thus, the Ca^2+^- entry induced by the two CgA-derived peptides was pharmacologically distinct from that by AA.

**Figure 4:**
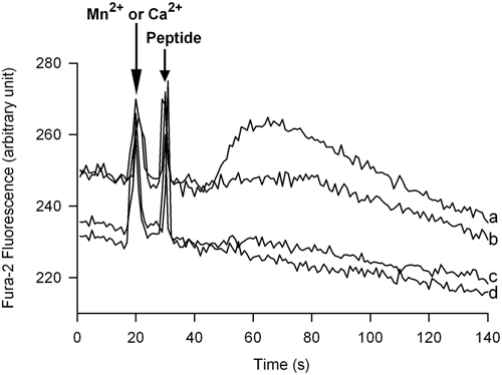
Specificity of the Ca^2+^ entry provoked by 50 µM CHR and 20 µM CAT. Characterization by spectrofluorimetry of the specificity of the Ca^2+^ entry induced by 50 µM CHR or 20 µM CAT for comparison with peptide effects on Mn^2+^ entry. Simultaneous recordings of fluorescent intensity variations of PMNs loaded with Fura-2 in the presence of 1 mM CaCl_2_ (a, b) and 0.2 mM MnCl_2_ (c, d) at the corresponding wavelengths of excitation λ_Ex340 nm_ and λ_Ex360 nm_. Divalent cations and peptides were added as indicated by arrows. Recordings are representative of three experiments.

Finally, the specificity for divalent cation entry into PMNs loaded with Fura-2 was assessed by comparison of the peptide induced Ca^2+^ entry (traces a and b) with that of Mn^2+^ (traces c and d) at the corresponding wavelengths of excitation (λ_ex340nm_ and λ_ex360nm_) (Figure 4). By comparison of the traces for CAT (a, c) and CHR (b,d) there was no evidence for Mn^2+^ entry in response to either peptide, consistent with activation of SOCs selective for Ca^2+^ and non-permeable for Mn^2+^ in PMNs [38].

### Calmodulin binding of CHR and CAT

**Figure 5:**
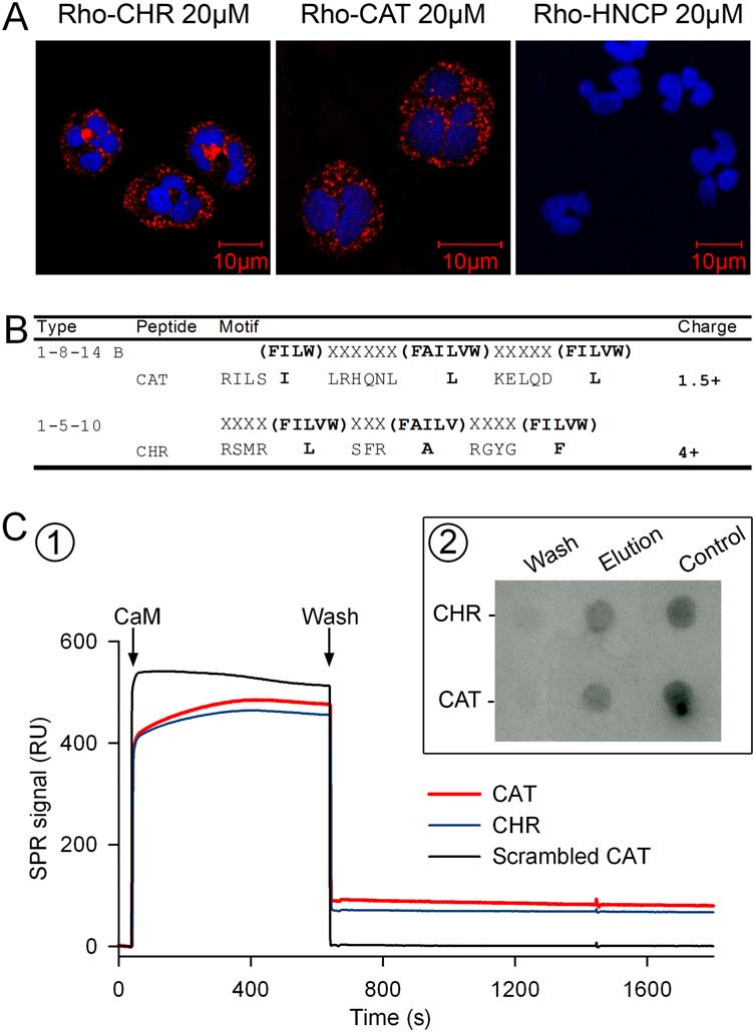
Interaction of CHR and CAT with calmodulin (CaM). A) Fluorescence confocal microscopy of PMNs after incubation during 120 s with 20 µM rhodamine-labeled peptides (Rho-CHR or Rho-CAT). Control peptide: 20 µM rhodamine labeled Hippocampal Cholinergic Neurostimulating Peptide (Rho-HCNP). The PMNs nuclei were labeled with Draq-5. B) Alignment of the CHR and CAT sequences with two CaM binding motifs 1-8-14 B and 1-5-10. The alignment has been obtained manually so that hydrophobic residues occupy invariant positions shown with boldface letters. The net positive charge is also indicated. C) (1) Interactions between CaM, CAT and CHR by Surface Plasmon Resonance (SPR) Peptides were immobilized on a CM5 Biacore chip using amine-coupling chemistry. CaM (5 µM) was added at arrow and allowed to stabilize for 10 min. Dissociation by washing was recorded for the following 10 min. All measurements were performed at 20°C. The resulting sensorgrams were analyzed using BIAevaluation Software. (2) Calmodulin-Affinity Chromatography of CHR and CAT. The retained and eluted peptides were immunodetected by dot blot with anti-CHR (monoclonal anti-CgA_47–68_) and anti-CAT (polyclonal anti-CgA_344–364_). Wash (5^th^ wash after adsorption of peptides).

The uptake of rhodamine-labeled CHR and CAT into isolated PMNs was demonstrated by confocal microscopy (Figure 5A). After 2 min incubation, the fluorescent rhodamine-labeled peptides were detected in the cytoplasm. In contrast, the control peptide, rhodamine-labeled Hippocampal Cholinergic Neurostimulating Peptide (HCNP), unable to induce Ca^2+^ influx into PMNs (not shown), was not detected in the cytoplasm, indicating a specificity for the entry of rhodamine-labeled CHR and CAT (Figure 5A).

The two cationic sequences CAT and CHR may be aligned with two Ca^2+^- dependent CaM binding motifs 1-8-14 B and 1-5-10 [39], as illustrated in Figure 5 B and previously demonstrated for binding of CHR to CaM [24], [40]. Interactions between CaM, CAT and CHR were assessed by surface plasmon resonance (SPR) (Figure 5 C1), revealing that not only CHR but also CAT specifically interacted with CaM, while scrambled CAT was inactive. In addition, CaM interacts with both CHR and CAT (Figure 5C2) when assayed by CaM-binding affinity chromatography and dot blot immunodetection of CgA in the fixed material with anti-CHR (monoclonal anti-CGA_47–66_) [14] and anti-CAT (polyclonal anti-CGA_344–364_) [17].

### Role of iPLA_2_ in the Ca^2+^ entry evoked by CHR and CAT

**Figure 6:**
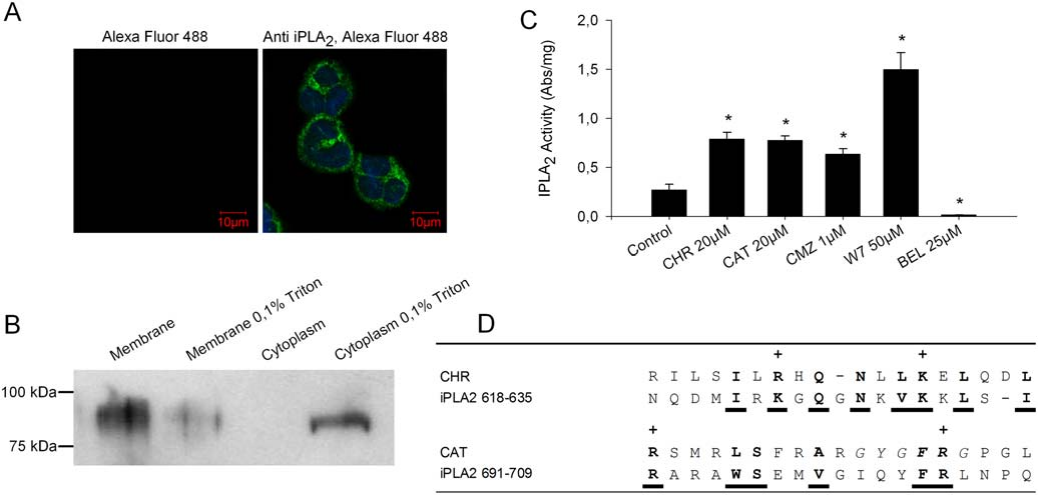
CHR and CAT stimulate iPLA2 activity. A) Fluorescence confocal microscopy of PMNs: Left, PMNs treated only with the secondary antibody (Alexa Fluo 488-conjugated donkey anti-rabbit IgG dilution 1∶2000). Right, PMNs stained with polyclonal anti-iPLA_2_ as primary antibody before Alexa Fluo 488 conjugated donkey anti-rabbit IgG. B) Western-blot analysis: The presence of a 82 kDa protein corresponding to iPLA_2_ was immunodetected in the membrane fraction obtained from PMNs. A comparable signal was obtained from the supernatant fraction of membranes treated with 0.1% Triton X-100. C) iPLA_2_ activity assay: Stimulation of iPLA_2_ activity after treatment by 20 µM CHR or 20 µM CAT for 30 min at 37°C and comparison with the effects of two CaM-binding factors (1 µM CMZ, 50 µM W7) and one iPLA_2_ inhibitor (25 µM BEL). Activity of iPLA_2_ is expressed as the absorbance (Abs/mg of protein) at λ_405 nm_. Results from two similar experiments, each performed in triplicate are presented as mean±S.E. * p<0.05, significance for difference from controls. D) Alignment of CHR and CAT sequences with two CaM-binding peptides of iPLA_2_. CHR sequence has been aligned with the iPLA_2_ CaM-binding motif aa 618 to aa 635 while the CAT sequence has been aligned with the iPLA_2_ CaM-binding motif (691–709). Corresponding homologous residues are in bold and underlined fonts.

Previous studies have demonstrated that functional iPLA_2_ is involved in activation of SOCs from different cell types [37] and that displacement of inhibitory CaM from iPLA_2_ may activate iPLA_2_, subsequently generating of lysophospholipids for activation of SOCs [36]. Confocal microscopy confirmed the presence of immunoreactive iPLA_2_ in PMNs (Figure 6A, right panel). The membrane localization of immunoreactive iPLA_2_ could be verified after subcellular fractionation, Triton X-100 treatment and western blotting (Figure 6B, [36]). The enzymatic activity of iPLA_2_ in PMNs was stimulated not only by two CaM inhibitors, *i.e.* calmidazolium (CMZ) and N-(6-aminohexyl)-5-chloro-l-naphthalenesulfonamide (W7), but also by CHR and CAT (Figure 6C). Interestingly, a comparison of CHR and CAT sequences with two CaM-binding peptides of iPLA_2_ reveals marked homologies (Figure 6D). Of note, the CHR sequence may be aligned with one of the iPLA_2_ CaM-binding motif (iPLA2_618–635_), while the CAT sequence aligns with another, *i.e.* the iPLA_2_ CaM-binding motif (iPLA2_691–709_) [41].

### CHR and CAT directly activate SOCs via iPLA_2_

**Figure 7:**
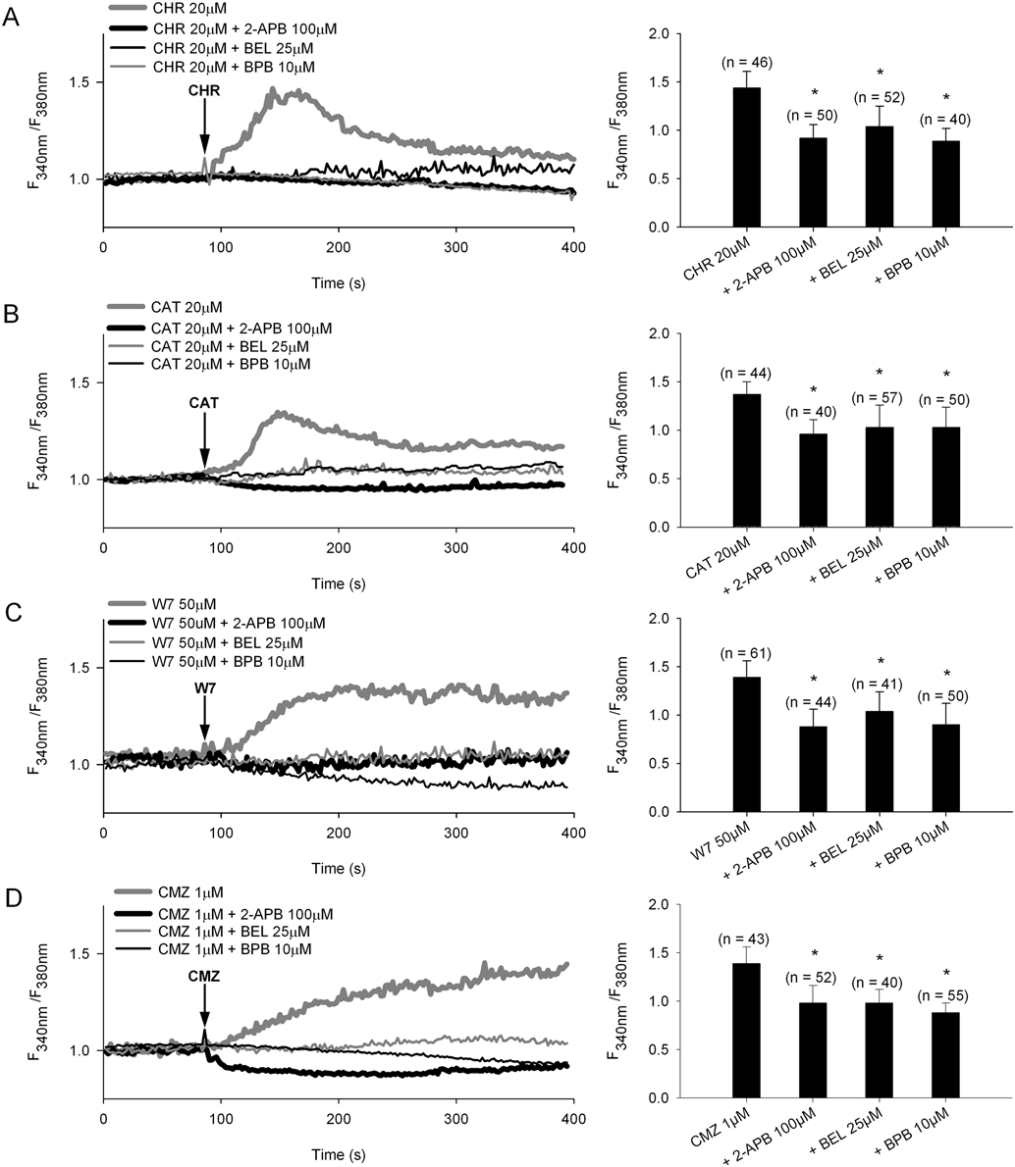
Involvement of iPLA_2_ in Ca^2+^ entry evoked by CHR and CAT in PMNs. A–D) Left panels: Effects of the SOC blocker (100 µM 2-APB) and inhibitors of iPLA_2_ (25 µM BEL) and PLA_2_ (10 µM BPB) on Ca^2+^ entry evoked by 20 µM of CHR or CAT, and two CaM antagonists (30 µM W7 and 1 µM CMZ). The representative traces show the average change in intracellular Ca^2+^ concentration (Fura-2 ratio, F_340_/F_380_) recorded simultaneously in a number of individual PMNs at 37°C. The following inhibitors were added: BEL (30 min), BPB (10 min) and 100 µM 2-APB (2 min) before addition of peptides or CaM antagonists (at arrows). Results are obtained from at least three independent experiments. Right panels: Bars represent the maximum Ca^2+^ influx (Ratio±S.E.) in different treatments (indicated below each bar); (n) number of PMNs; * *p*<0.05, significance for difference from peptide or CaM antagonist alone.

Two inhibitors of iPLA_2_ were compared with 2-APB for effects on peptide inducing Ca^2+^ influx (Figure 6C, 7A, B). Bromoenol lactone (BEL), a specific inhibitor of iPLA_2_ and bromophenacyl bromide (BPB), an inhibitor of PLA_2_, abolished the transient Ca^2+^ influx induced by CHR or CAT similarly to 2-APB. In addition, the two CaM antagonists W7 and CMZ induced longer lasting rises in influx (Figure 7C, D), that were significantly suppressed by the PLA_2_ inhibitors BEL and BPB and the specific SOC blocker 2-APB.

Interestingly, CMZ and some CaM inhibitory peptides may directly activate SOCs without the release of intracellular Ca^2+^ stores [42]–[44]. Hence, these findings indicate that CAT and CHR, being unable to evoke a Ca^2+^ mobilization from intracellular stores in absence of extracellular Ca^2+^, by their 2-APB sensitive induction of Ca^2+^ entry *via* SOCs may converge on iPLA_2_ in a manner similar to the two CaM inhibitors W7 and CMZ.

### Proteomic analysis of the PMNs secretions released by CHR and CAT

**Figure 8:**
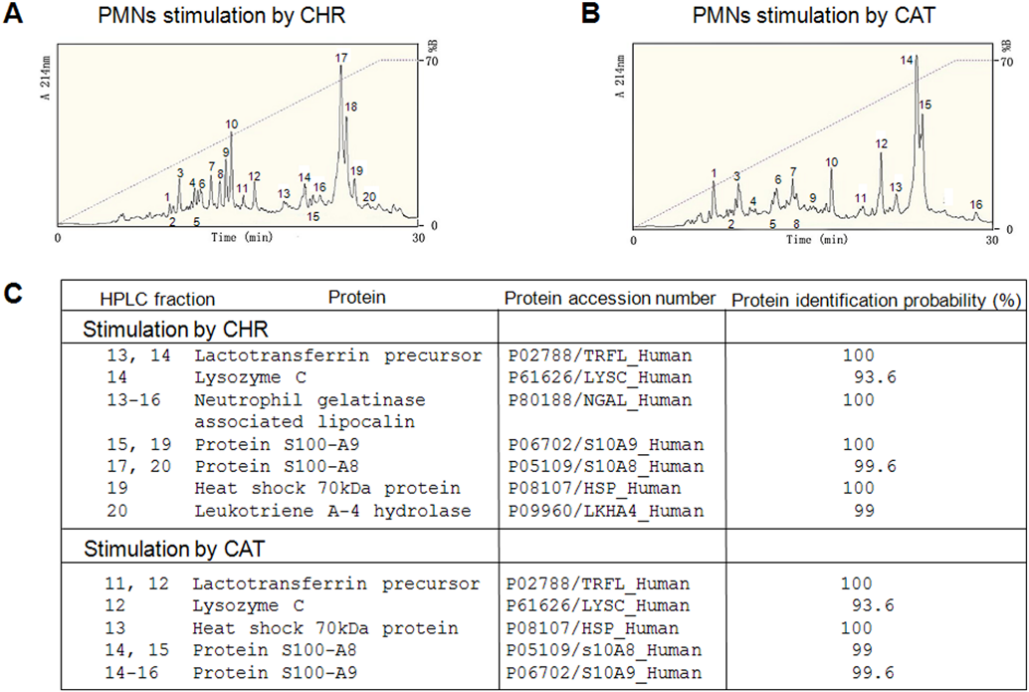
HPLC of proteins in PMNs secretions induced by CHR and CAT and implication for innate immunity. A, B) Secretion from PMNs (1.10^8^ cells) was induced during 30 min stimulation by (A) 20 µM CHR or (B) 20 µM CAT. The secreted proteins >3 kDa were purified by RP-HPLC on a Macherey Nagel reverse-phase C18 column (4×250 mm; particle size 5 µM and pore size 100 nm). Numbered peaks in the chromatograms indicate protein fractions subjected to proteomic analyses. (C) Proteomic identification by NanoLC-MS/MS analysis of protein fractions involved in innate immunity (protein identification probability >93%). The numbered HPLC fractions correspond to the peaks of secreted protein in the chromatograms presented in A and B, respectively.

PMNs secretions obtained after treatment with CHR and CAT were chromatographied by HPLC (Figure 8A, B) and the isolated fractions were identified by NanoLC-MS/MS analysis (supplementary data). Factors involved in innate immunity were identified among these proteins, *i.e.* lactotransferrin, lysozyme, neutrophil gelatinase associated lipocalin, S100 calcium binding protein A8, S100 calcium binding protein A9, heat shock 70 kDa protein and leukotriene A4 hydrolase (Figure 8C and complete data in the annexe with Figures S1 and S2).

CgA-derived fragments in the PMN secretions obtained after treatment with CHR and CAT were detected by western blotting, revealing traces of Vasostatin-1 and a CAT-including fragment (CgA_340–395_) (data not shown), consistent with the previously report of several CAT-including peptides with molecular weights in the range 70-15 kDa [17]. The major fragment corresponds to CgA_340–395_ (15–17 kDa) after stimulation of PMNs by CAT.

## Discussion

The present experiments are the first to demonstrate that not only CAT but also the distinctly different sequence CHR may play a role in Ca^2+^ signaling outside the chromaffin cells, *i.e.* serving as immunomodulators for activation of PMNs secretion.

A rapid and effective response to challenge with pathogens is essential for the survival of all living organisms. Natural cationic host defense peptides represent lead molecules that boost innate immune responses and selectively modulate pathogen-induced inflammatory responses [45]. Among the different mechanisms that have evolved to this effect, the production of a large variety of natural antimicrobial peptides has gained increasing attention.

CHR and CAT are two antimicrobial peptides derived from CgA. They are stored in secretory granules of chromaffin cells and PMNs in a larger form, which may be processed extracellularly to the mature active peptides [17], [18]. In the intragranular matrix, they result to the processing of CgA by numerous enzymes such as prohormone convertases (PC1 and 2), aminopeptidases and carboxypeptidases. In the extracellular medium, larger forms may be processed by kallikrein located at the plasmic membrane level and by circulatory proteolytic enzymes such as plasmin and thrombin [1]. In addition, some bacterial virulence factors are indeed proteolytic enzymes, *i.e.* Glu-C protease from *Staphylococcus aureus,* and might continue the natural processing of CgA to generate the CHR and CAT fragments during infections [17], [18], [46].

**Figure 9:**
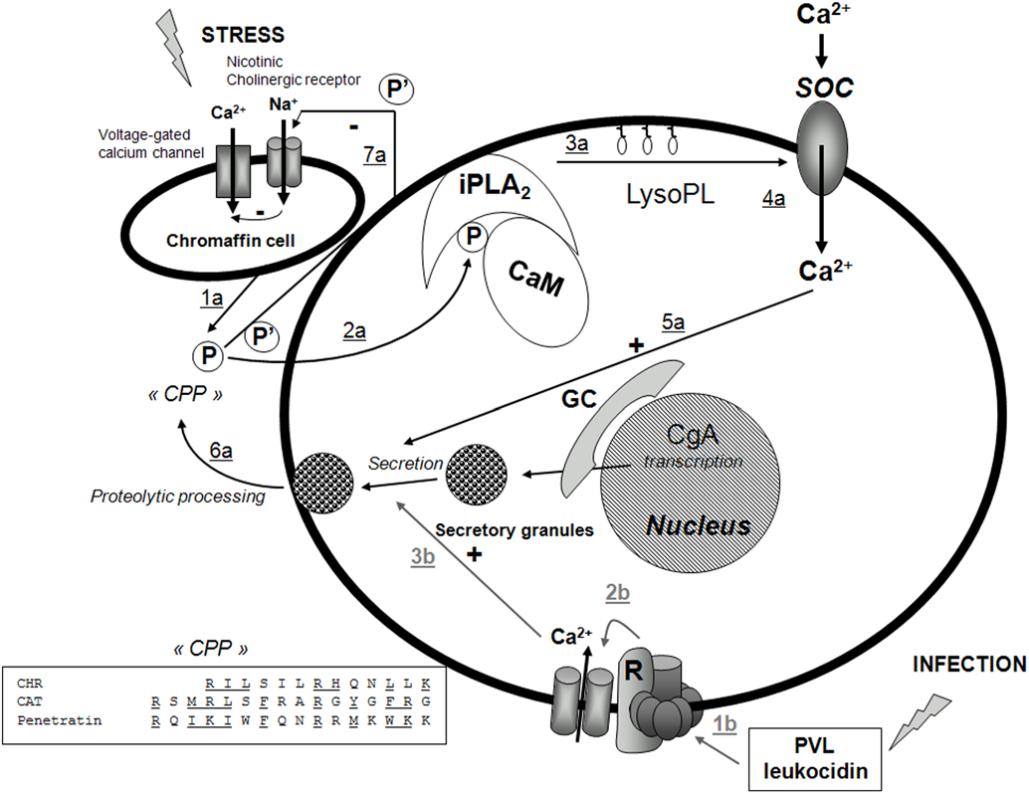
Model for the action of CHR and CAT on PMNs activation. Stress and infection lead to two different pathways for stimulation of PMN secretion, by release of CgA and CgA-derived peptides from the adrenal medulla, as indicated by 1a–6a (black), and by PVL leucocidin stimulation by *S. aureus infections*, as indicated by 1b–3b (grey), respectively. Abbreviated symbols: P (CHR, CAT), P' (CAT), CPP (Cell Penetrating Peptide), CaM (calmodulin), iPLA_2_ (calcium independent phospholipase A_2_) LysoPL (lysophospholipids); GC (Golgi complex); PVL (Panton-Valentine leucocidin), R (receptor). The stress-stimulated pathway leads to penetration of P into the cytoplasm (2a), resulting in removal of inhibitory CaM, activation of iPLA_2_ to produce LysoPL (3a) and activation of Ca^2+^ influx through SOC (4a), converging on activated docking of secretory granules (5a) and subsequent release of proteins of relevance for innate immunity (6a). The negative feedback induced by P' on nicotinic cholinergic receptor of chromaffin cell is also indicated (7a). The infective route leads to activation of the putative PVL receptor coupled to opening of Ca^2+^-channels and a rise in intracellular Ca^2+^ (3b) that converges on docking of secretory granules and subsequent secretion of proteins of relevance for innate immunity (6a). Transcription of CgA in response to stress and infection is also indicated. Insert.The alignment of the two P sequences (CHR and CAT) with that of Penetratin (Cell penetrating peptide, CPP).

To date only CAT has been reported to act *via* a classical surface receptor, *i.e.* the nicotinic acetylcholine receptor [32]. On the other hand, several 70–80 kDa membrane binding proteins have been reported for Vasostatin-I (CgA_1–76_) [47], [48]. Furthermore, the sequences of CHR and CAT may also be aligned with the cell penetrating peptide (CPP), Penetratin, *i.e.* (Figure 9 insert), pointing the possibility for CHR and CAT, as new members of the family of CPPs [49].

Calcium is an universal secondary messenger involved in many cellular signal transduction pathways, regulating crucial functions such as secretion, cell motility, proliferation and cell death. Increased in intracellular Ca^2+^ derives mainly from two sources: internal stores releasing Ca^2+^ into the cytosol and channels in the plasma membrane that open to allow external Ca^2+^ to flow into the cell. In PMNs, calcium signaling has been reported to be involved in oxidase activation, cell degranulation and priming response to a wide variety of proinflammatory molecules [35]. The present experiments have demonstrated that the two CgA peptides, CHR and CAT, penetrate rapidly into PMNs (Figure 5A) and subsequently interact with CaM (Figure 5C) to activate iPLA_2_ (Figure 6C). Displacement of CaM leads to iPLA_2_ activation and production of lysophospholipids inducing the opening of SOCs [36], the entry of Ca^2+^ and the release of innate immune factors among a long list of proteins (Figure 8C and Figures S1 and S2).

The SOCE pathway remains one of the most intriguing and long lasting mysteries of Ca^2+^ signaling [43]. The main question concerns the origin of the signal [43], [50]: how does depletion of the internal Ca^2+^ store activate Ca^2+^ entry through the SOC in the plasma membrane?. The Calcium Influx Factor (CIF) is a specific factor produced by the cells following depletion of Ca^2+^ stores [51] and corresponds to the diffusible messenger that is produced by Ca^2+^ stores upon their depletion traveling to the plasma membrane to activate SOCs and Ca^2+^ entry [43]. The mechanism by CIF may activate SOCs with a displacement of CaM from the inactive complex with iPLA_2_. The subsequent formation of lysophospholipids and the opening of SOCs, consistent with an activation of SOCs by displacement of inhibitory CAM from the inactive complex with iPLA_2_ [36], [37], [43]. Thus, CaM inhibitors such as chemical substances and peptides, may activate SOCs by interfering with the CaM-iPLA_2_ complex [36], [43]. The present experiments have made evident that CHR and CAT induce a 2-APB sensitive Ca^2+^ entry without depletion of Ca^2+^ stores in a similar manner to CaM inhibitors, pointing to iPLA_2_, as the most likely target for these two peptides, once penetrated into the cytoplasm.

Of note, CAT is an inhibitory peptide controlling catecholamine release from chromaffin cells and noradrenergic neurons by an autocrine regulatory mechanism, non-competitively blocking the influx of Ca^2+^ coupled to activation of the nicotinic acetylcholine receptor, as illustrated in Figure 9, but not when triggered by membrane depolarization [32].

Several proteins involved in innate immunity (lactotransferrin, heat shock 70 kDa protein, lysosyme, neutrophil gelatinase associated lipocalin, protein S100-A8, protein S100-A9 and leukotriene A-4 hydrolase were identified by using proteomic analysis in the secretions of neutrophils stimulated by CHR and CAT (Figure 8C). In neutrophils, azurophil, specific and gelatinase granules contain host defense proteins in their luminal spaces. The extent of mobilization of the three types of granules depends on the stimulus intensity: gelatinase granules are most easily mobilized followed by specific granules and then azurophil granules.

Lactotransferrin is an iron-binding protein that is released from activated neutrophils at sites of inflammation by the three types of granules [52] and has antimicrobial, as well as anti-inflammatory properties [53]. Lactotransferrin serves as an autoregulator to retain PMNs at inflammatory sites [54] and it was able to delay spontaneous apoptosis of neutrophils [55]. The anti-inflammatory activity of lactotransferrin is due to binding and neutralization of proinflammatory molecules and its capacity to promote activation of neutrophils and macrophages [56]. Lysozyme is also released by three types of granules [52]. In addition to enzymatic lysis of bacterial cell wall, it can also kill bacteria by a non-enzymatic mechanisms [57]. Neutrophil gelatinase associated lipocalin (NGAL) was found in specific granules of neutrophils [58], [59]. It is involved in the allosteric activation of matrix-metalloproteinase (MMP)-9 and protection from degradation. It may function as an effector molecule of innate immune system [58], [60].

Proteins S100-A8 and A9, two Ca^2+^-binding proteins of the S100 family are secreted from neutrophils by the three types of granules as a heterodimeric complex (S100-A8/S100-A9) [61]–[63]. High levels of the proteins have been found in the extra-cellular milieu during inflammatory conditions [64], [65]. The S100-A8/A9 heterodimer is an antimicrobial complex that exhibits cytokine-like functions in the inflammatory sites *via* activation of NFkappa B and p38MAPK [66]. Hsp70, an ubiquitous family of protein chaperones that assist in folding of newly synthesized peptides and translocation of proteins across biological membranes, plays regulatory roles in signal transduction, cell cycle and apoptosis [67]. Hsp70 is known to contribute to the mechanisms of cell protection against a variety of stress and cytotoxic factors, providing an increase of cell survival [68]. Leukotriene A-4 hydrolase is released by the specific granules of neutrophils. It catalyzes the final and commited step in the biosynthesis of leukotriene B4, a potent chemotactic agent for neutrophils, eosinophils, monocyted and T cells that play key roles in the innate immune response [69].

A considerable number of studies have demonstrated that the neuroendocrine and immune systems communicate to promote reciprocal regulation in the host. Not only may immune cells sense pathogens and secrete proteins that modify cells of the neuroendocrine system but factors secreted by the neuroendocrine system may also bring about changes in immune cell activity [26].

In addition to CAT, substance P is another neuro-immunomodulator that may inhibit nicotinic cholinergic-stimulated catecholamine release, but displays a lower potency [70]. PACAP displays numerous functions in cells of neuronal and non neuronal origin and functional VIP/PACAP receptors have been demonstrated on PMNs, mediating Ca^2+^- dependent pro-inflammatory activities through the activation of multiple regulatory pathways [71]. Thus, it was demonstrated that the extracellular and intracellular Ca^2+^ play key roles in PACAP mediated proinflammatory activities [72]. In addition, it has been reported that, in contrast to the inhibitory effect of CAT, PACAP may induce an increase of Ca^2+^ entry and catecholamine release in bovine chromaffin cells by a a unique pathway distinct from nAChR, VOCs and SOCs [73]. Hence, in contrast to the action of PACAP, CAT may control the proinflammatory activity *via* a retroactive loop, inhibiting the adrenal medulla by an autocrine mechanism while enhancing PMNs secretions of a range of immunoactive factors.

To conclude, the data reported here are important for our understanding of the role for CgA in innate immunity and for its crucial role as a mediator in the cross-talk between the neuroendocrine and immune systems. All together, the immunomodulatory activities of CHR and CAT may be important for the design of pharmacological strategies to maintain homeostasis during recruitment of PMNs in microbial and systemic infections.

## Materials and Methods

### Peptide synthesis

All the synthetic peptides were prepared on an Applied Biosystems 433A peptide synthesizer (Foster City, USA), using the stepwise solid-phase approach with 9-fluorenylmethoxycarbonyl (Fmoc) chemistry. Then, the synthetic peptides were purified by RP-HPLC on a Macherey Nagel Nucleosil RP 300-7C18 column (10×250 mm; particle size 7 µm and pore size 100 nm). Rhodamine fluorophore 5(6)-carboxytetramethyl rhodamine was conjugated with peptides at the N-terminal extremity, as previously described [24]. Synthetic peptides were analysed by mass spectrometry and automated Edman sequencing on an Applied Sequencing System (Applied Biosystems, Foster City, USA) [5]. MALDI mass measurements were carried out on an Ultraflex™ TOF/TOF (Bruker Daltonics, USA) to perform a rapid control of synthetic peptides according to the procedure previously reported [74].

### Cells and flow cytometry measurements

Human PMNs were prepared from buffy coats purchased at the Etablissement Français du Sang – Strasbourg, France from anonymous and healthy donors from either sex. Briefly, 40 mL of a 1/3 (vol/vol) dilution of blood cells in 0.9% (wt/vol) NaCl was layered on 12 mL of 15 J Prep (Techgen International, Voisins le Bretonneux, France). After centrifugation at 800× *g* for 20 min, the pellet was suspended in 30 mL of 0.9% NaCl, added to 10 mL of 6% (wt/vol) dextran, and sedimented for 30 min. Thirty mL of the supernatant was centrifuged for 10 min at 800× *g*. The pellet was suspended in Hepes buffer (140 mM NaCl, 5 mM KCl, 10 mM glucose, 0.1 mM EGTA, 10 mM Hepes, 3 mM Tris; pH 7.3) and the contaminating erythrocytes were removed by hypotonic lysis for 45 s and subsequent washing in Hepes buffer [75]. The final suspension was adjusted to a concentration of 6.10^6^ PMNs/mL and 0.1% (Wt/vol) bovine serum albumin was added to prevent non-specific leucotoxin adherence on tube walls.

To flow cytometry measurements PMNs were suspended at a concentration of 5.10^5^ cells/mL in Hepes and measurements from 3000 PMNs were carried out using a FacSort® flow cytometer (Becton-Dickinson, Le Pont de Claix, France) equipped with a 15 mW argon laser tuned to 488 nm [76].

We evaluated the intracellular calcium, using flow cytometry of cells previously loaded with 5 µM Fluo-3 (Molecular Probes, New Brunswick, USA). The increase of Fluo-3 fluorescence was measured with different CgA-derived peptides in absence or after addition of 1.1 mM extracellular CaCl_2_ [77]. Fluo-3 fluorescence was measured from the fluorescence light 1 (FL1: λ_Em_ = 530 nm) using Cell Quest Pro™ software (Becton-Dickinson, Le Pont de Claix, France). Data provided are the average of three independent experiments of different cell preparations. Mean variations, mainly never exceed 10% of the measured values.

### Time-lapse intracellular Ca^2+^ imaging

PMNs were incubated for 30 min at 37°C in Hepes-buffered Krebs medium (130 mM NaCl, 4.5 mM KCl, 1.2 mM MgSO_4_, 2.5 mM CaCl_2_, 11 mM D-glucose, 10 mM Hepes, pH 7.4) with 2 µM Fura-2-AM. Cells were then washed with Hepes-buffered Krebs medium and subsequently incubated for 20 min to allow Fura-2-AM hydrolysis. After three additional washes the cells were placed on an inverted microscope (Axiovert 35M Zeiss, Germany), superfused with Krebs solution (1 mL/min), and alternatively illuminated at 350±10 nm and 380±10 nm. The fluorescent emission was observed using a long pass filter at 510 nm. For each excitation wavelength and every 2 s an image pair was recorded using a CCD camera (ImageEM™, Hamamatsu Photonics, Hamamatsu, Japan) and fluorescence ratio images were calculated subsequently using Metafluor™ software (Molecular Devices, Downingtown, USA).

### Spectrofluorometry determination

PMNs were loaded with Fura-2 as above and washed in EGTA buffer with 1.1 mM CaCl_2_ added. Continuous variations of fluorescence intensities were recorded using a dual excitation and dual emission spectrofluorometer Deltascan (Bioritek, PTI, Chamarande, France) with slits set at 4 nm. One mL of PMNs (4.10^6^ cells/mL) was added to 1 mL of the assay solution under constant stirring in a 4 ml cuve (1 cm light path) thermostated at 37°C. Wavelengths were settled at 340 nm (Ca ^2+^) and 360 nm (Mn^2+^) for excitation, and 510 nm for emission. Data were expressed in arbitrary units.

### Confocal microscopy analysis of rhodamine-labelled peptide loaded PMNs

PMNs (1.10^6^/mL) were incubated 2 min at room temperature (RT) with rhodamine-labelled peptides (20 µM) in Phosphate Buffered Saline consisting of 10.5 mM KH_2_PO_4,_ 30 mM Na_2_HPO_4_, 0.154 M NaCl, pH 7.4, washed and subsequently centrifuged by cytospins of 1–2.10^5^ isolated PMNs onto glass slides (300 rpm, 10 min, RT). Cytospins were fixed with 4% (v/v) paraformaldehyde in PBS at RT for 20 min, then incubated with Draq-5 (dilution1∶500, Biostatus Limited., Leicestershire, UK) for 15 min. Stained cells were monitored with a Zeiss LSM 510 laser scanning microscope (Zeiss, Iena, Germany) equipped with a Planapo oil immersion objective (×63, numerical aperture 1.4).

### Surface Plasmon Resonance experiments

Experiments were conducted on a Biacore 3000™ (GE Healthcare) and peptides were immobilized on a CM5 Biacore chip using amine-coupling [78]. The surface of the chip was activated for 7 min at a flow rate of 5 µL/min with a mixture of 50 mM NHS and 0.2 mM EDC. Peptides were covalently linked to the surface giving up to 2000 resonance units (RU). Ethanolamine (1M, pH8.5) was injected for 7 min to block the remaining activated groups. 5 µM CaM (Sigma, St. Louis, MO, USA) addition was performed at an injection flow of 20 µL/min in 50 mM NaCl, 2 mM CaCl_2_, 20 mM Tris pH6.5 until stabilization was reached (10 min). Dissociation was registered for 10 min after the end of injections. All measurements were performed at 20°C. Sensorgrams were analyzed by using BIAevaluation Software.

### Calmodulin-Affinity Chromatography and dotblot immunodetection

CHR and CAT were prepared in buffer A (50 mM NaCl, 2 mM CaCl2, 50 mM Tris-HCl, pH 7.5) and loaded on 0.1 ml CaM-Sepharose 4B column (GE healthcare, Buckinghamshire, United Kingdom) with molar ratio 10∶1. The mixtures were incubated at room temperature for 1 h. Afterwards, the column was washed (x5) with 1 mL of buffer A, and (x3) 0.1mL buffer B (50 mM NaCl and 2 mM EGTA, 50 mM Tris-HCl, pH 7.5) were used to elute the CaM binding peptides. The elutions were analyzed by dotblot onto PVDF membrane with pore 0.2 µM (Millepore, Billerica, MA, USA) with specific antibodies anti-CHR (monoclonal anti-CGA_47–68_ (dilution 1∶5,000) and anti-CAT (anti-CGA_344–364_ (dilution 1∶20,000) [17], [24]. Immunoreactivity signals were developed using goat anti-mouse IgG conjugated to peroxidase (dilution 1∶400,000, Jackson Immunoresearch laboratories, Baltimore Pike, USA).

### Immunocytochemistry of iPLA2 in PMNs

Cytospins were prepared by centrifugation of 1–2.10^5^ isolated PMNs onto glass slides (300 rpm, 10 min), then fixed with 4% (v/v) paraformaldehyde in PBS, at RT for 20 min, permeabilized with PBS containing 1% (v/v) Triton X-100 (Sigma, St. Louis, USA) at RT for 30 min, and blocked by incubation in PBS containing 0.25% (w/v) bovine serum albumin. Subsequently, cytospins were incubated with primary antibodies anti-iPLA_2_ polyclonal antibody (dilution 1∶100, Cayman chemical, Ann Arbor, USA) for 1 h at 37°C. Cytospins were washed with PBS, then incubated with secondary antibodies Alexa 488-conjugated donkey anti-rabbit IgG (dilution 1∶2000, Molecular Probes, Carlsbad, USA) during 45 min at 37°C, finally incubated with Draq-5 (dilution 1∶500) for 15 min. Immunofluorescence staining was monitored with a Zeiss LSM 510 laser scanning microscope equipped with a Planapo oil immersion objective (×63, numerical aperture 1.4).

### Detection of iPLA_2_ in PMNs and iPLA_2_ activity assay

#### Subcellular fractionation of PMNs

PMNs (5.10^7^/mL) were sonicated (10 W) in the absence or presence of 1% (v/v) Triton X-100, in the buffer 1 mM phenylmethysulphonyl fluoride, 1 mM EDTA, 50 mM Tris-HCl pH 7.5 for 3 times of 10 s with 15 s break between cycles, then spun down at 10,000× *g* for 15 min at 4°C. The pellet containing membranes was collected and the supernatant was further centrifuged at 100,000× g for 60 min at 4°C [44]. The amount of proteins contained in membrane and cytoplasm fractions was determined by Bradford method, and both preparations were stored at −20°C for later use.

#### Gel electrophoresis and Western-blot analysis of iPLA_2_ in PMNs

Fifty µg proteins of each sample were separated on 12% sodium dodecyl sulfate-polyacrylamide gel electrophoresis SDS-PAGE gradient gels (SDS-PAGE) and electrotransferred onto polyvinyldifluorene membranes (GE Healthcare Bioscience). IPLA_2_ were detected with a polyclonal anti-iPLA_2_ (dilution1∶500, Cayman Chemicals, Ann Arbor, MI, USA), and signal was developed using goat anti-rabbit IgG conjugated to peroxidase (dilution 1∶50,000, Jackson Immunoresearch Laboratories, Baltimore Pike, USA) to determine immunoreactivity [79].

#### iPLA_2_ activity assay

PMNs (3.10^8^) were collected, sonicated 3 times for 10 s with a 15 s break between cycles, and centrifuged at 15,000× *g* for 20 min at 4°C. The supernatant was removed and kept on ice. After determination of the protein concentration (Bradford assay), iPLA_2_ activity measurement was obtained in presence of different peptide concentrations using the cPLA_2_ assay kit, according to the modified method previously reported (cPLA_2_ assay kit, Caymen chemical), [36]. Results were from two similar experiments, each performed in triplicates.

### Characterization of secretions obtained after stimulation of PMNs by CHR and CAT

#### Stimulation and purification

PMNs secretory products were obtained after stimulation of 1.10^8^ PMNs with 20 µM CHR or 20 µM CAT in EGTA buffer for 20 min. PMNs were centrifuged at 800× *g* for 10 min at 4°C and the supernatant was recovered. Desalting and elimination of low molecular weights protein were achieved by centrifugation with a molecular cut-off 3 kDa Centricon (Pall, New York, USA), then the material was stored at −20°C for further analyses.

PMNs secretions induced by CHR and CAT were purified using a DIONEX Dual Gradient system (Dionex, Sunnyvale, USA) and a Nucleosil RP300–5C18 column (4×250 mm, 5 µm particle size, 300-Å porosity; Macherey-Nagel, Hoerdt, France). Absorbance was monitored at 214 nm, and the solvent system consisted of 0.1% (v/v) trifluoroacetic acid in water (solvent A) and 0.1% (v/v) trifluoroacetic acid in 70% (v/v) acetonitrile-water (solvent B with a flow rate of 1 mL/min. Gradient was indicated on chromatograms and fractions of 700 µL were collected.

#### Mass spectrometry analysis

The cysteine residues from the RP-HPLC fractions were reduced by 50 µL of 10 mM dithiothreitol at 57°C and alkylated by 50 µLof 55 mM iodoacetamide. The proteins were digested with 40 µL of 12.5 ng/µL of modified porcine trypsin (Promega, Madison, WI, USA) in 25 mM NH_4_HCO_3_ at 37°C for 14 h.

The generated peptides were analyzed directly by NanoLC-MS/MS on an Agilent 1100 Series HPLC-Chip/MS system (Agilent Technologies, Palo Alto, USA) coupled to an HCT Ultra ion trap (Bruker Daltonics, Bremen, Germany). The chip contained a Zorbax 300SB-C18 (43 mm×75 µm, with a 5 µm particle size) column and a Zorbax 300SB-C18 (40 nL, 5 µm) enrichment column. The solvent system consisted of 2% (v/v) acetonitrile, 0.1% (v/v) formic acid in water (solvent A) and 2% (v/v) water, 0.1% formic acid in acetonitrile (solvent B). The sample was loaded into the enrichment column at a flow rate set to 3.75 µL/min with solvent A. Elution was performed at a flow rate of 300 nL/min with a 8–40% linear gradient (solvent B) over 12 min and followed by a 70% stage (solvent B) over 5 min before the reconditioning of the column at 92% of solvent A. The complete system was fully controlled by ChemStation Rev. B.01.03 (Agilent Technologies).

All the analyses were performed using an HCT+ ion trap (Bruker Daltonics, Bremen Germany). The peptides were ionised by positive electrospray and fragmented by CID. The trap was externally calibrated with tunning mix for LC/MSD ion trap G2431A (Agilent Technologies). The voltage applied to the capillary cap was optimized to −1750 V. The MS scanning was performed in the standard enhanced resolution mode at a scan rate of 8.100 m/z per second. The mass range was 250 to 2500 m/z. The Ion Charge Control was 100000 and the maximum accumulation time was 200 ms. A total of 4 scans were averaged to obtain a MS spectrum and the rolling average was 2.

For tandem MS experiments, the system was operated with automatic switching between MS and MS/MS modes. The 3 most abundant peptides with an isolation width of 4 m/z, preferring doubly charged ions (absolute threshold of 2000 and a relative of 5%) were selected on each MS spectrum for further isolation and fragmentation. Ions were excluded after 2 spectra and released after 1 min.

Smart Parameters Setting was used for selected precursor ions. The MS/MS scanning was performed in the ultrascan resolution mode at a scan rate of 26.000 m/z per second. The mass range was 50 to 2800 m/z. The Ion Charge Control was 300000. A total of 6 scans were averaged to obtain a MS/MS spectrum. The values 30% and 200% give a start and end for fragmentation relative to the fragmentation amplitude selected (1.5 V).

The complete system was fully controlled by EsquireControl 6.1 Build 90 (Bruker Daltonics) software. Mass data collected during analysis were processed, converted into .mgf files using DataAnalysis 3.4 Build 179 (Bruker Daltonics) software. The standard parameters were used.

The smoothing algorithm was Savitzky Golay with a smoothing width of 0.2 m/z in 1 cycle. 250 compounds were selected with a threshold of 60000. The algorithm “Accu Mass” was used.

The MS and the MS/MS data were searched using a local Mascot server (version MASCOT 2. 2.0, MatrixScience, UK). Searches were performed with a mass tolerance of 200 ppm in MS mode and 0.3 Da in MS/MS mode and with the following parameters: trypsin specificity, one missed cleavage, cysteine carbamidomethylation and methionine oxidation (and protein amino-terminal acetylation) as variable modifications, trap fragmentation, without constraining protein molecular weight or isoelectric point and without any taxonomic restriction.

To minimize false positive identifications, the results were subjected to very stringent filtering criteria. For the identification of a protein with two peptides or more, at least two unique peptides had to a Mascot ion score above 20. In the case of single peptide hits, the score of the unique peptide must be greater than the 52. For the estimation of the false positive rate, a target-decoy database search was performed [80]. In this approach, peptides are matched against a database consisting of the native protein sequences found in the database (target) and of the sequence-reversed entries (decoy). The evaluations were performed using the peptide validation software Scaffold (Proteome Software, Portland, USA). This strategy was used to obtain a final catalogue of proteins with an estimated false positive rate below 1%.

### Drugs

Bromoenol Lactone (BEL), Thapsigargin (TG), Calmidazolium (CMZ), Bromophenacyl bromide (BPB), 2-Aminoethyl diphenylborinate (2-APB), N-6-aminohexyl-5-chloro-l-naphthalenesulfonamide (W7), Arachidonic acid (AA) and Rhodamine fluorophore, 5(6)-carboxytetramethyl rhodamine were purchased from Sigma (St. Louis, MO, USA).

### Statistical analysis

Group data are presented as mean±SD (as specified in the text and figure legends). Student's t test was used to determine the significance of data (*), *p*<0.05.

## Supporting Information

Figure S1 (0.30 MB XLS)

Figure S2 (0.41 MB PDF)
