# Supplementary material for: Two Chromogranin A-Derived Peptides Induce Calcium Entry in Human Neutrophils by Calmodulin-Regulated Calcium Independent Phospholipase A2
Source: PLoS One. 2009 Feb 19;4(2):e4501. doi: 10.1371/journal.pone.0004501 (PMC2639705; doi:10.1371/journal.pone.0004501)
Supplement: Figure S2 — (0.41 MB PDF) [file pone.0004501.s002.pdf]

Suplementary data

Spectrum from the protein identified with a unique peptide.

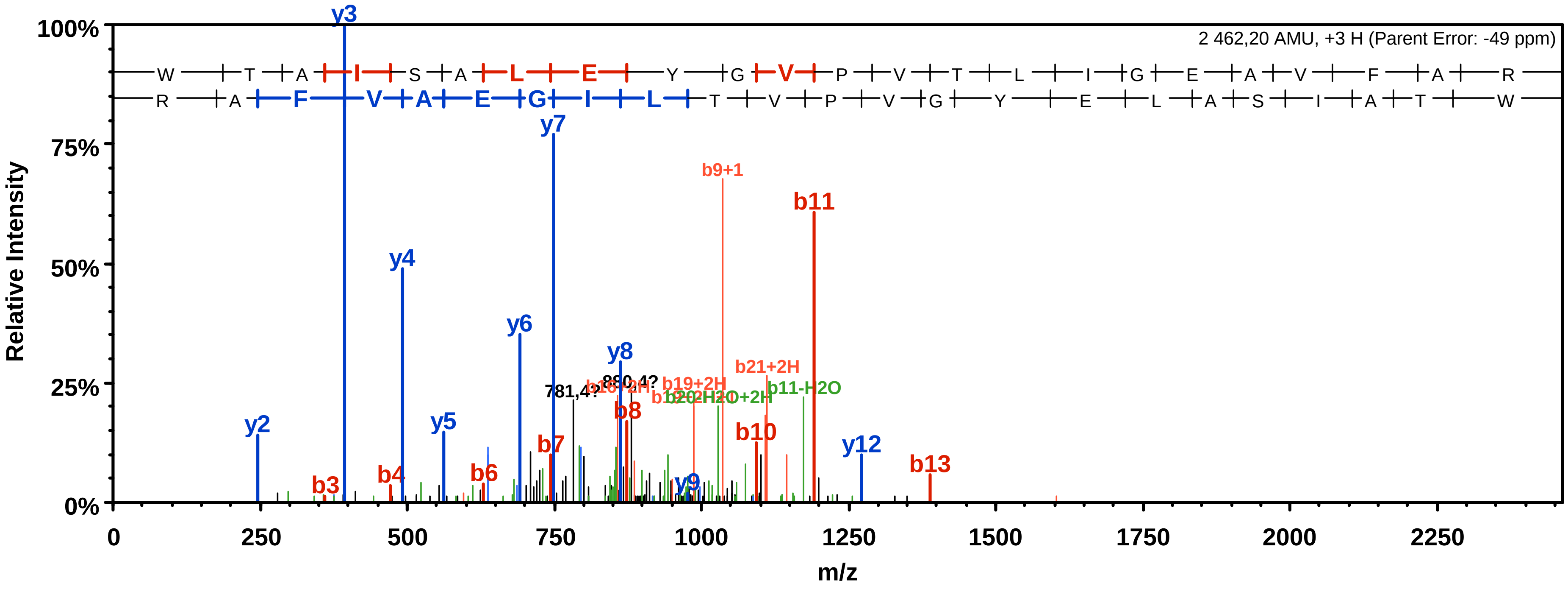

Biological sample name	MS/MS sample name	Protein name	Peptide sequence	Mascot Ion score
**Chromofungin_20**	F011551 (F013456)	6-phosphogluconate dehydrogenase, decarboxylating - Homo sapiens (Human)	WTAISALEYGVPVTLIGEAVFAR	67,7

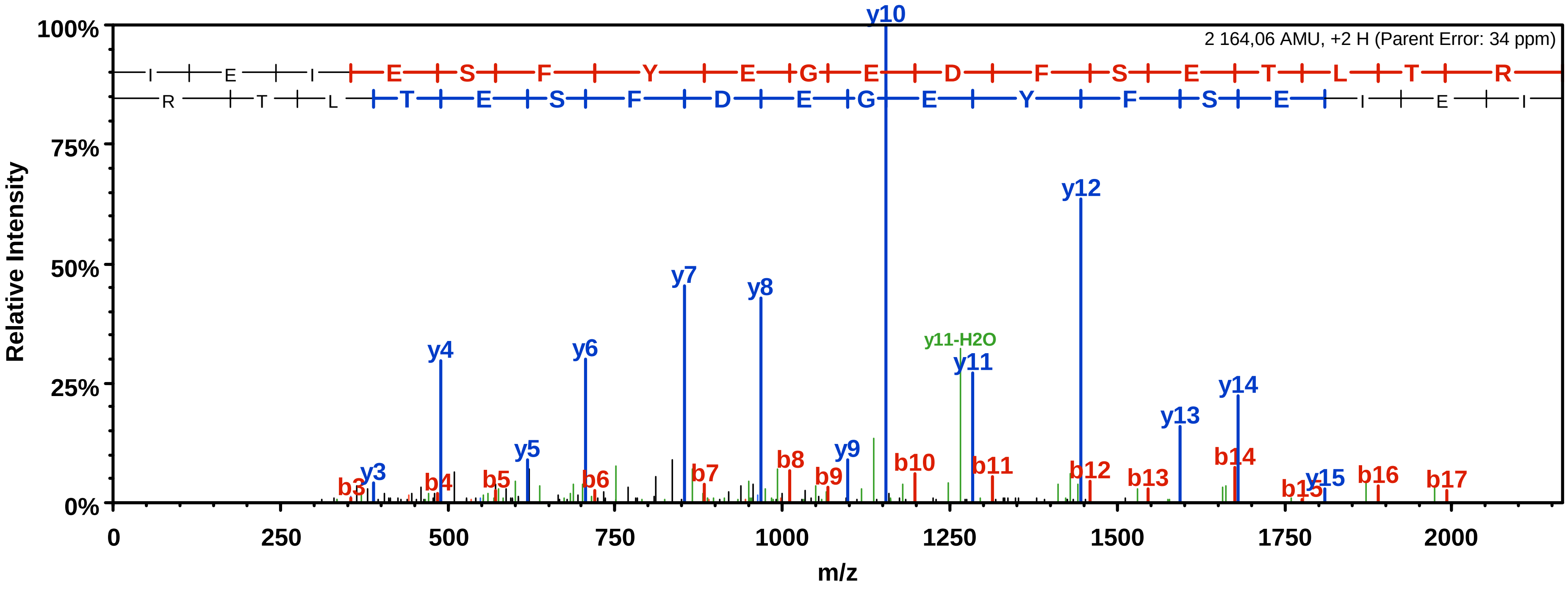

Biological sample name	MS/MS sample name	Protein name	Peptide sequence	Mascot Ion score
**Chromofungin_15-16**	F011547 (F013448)	78 kDa glucose-regulated protein precursor - Homo sapiens (Human)	IEIESFYEGEDFSETLTR	110

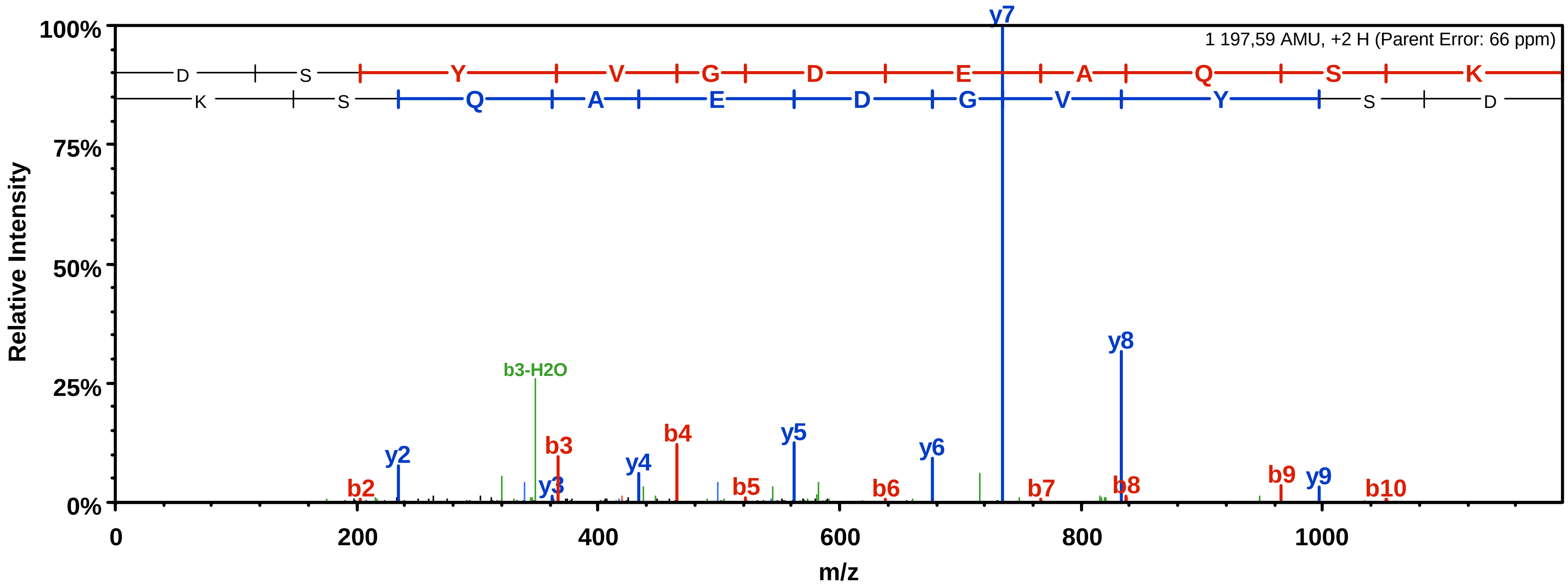

Biological sample name	MS/MS sample name	Protein name	Peptide sequence	Mascot Ion score
**Chromofungin_10**	F011542 (F013438)	Actin, cytoplasmic 1 - Homo sapiens (Human)	DSYVGDEAQSK	72,4

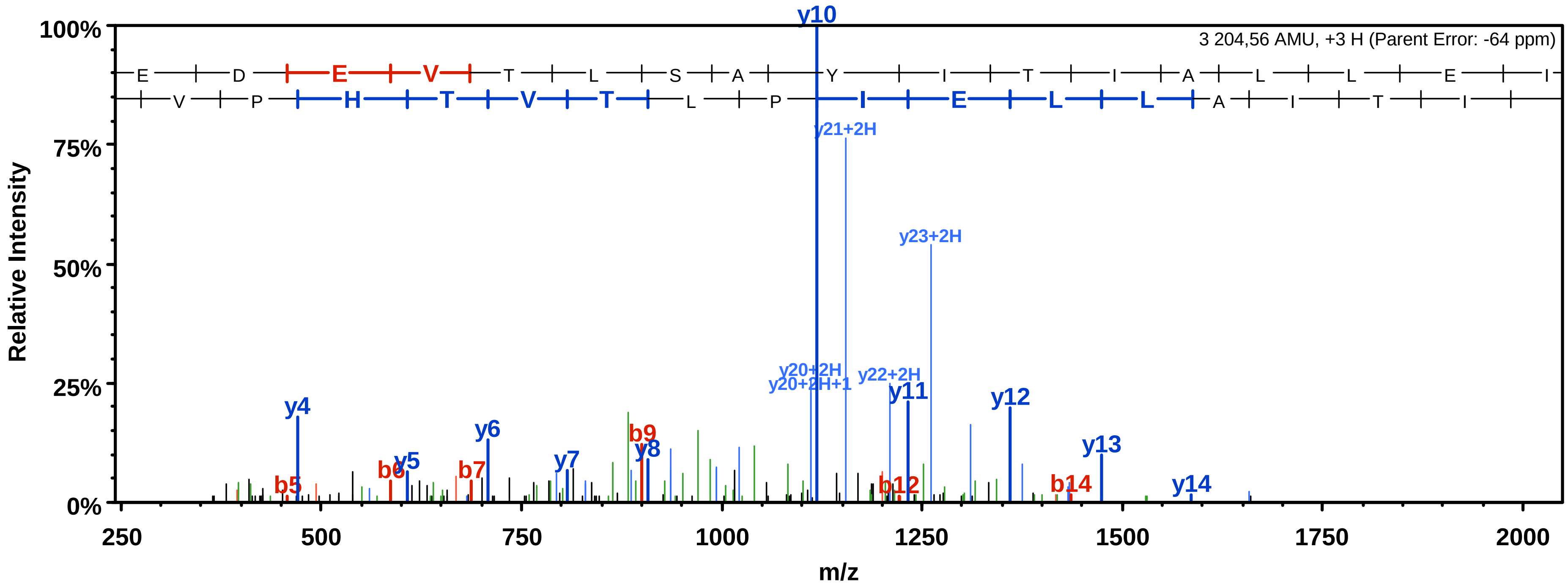

Biological sample name	MS/MS sample name	Protein name	Peptide sequence	Mascot Ion score
**Chromofungin_20**	F011551 (F013456)	Alpha-2-macroglobulin precursor - Homo sapiens (Human)	GGVEDEVTLSAYITIALLEIPLTVTHPVVR	91,3

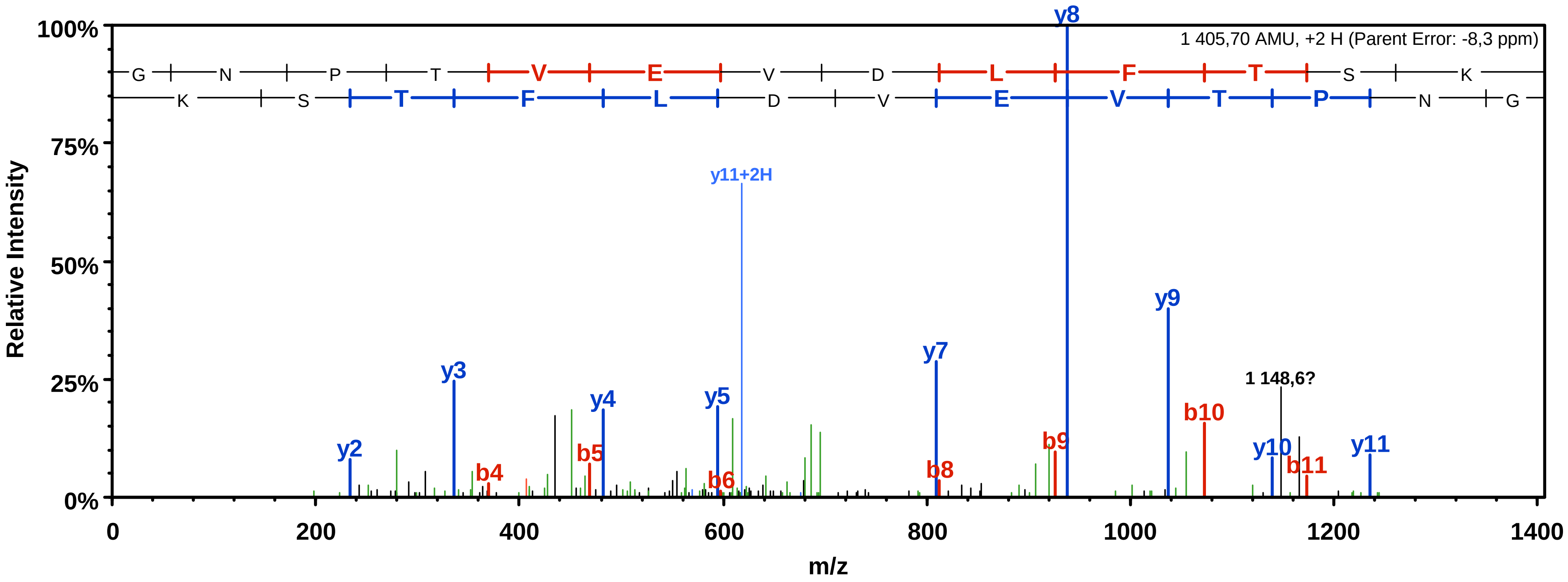

Biological sample name	MS/MS sample name	Protein name	Peptide sequence	Mascot Ion score
**Chromofungin_14**	F011546 (F013446)	Alpha-enolase - Homo sapiens (Human)	GNPTVEVDLFTSK	62,3

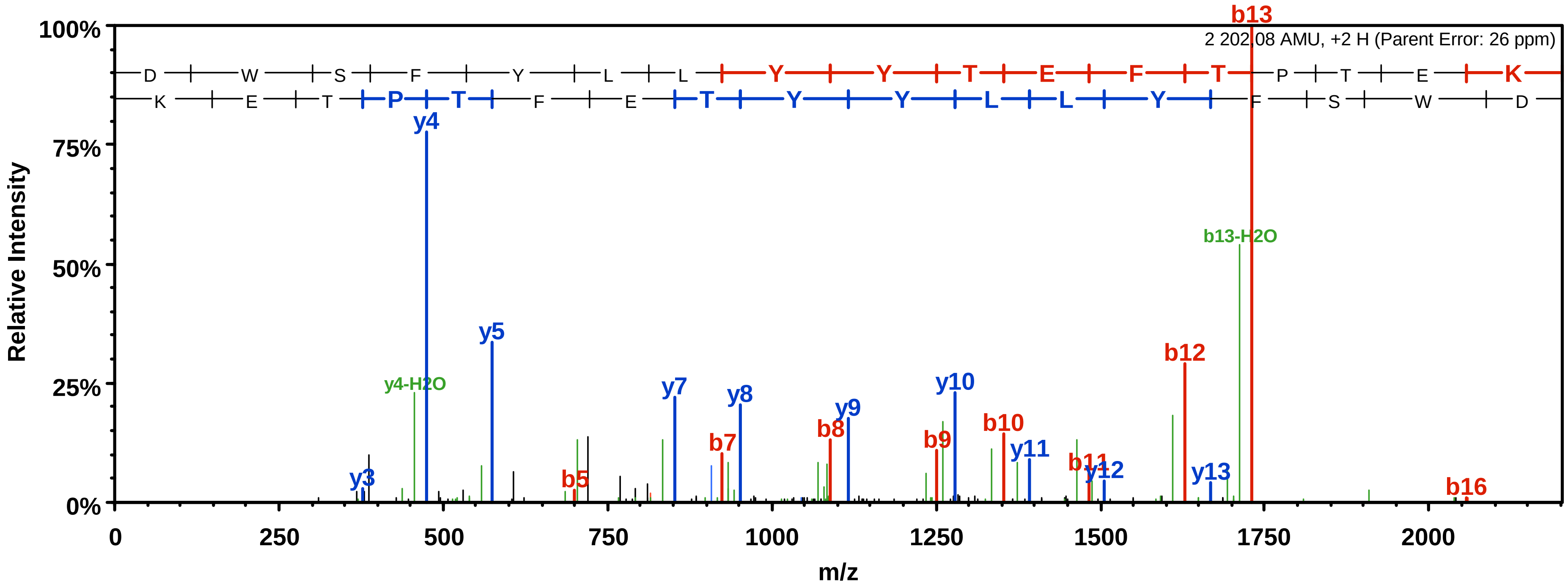

Biological sample name	MS/MS sample name	Protein name	Peptide sequence	Mascot Ion score
**Chromofungin_13**	F011545 (F013444)	Beta-2-microglobulin precursor [Contains: Beta-2-microglobulin form pI 5.3] - Homo sapiens (Human)	DWSFYLLYYTEFTPTEK	53,7

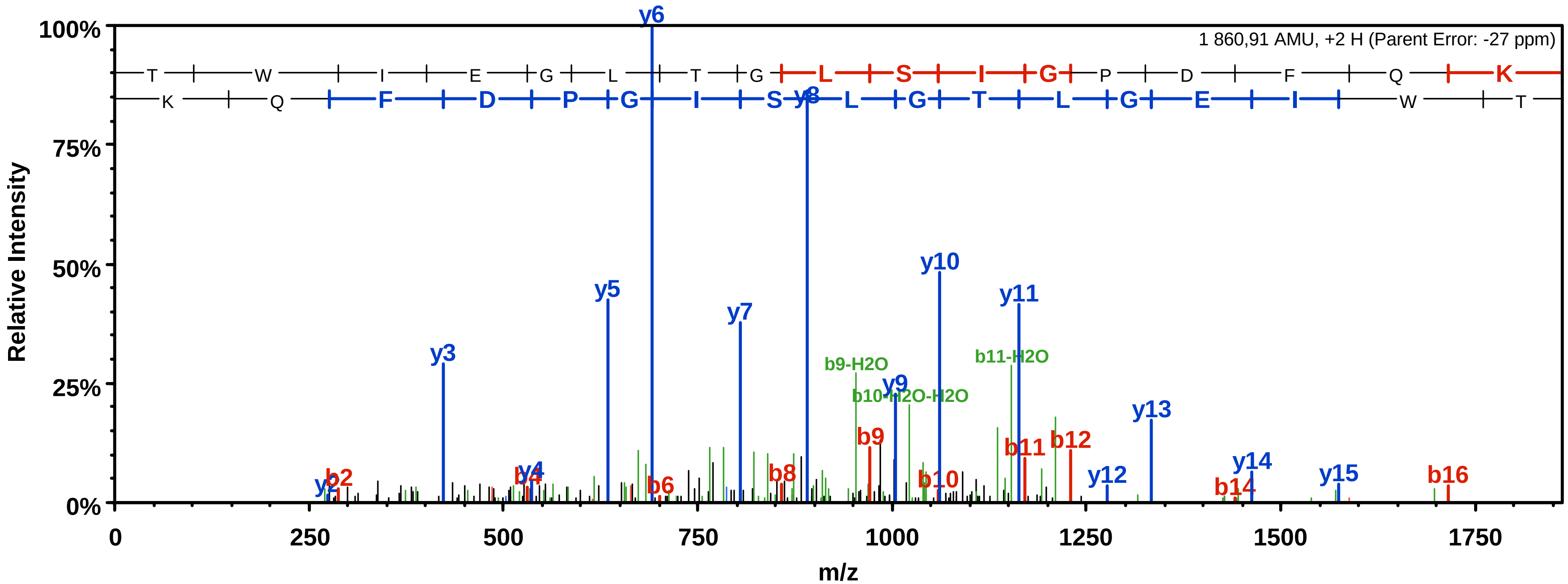

Biological sample name	MS/MS sample name	Protein name	Peptide sequence	Mascot Ion score
**Chromofungin_18**	F011549 (F013452)	Calponin-2 - Homo sapiens (Human)	TWIEGLTGLSIGPDFQK	118

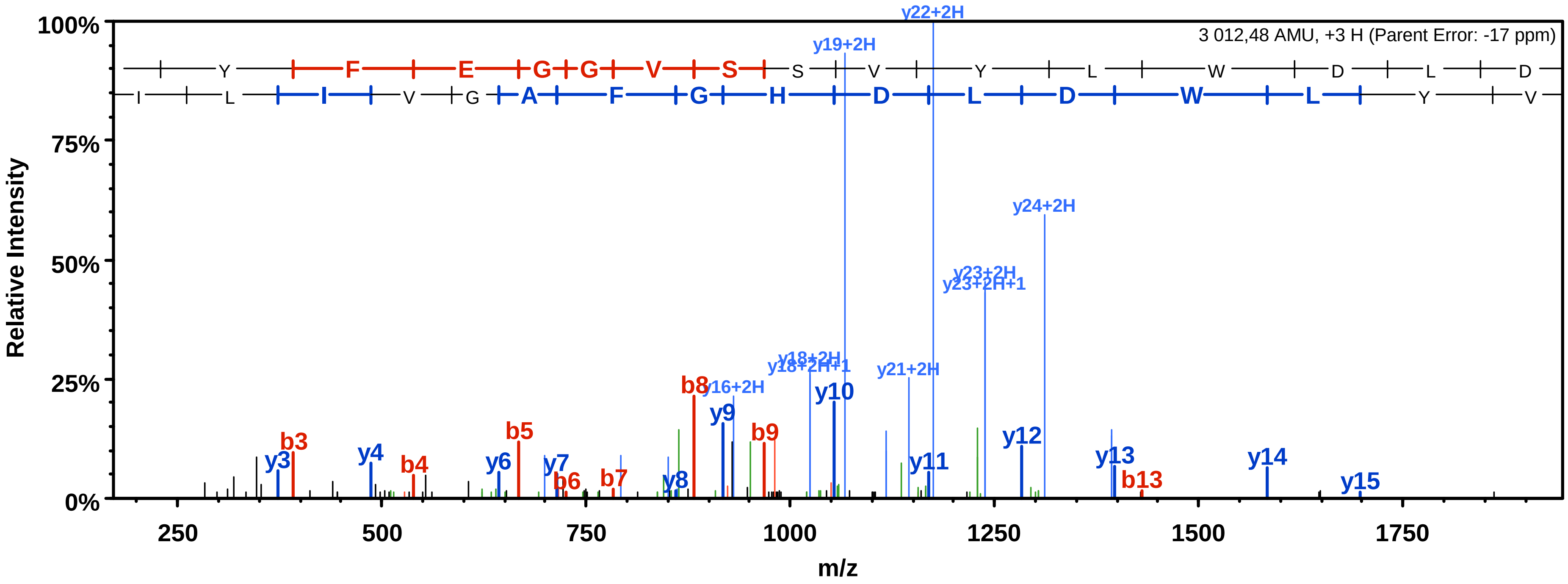

Biological sample name	MS/MS sample name	Protein name	Peptide sequence	Mascot Ion score
**Chromofungin_15-16**	F011547 (F013448)	F-actin-capping protein subunit beta - Homo sapiens (Human)	DLYFEGGVSSVYLWDLDHGFAGVILIK	85,7

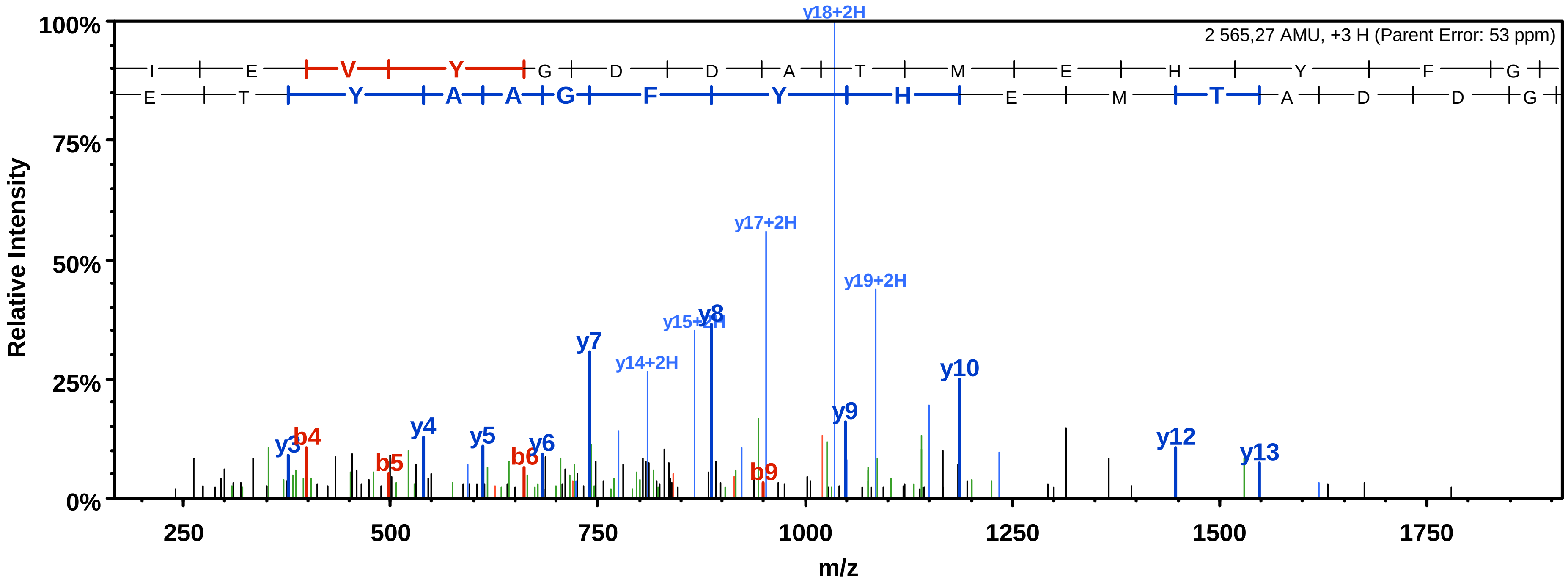

Biological sample name	MS/MS sample name	Protein name	Peptide sequence	Mascot Ion score
**Chromofungin_6**	F011538 (F013430)	Genome polyprotein [Contains: P1 proteinase - Lettuce mosaic virus (strain 0 / isolate French) (LMV)	VGIEVYGDDATMEHYFGAAYTEK	75,8

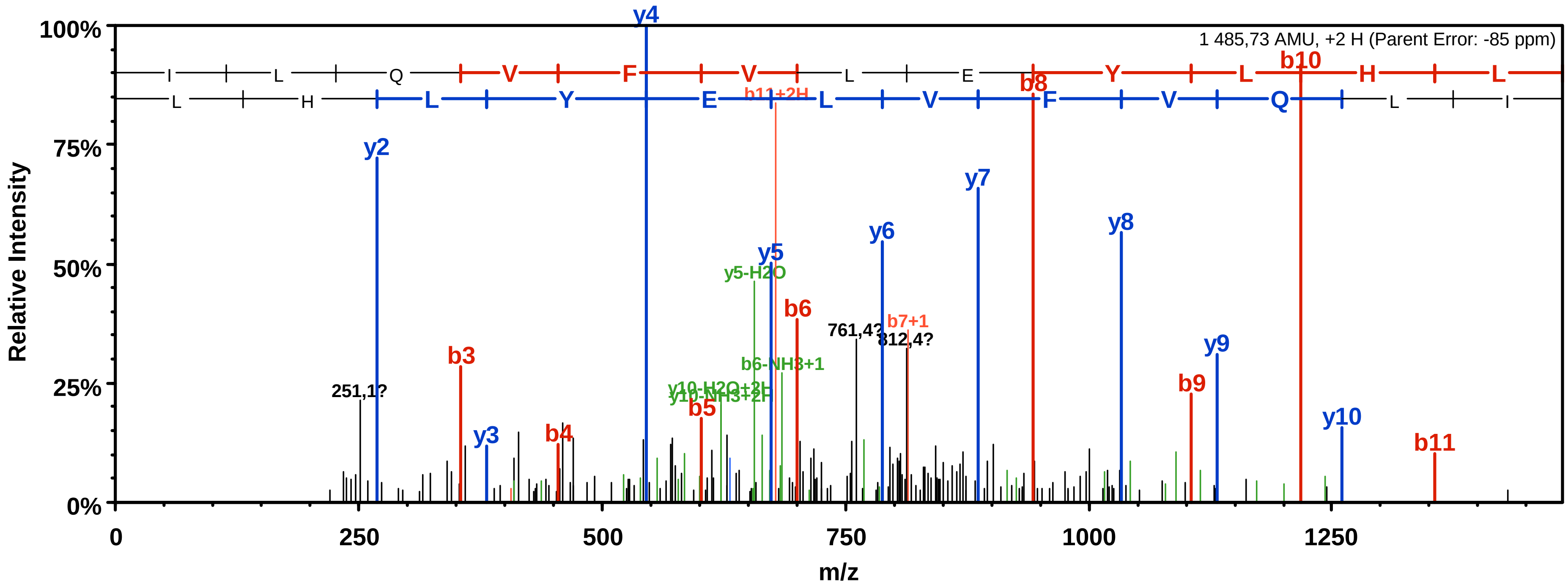

Biological sample name	MS/MS sample name	Protein name	Peptide sequence	Mascot Ion score
**Chromofungin_19**	F011550 (F013454)	Glutaminyl-peptide cyclotransferase precursor - Homo sapiens (Human)	ILQVFVLEYLHL	60,8

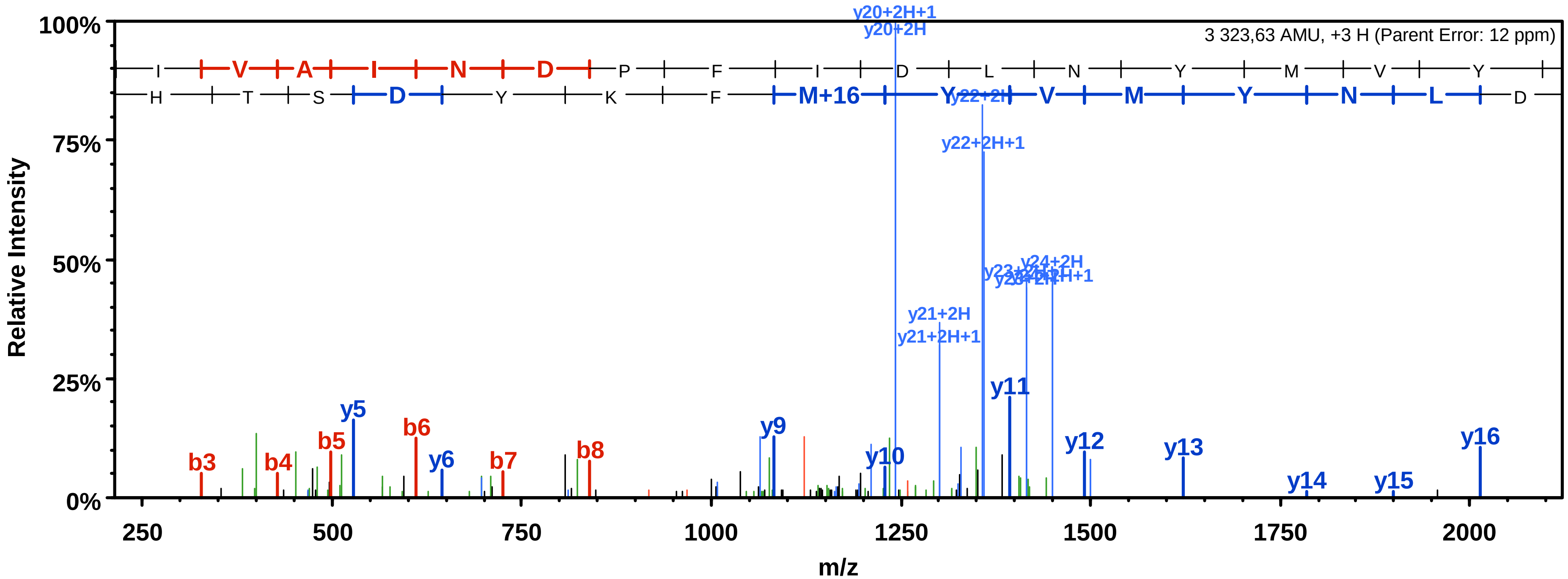

Biological sample name	MS/MS sample name	Protein name	Peptide sequence	Mascot Ion score
**Chromofungin_15-16**	F011547 (F013448)	Glyceraldehyde-3-phosphate dehydrogenase, muscle - Jaculus orientalis (Greater Egyptian jerboa)	VDIVAINDPFIDLNYMVYMFKYDSTHGK	66,2

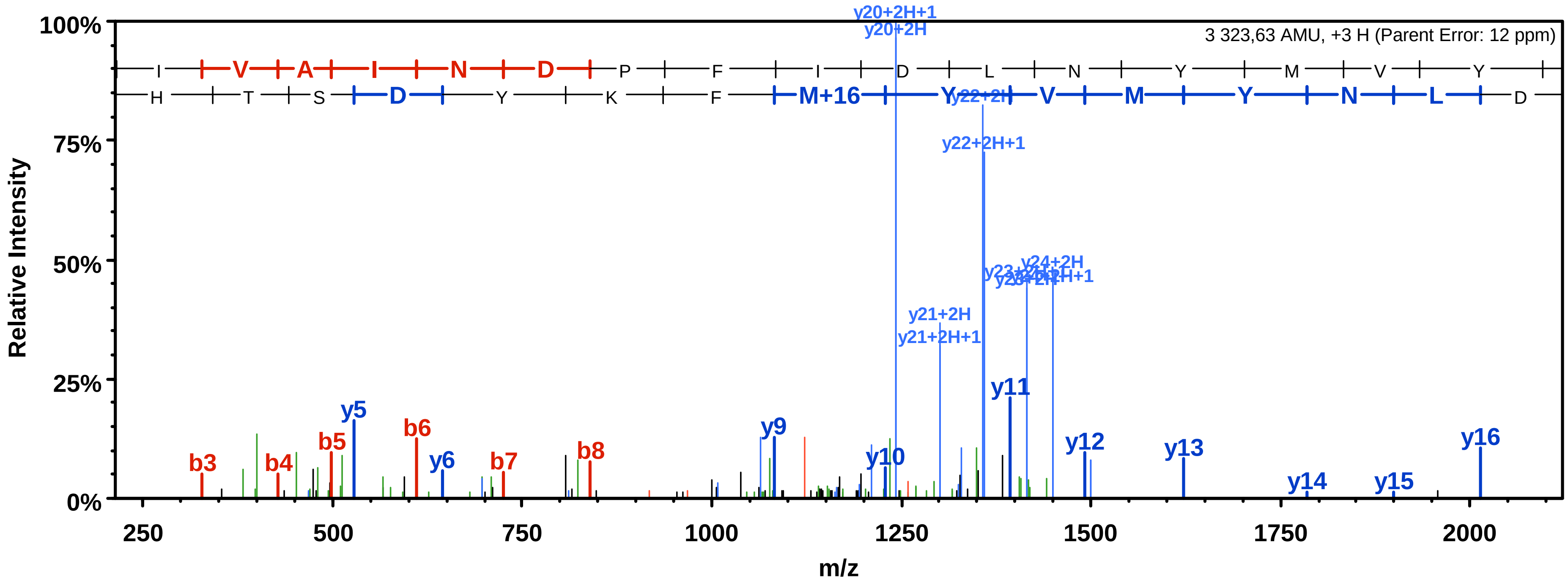

Biological sample name	MS/MS sample name	Protein name	Peptide sequence	Mascot Ion score
**Chromofungin_15-16**	F011547 (F013448)	Heat shock protein HSP 90-alpha - Homo sapiens (Human)	DLVILLYETALLSSGFSLEDPQTHAN R	120

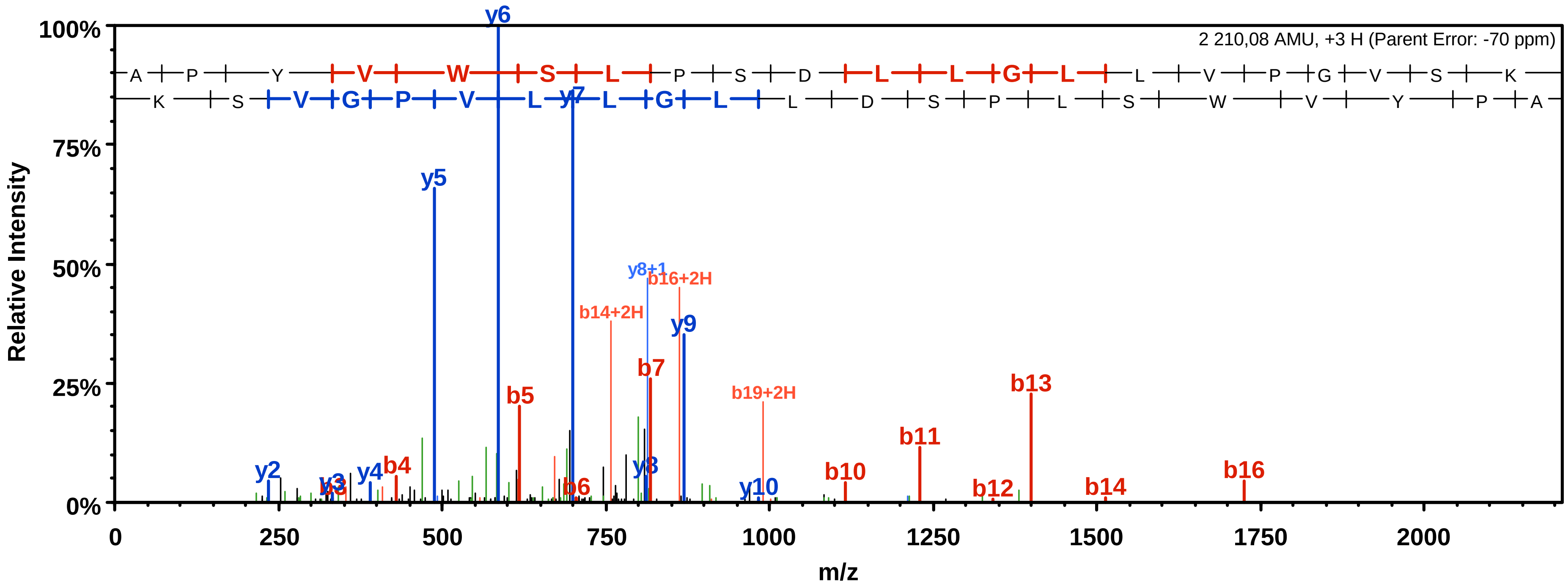

Biological sample name	MS/MS sample name	Protein name	Peptide sequence	Mascot Ion score
**Chromofungin_13**	F011545 (F013444)	Leukocyte immunoglobulin-like receptor subfamily A member 3 precursor - Homo sapiens (Human)	APYVWSLPSDLLGLLVPGVSK	54,1

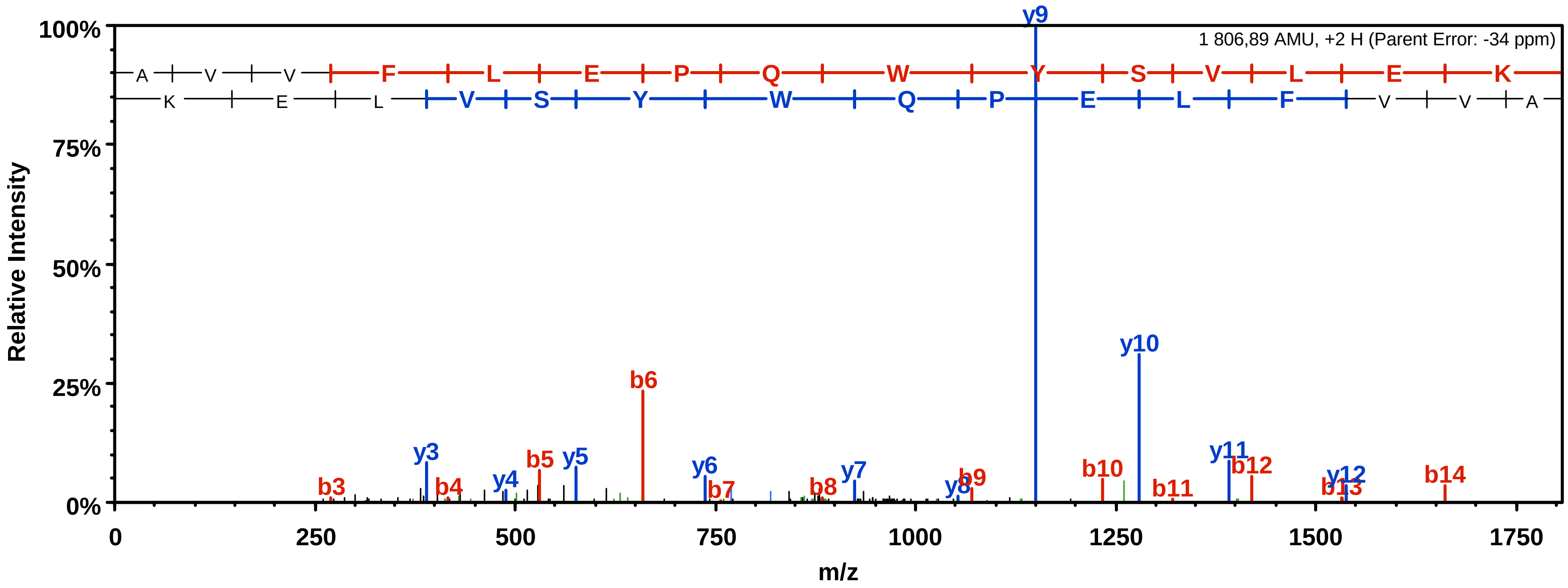

Biological sample name	MS/MS sample name	Protein name	Peptide sequence	Mascot Ion score
**Chromofungin_13**	F011545 (F013444)	Low affinity immunoglobulin gamma Fc region receptor III-B precursor - Homo sapiens (Human)	AVVFLEPQWYSVLEK	87,7

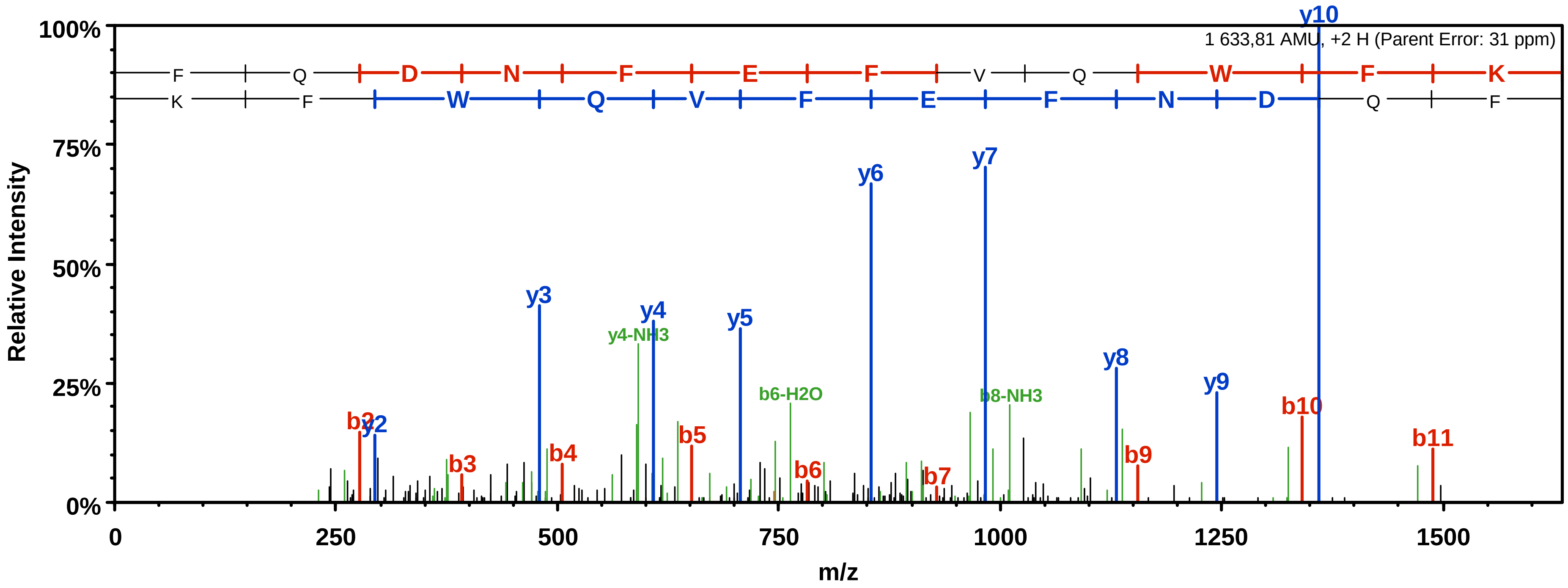

Biological sample name	MS/MS sample name	Protein name	Peptide sequence	Mascot Ion score
**Chromofungin_19**	F011550 (F013454)	Microtubule-associated protein RP/EB family member 1 - Homo sapiens (Human)	FQDNFEFVQWFK	82,7

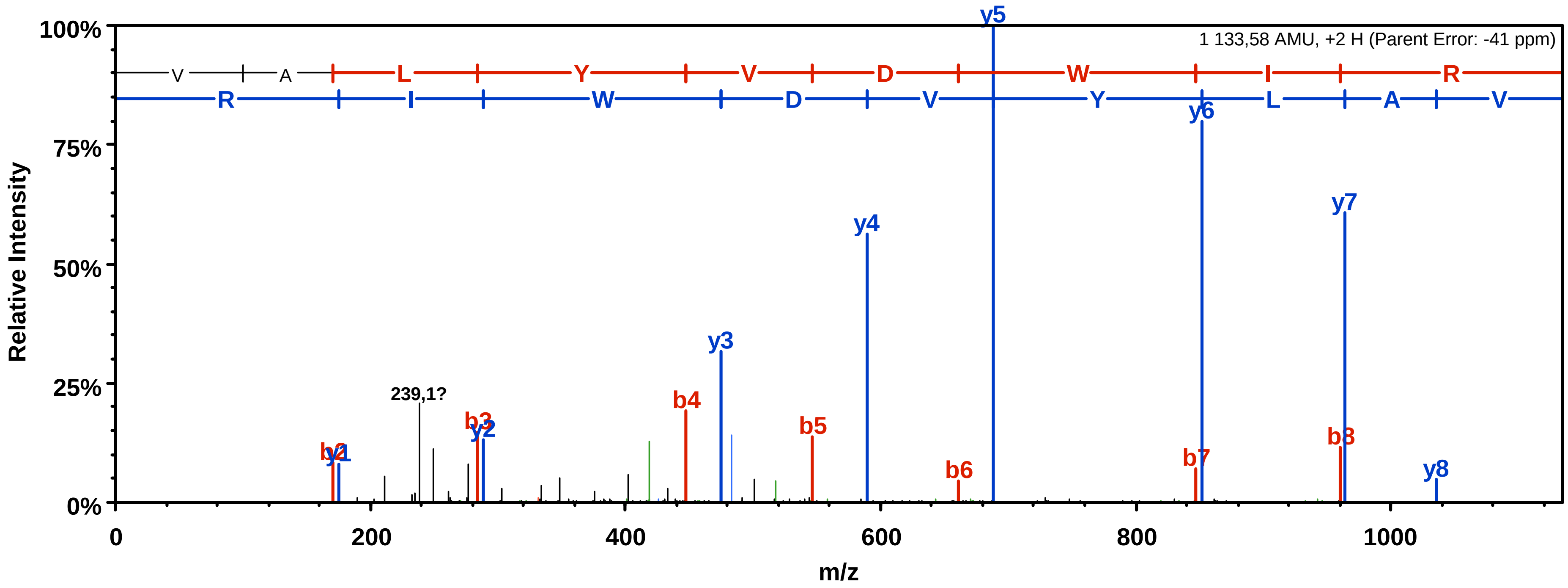

Biological sample name	MS/MS sample name	Protein name	Peptide sequence	Mascot Ion score
**Chromofungin_17**	F011548 (F013450)	Myeloblastin precursor - Homo sapiens (Human)	VALYVDWIR	58,1

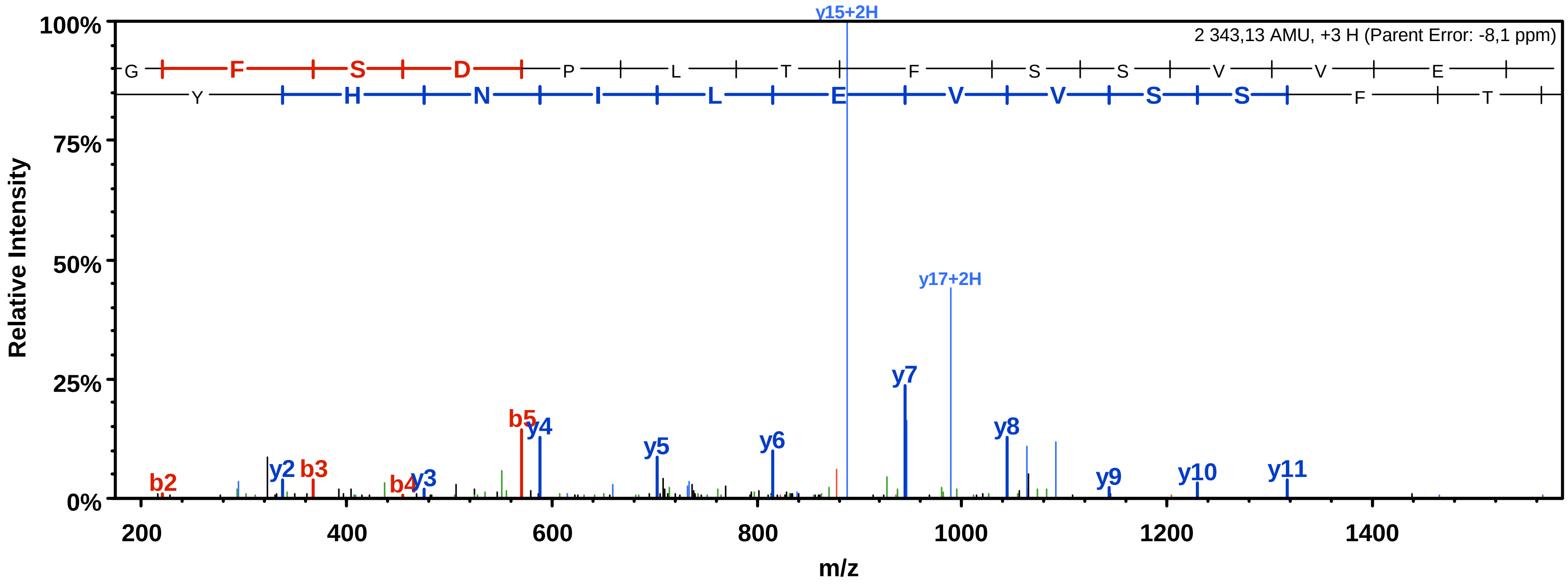

Biological sample name	MS/MS sample name	Protein name	Peptide sequence	Mascot Ion score
**Chromofungin_14**	F011546 (F013446)	Phosphatidylinositol 3-kinase regulatory subunit alpha - Homo sapiens (Human)	YGFSDPLTFSSVVELINHYR	58,1

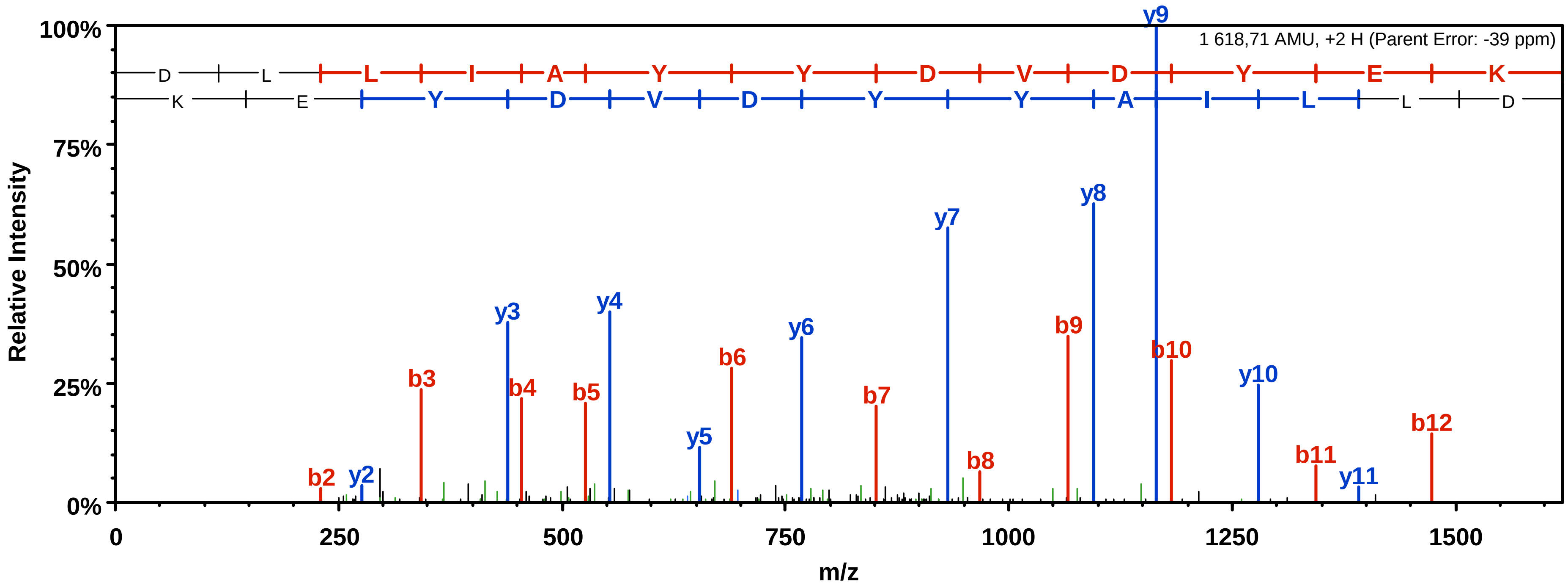

Biological sample name	MS/MS sample name	Protein name	Peptide sequence	Mascot Ion score
**Chromofungin_15-16**	F011547 (F013448)	Protein disulfide-isomerase A3 precursor - Homo sapiens (Human)	DLLIAYYDVDYEK	96,5

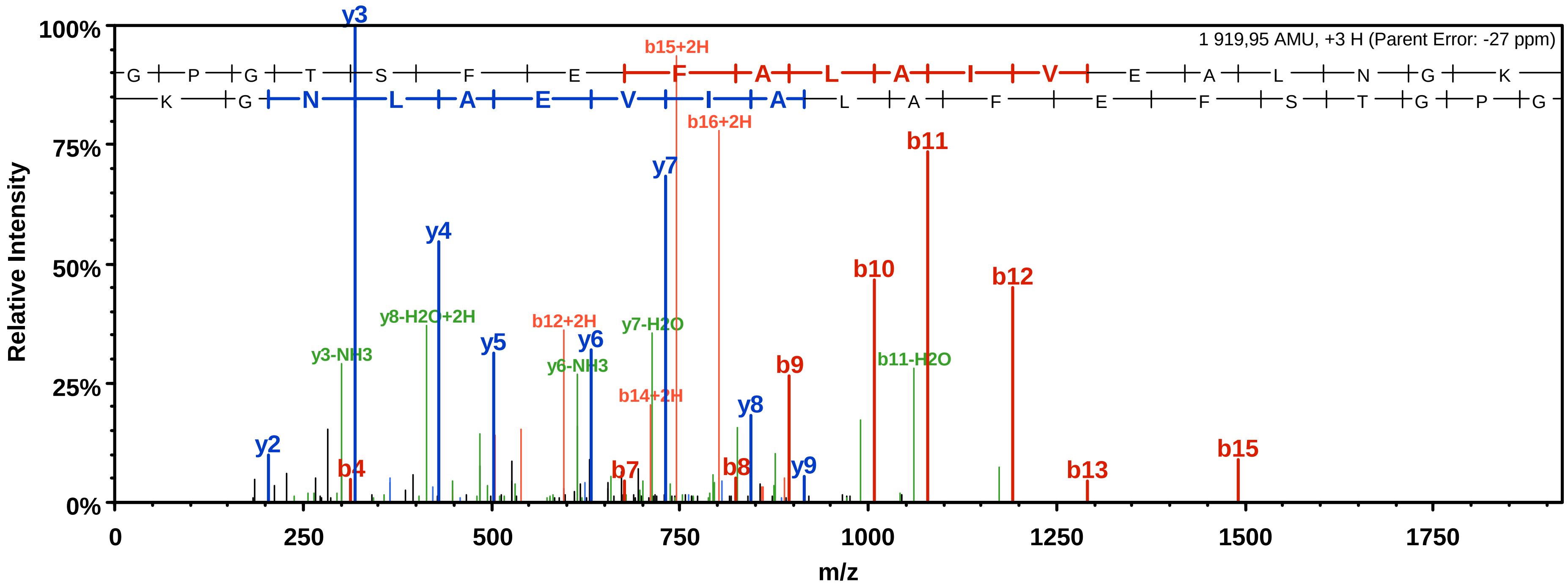

Biological sample name	MS/MS sample name	Protein name	Peptide sequence	Mascot Ion score
**Chromofungin_15-16**	F011547 (F013448)	Protein DJ-1 - Homo sapiens (Human)	GPGTSFEFALAIVEALNGK	75,2

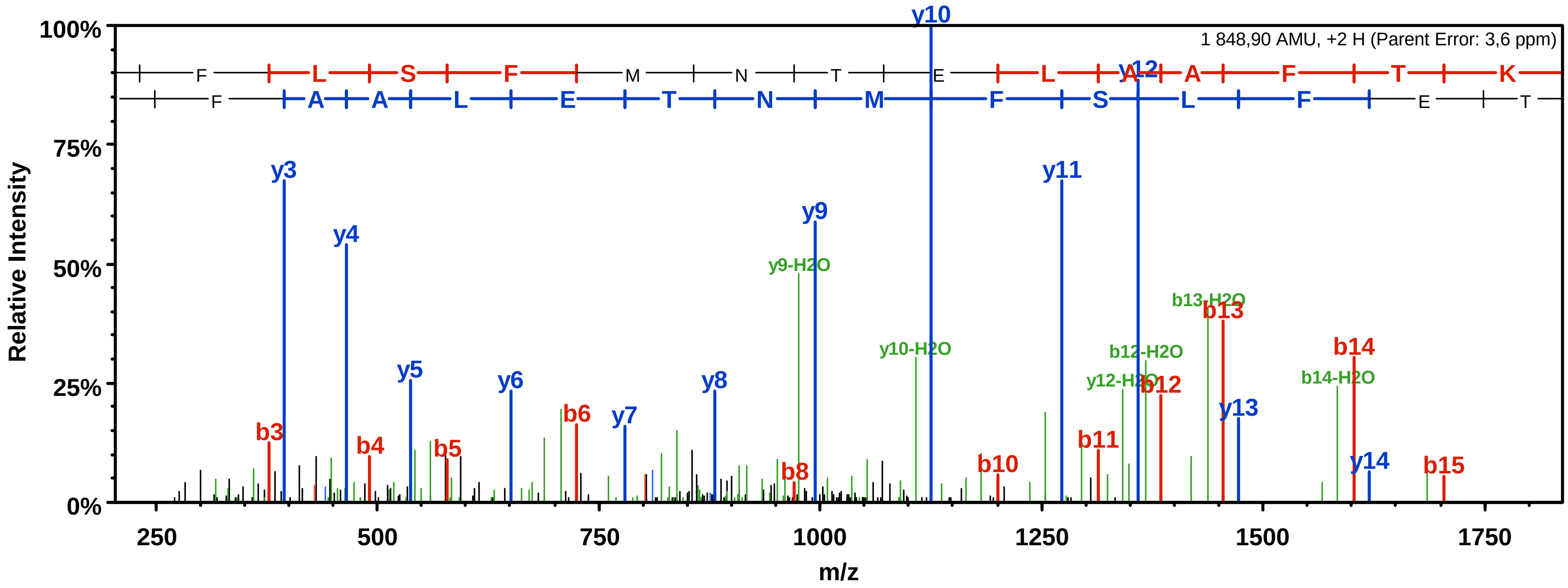

Biological sample name	MS/MS sample name	Protein name	Peptide sequence	Mascot Ion score
**Chromofungin_17**	F011548 (F013450)	Protein S100-A11 - Homo sapiens (Human)	TEFLSFMNTELAAFTK	106

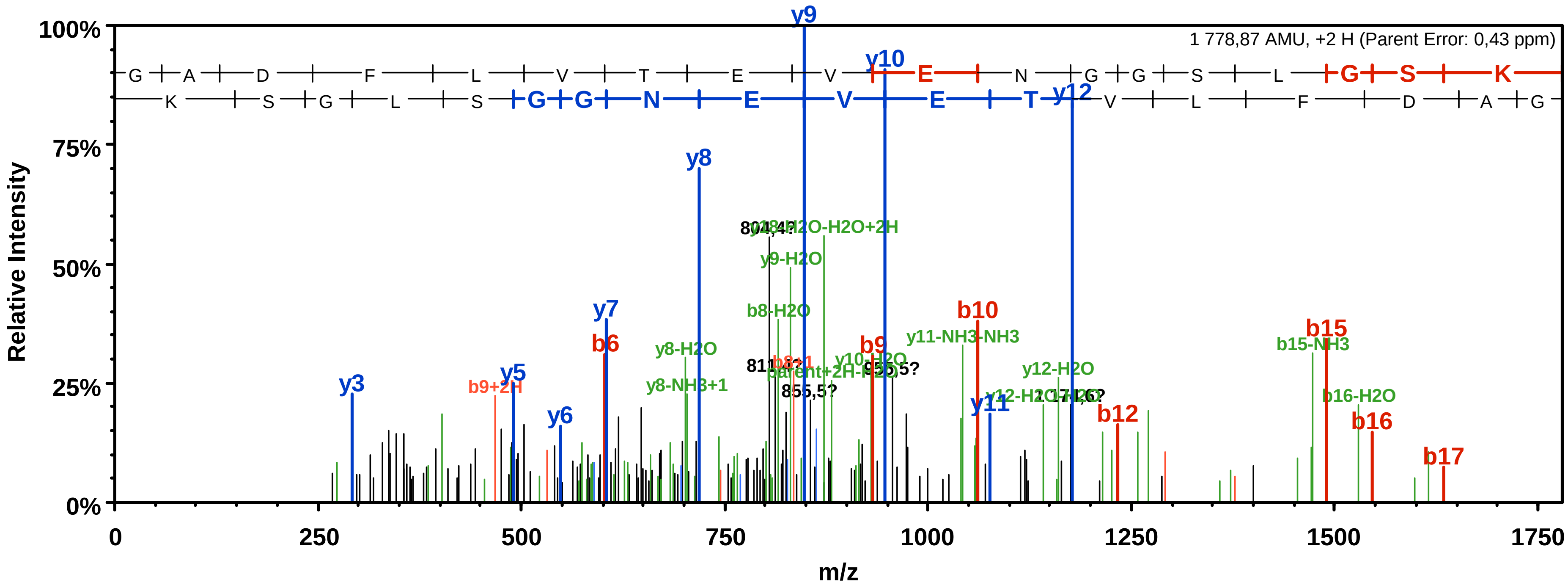

Biological sample name	MS/MS sample name	Protein name	Peptide sequence	Mascot Ion score
**Chromofungin_10**	F011542 (F013438)	Pyruvate kinase isozymes M1/M2 - Homo sapiens (Human)	GADFLVTEVENGGSLGSK	54

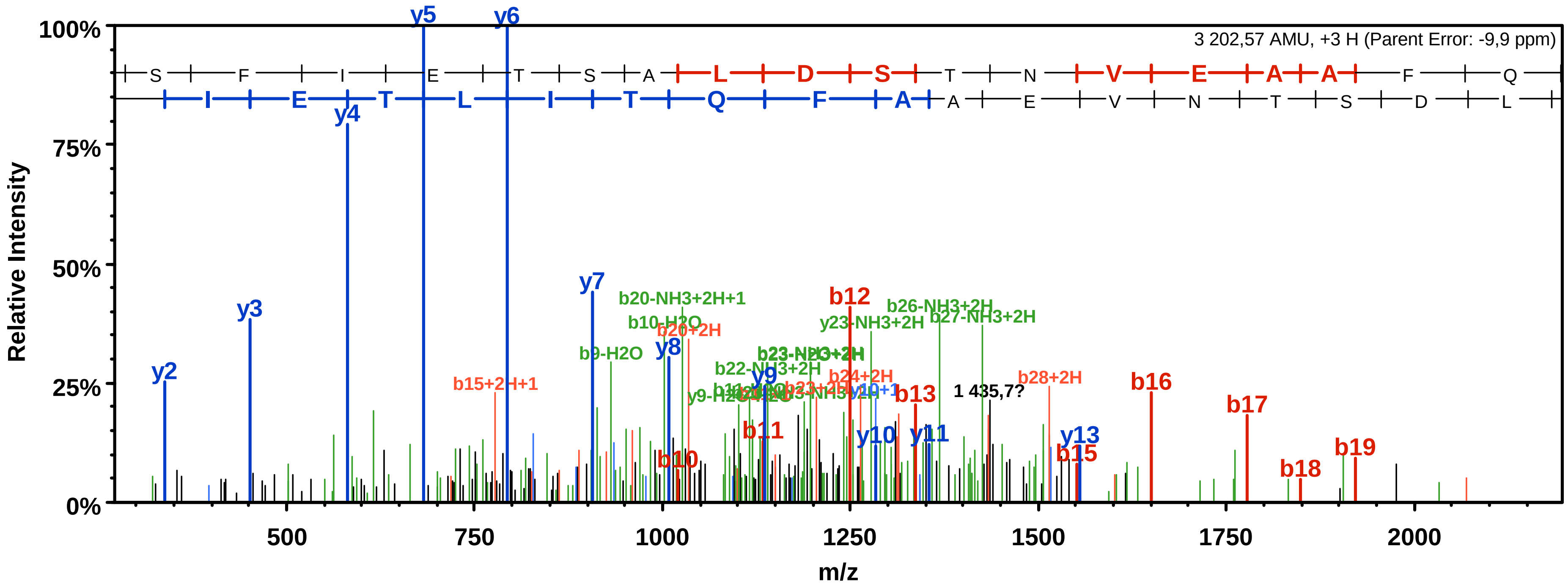

Biological sample name	MS/MS sample name	Protein name	Peptide sequence	Mascot Ion score
**Chromofungin_19**	F011550 (F013454)	Ras-related protein Rab-11A - Homo sapiens (Human)	NGLSFIETSALDSTNVEAAFQTILTEI YR	54,8

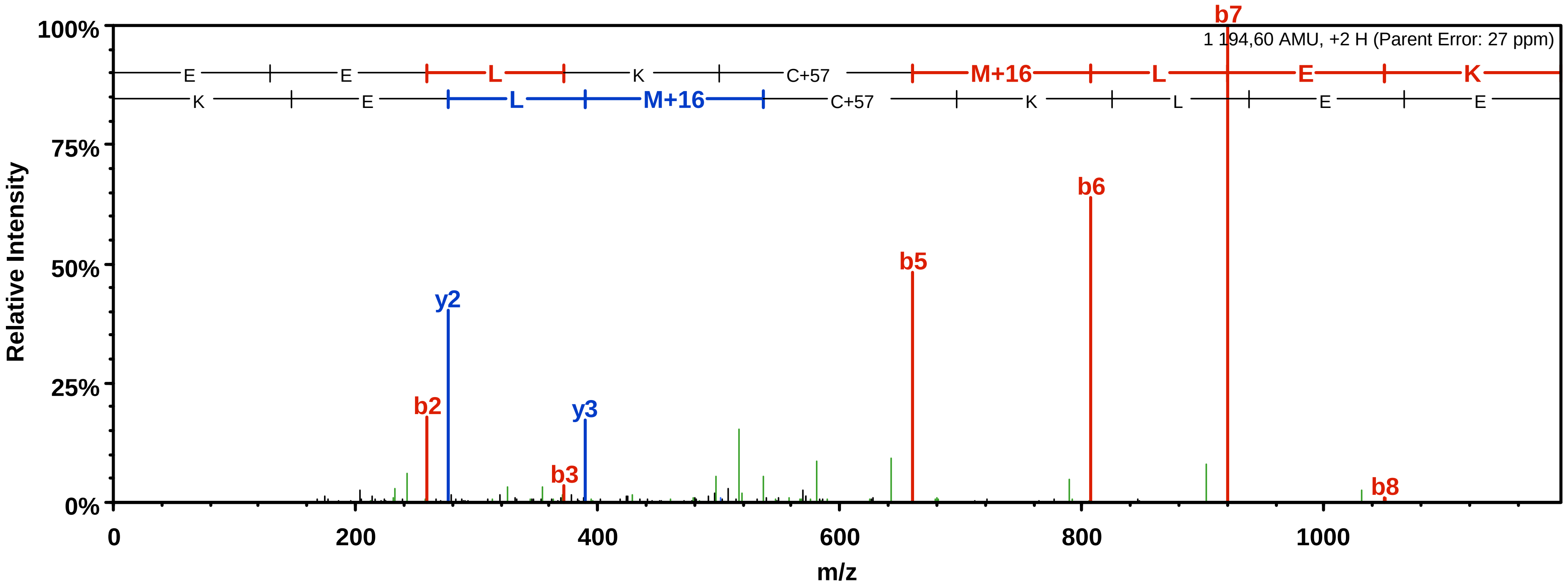

Biological sample name	MS/MS sample name	Protein name	Peptide sequence	Mascot Ion score
**Chromofungin_14**	F011546 (F013446)	Testis-specific gene 10 protein - Homo sapiens (Human)	EELKCMLEK	55,6

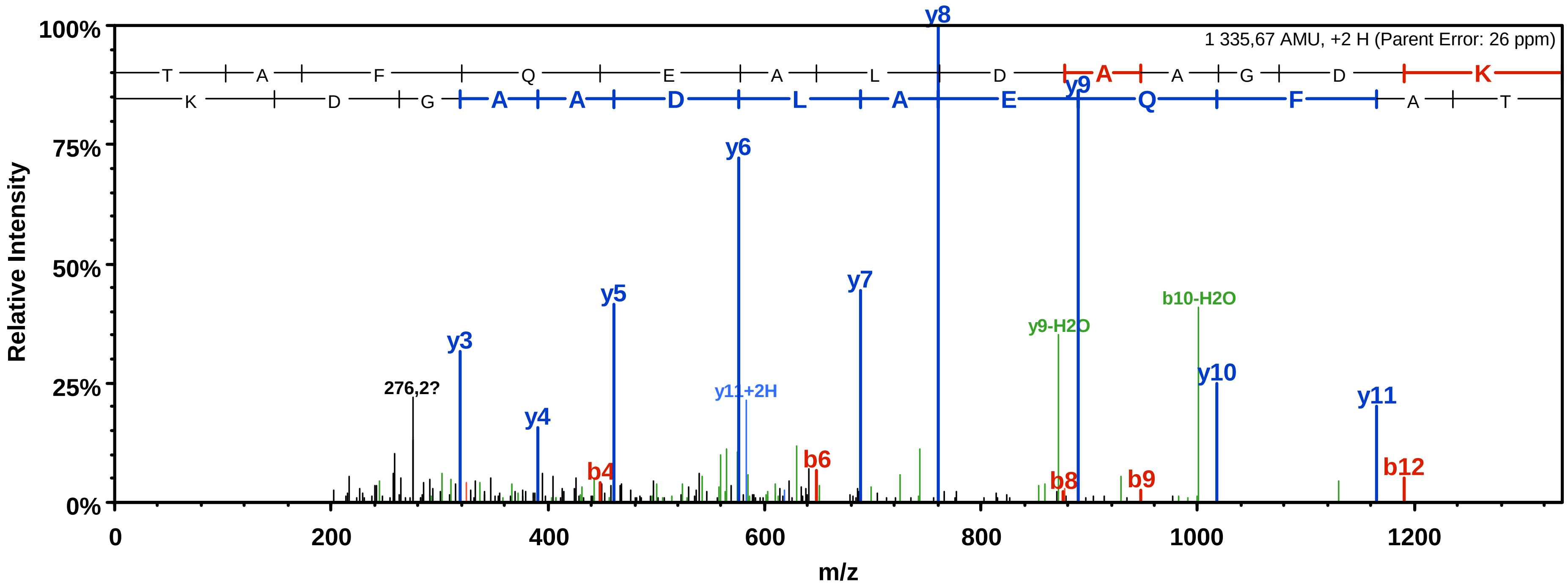

Biological sample name	MS/MS sample name	Protein name	Peptide sequence	Mascot Ion score
**Chromofungin_13**	F011545 (F013444)	Thioredoxin - Homo sapiens (Human)	TAFQEALDAAGDK	79,4

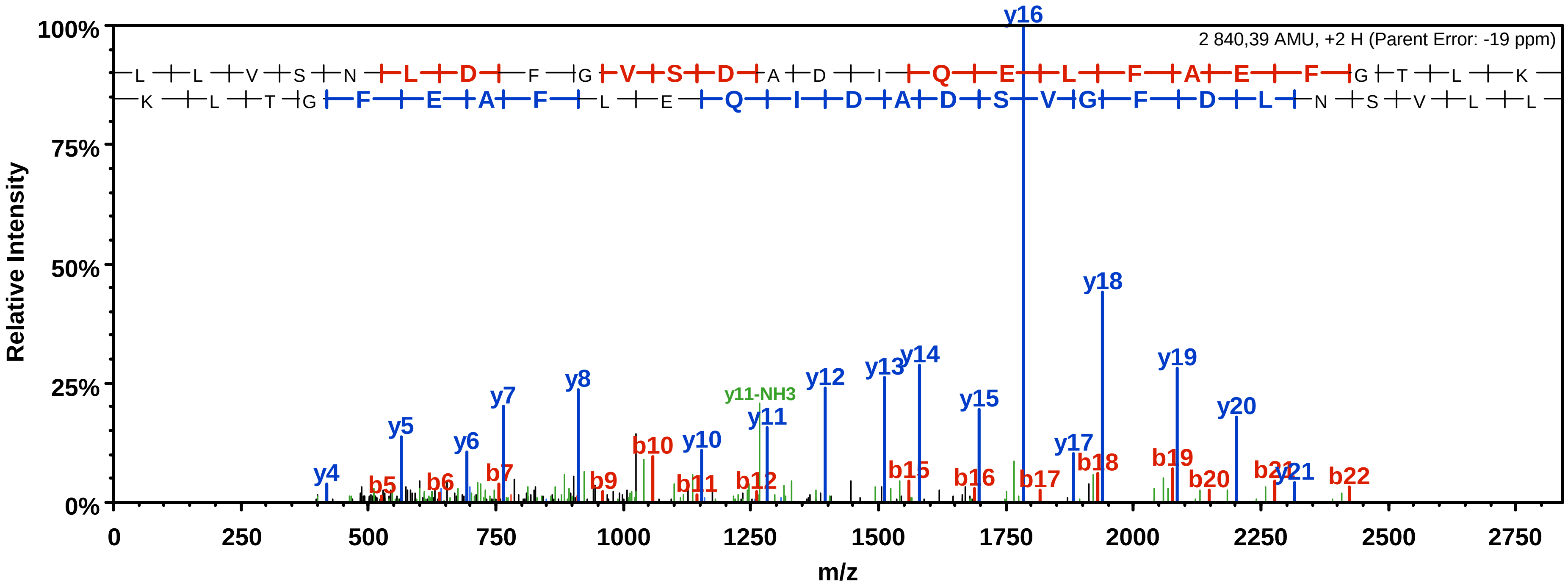

Biological sample name	MS/MS sample name	Protein name	Peptide sequence	Mascot Ion score
**Chromofungin_14**	F011546 (F013446)	THO complex subunit 4 - Homo sapiens (Human)	LLVSNLDFGVSDADIQELFAEFGTLK	143

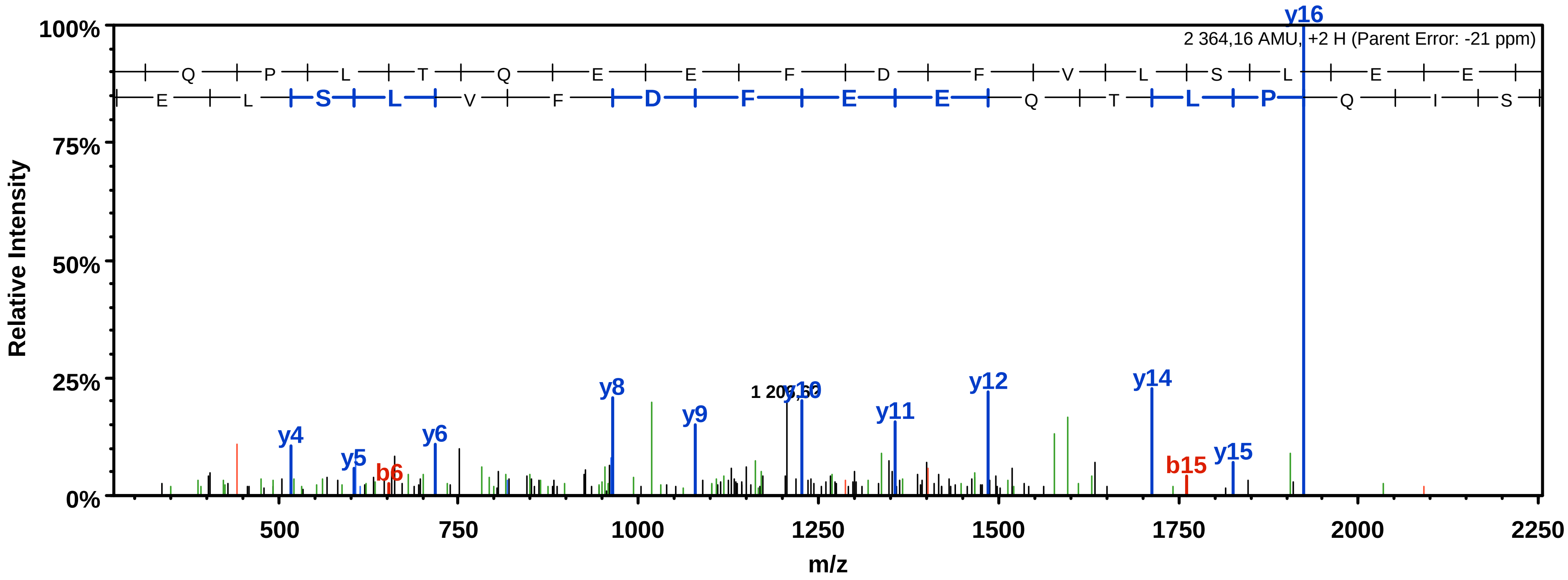

Biological sample name	MS/MS sample name	Protein name	Peptide sequence	Mascot Ion score
**Chromofungin_14**	F011546 (F013446)	Thymocyte nuclear protein 1 - Homo sapiens (Human)	LSIQPLTQEEFDFVLSLEEK	69

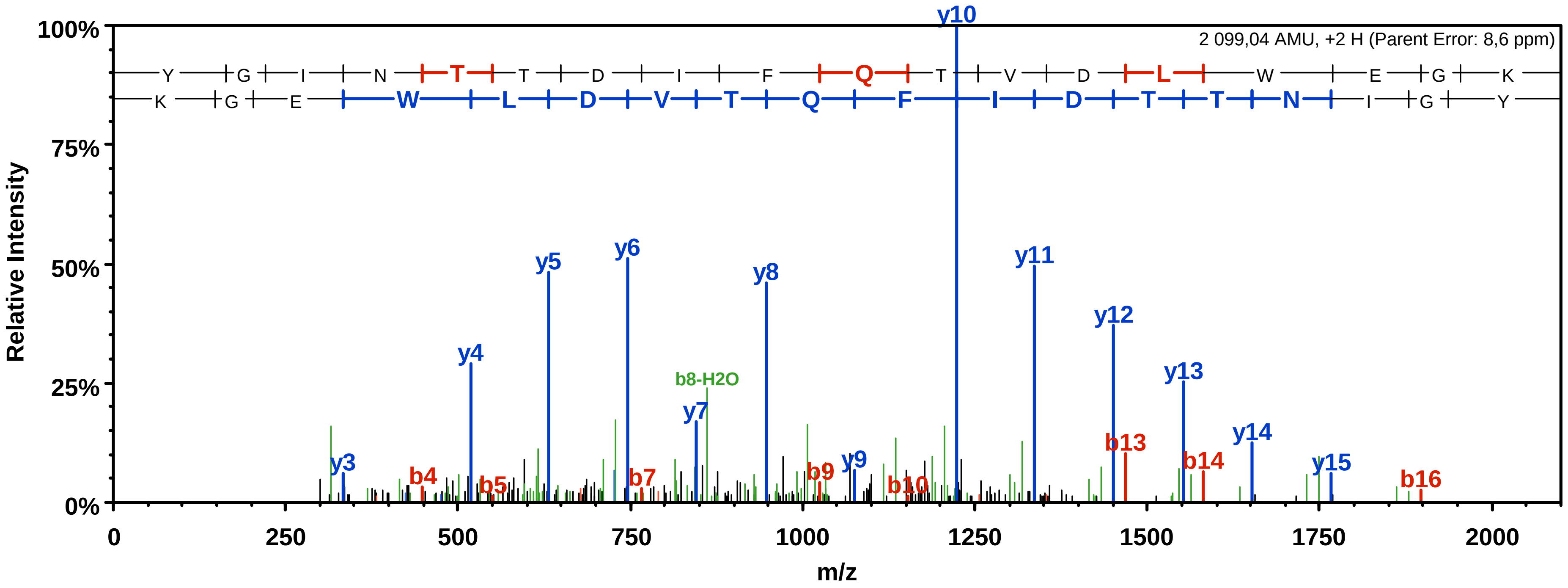

Biological sample name	MS/MS sample name	Protein name	Peptide sequence	Mascot Ion score
**Chromofungin_19**	F011550 (F013454)	Transgelin-2 - Homo sapiens (Human)	YGINTTDIFQTVDLWEGK	101

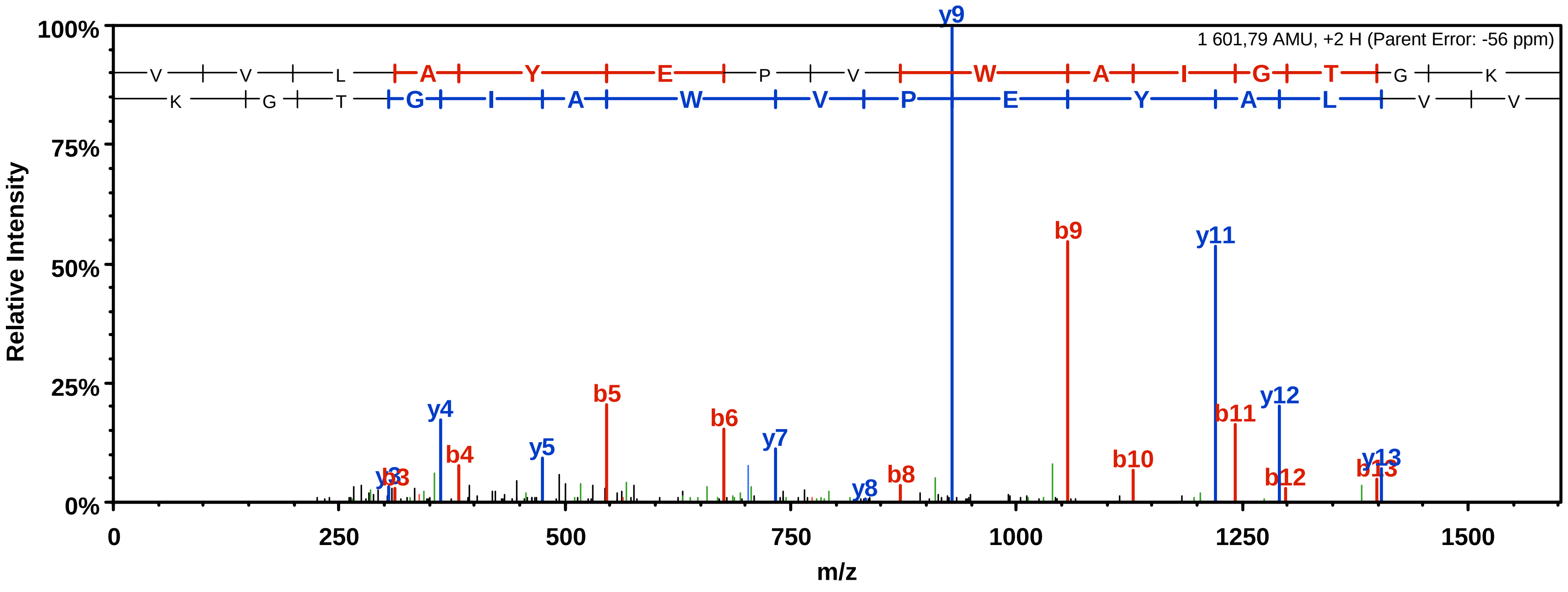

Biological sample name	MS/MS sample name	Protein name	Peptide sequence	Mascot Ion score
**Chromofungin_19**	F011550 (F013454)	Triosephosphate isomerase - Homo sapiens (Human)	VVLAYEPVWAIGTGK	89,9

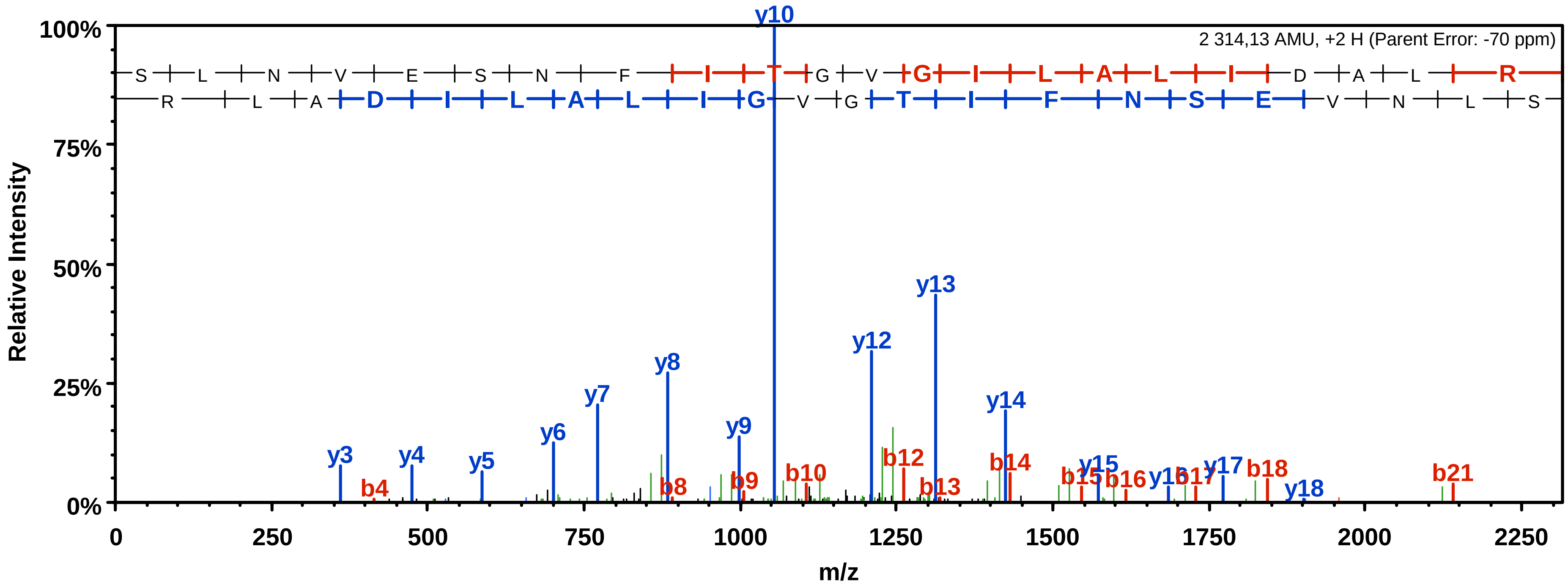

Biological sample name	MS/MS sample name	Protein name	Peptide sequence	Mascot Ion score
**Chromofungin_19**	F011550 (F013454)	Tropomodulin-3 - Homo sapiens (Human)	SLNVESNFITGVGILALIDALR	122

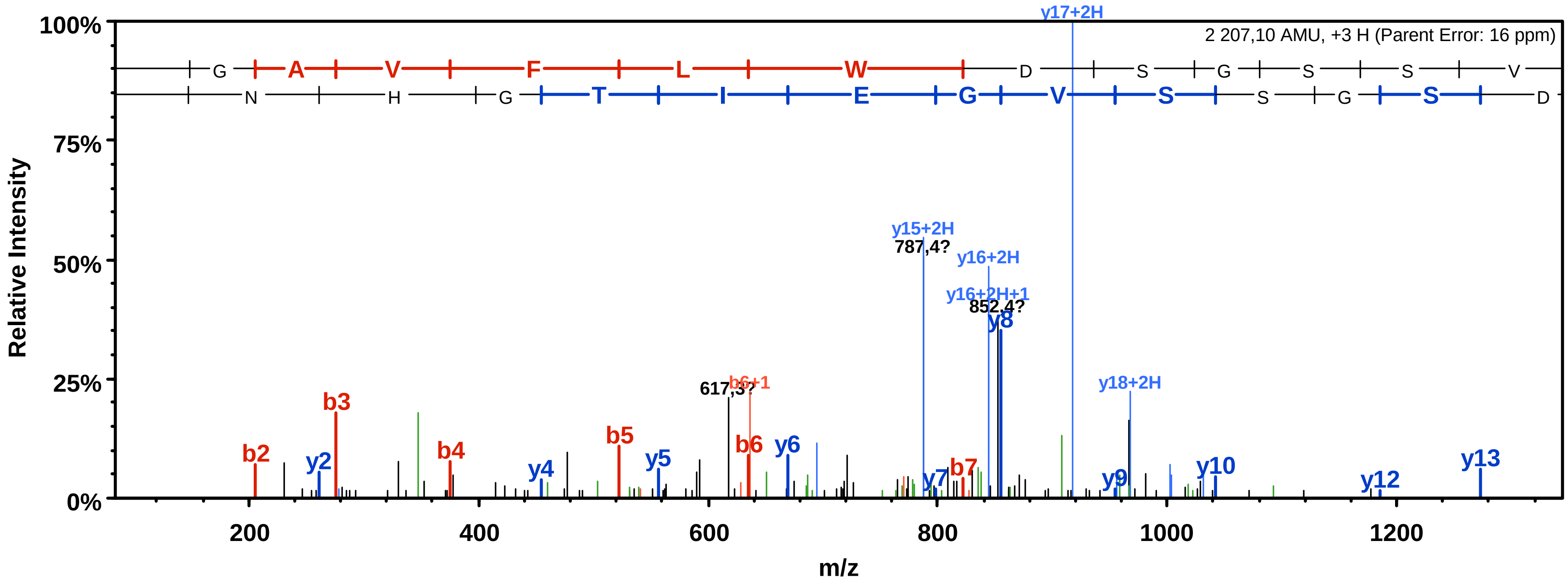

Biological sample name	MS/MS sample name	Protein name	Peptide sequence	Mascot Ion score
**Chromofungin_14**	F011546 (F013446)	WD repeat-containing protein 1 - Homo sapiens (Human)	FGAVFLWDSGSSVGEITGHNK	60,6

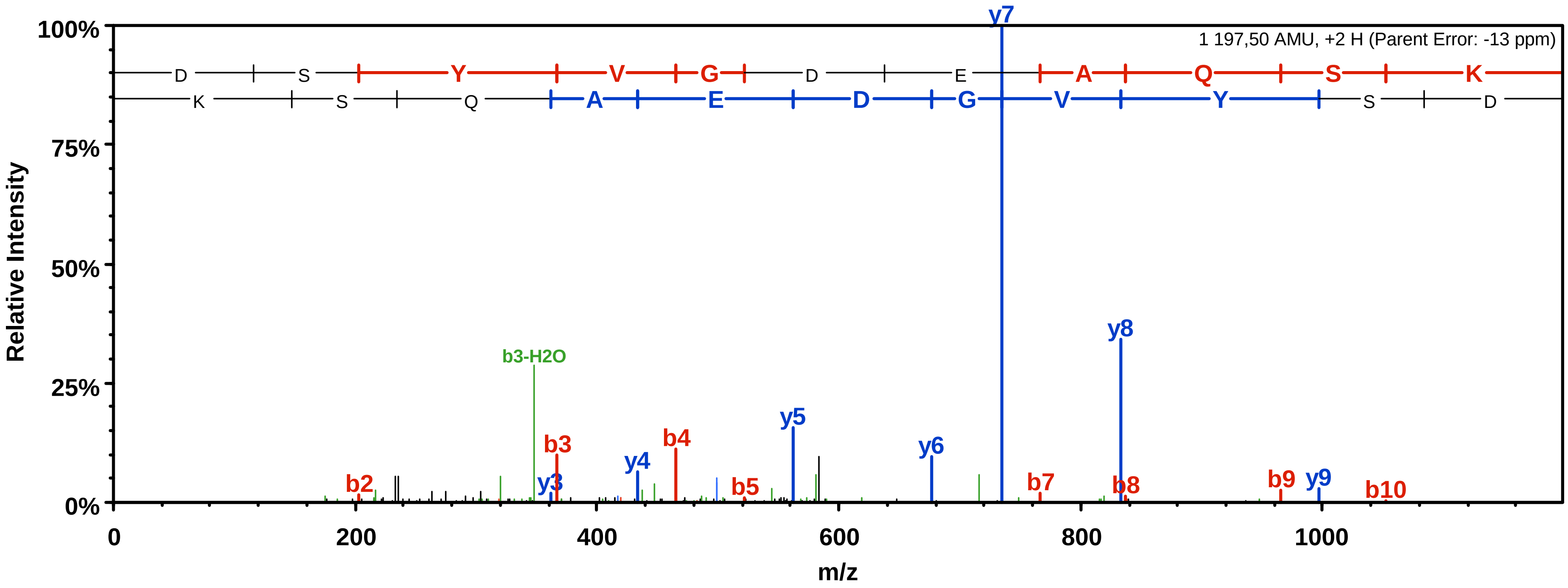

Biological sample name	MS/MS sample name	Protein name	Peptide sequence	Mascot Ion score
**Castestatin_6**	F011558 (F013468)	Actin, cytoplasmic 1 - Homo sapiens (Human)	DSYVGDEAQSK	63,3

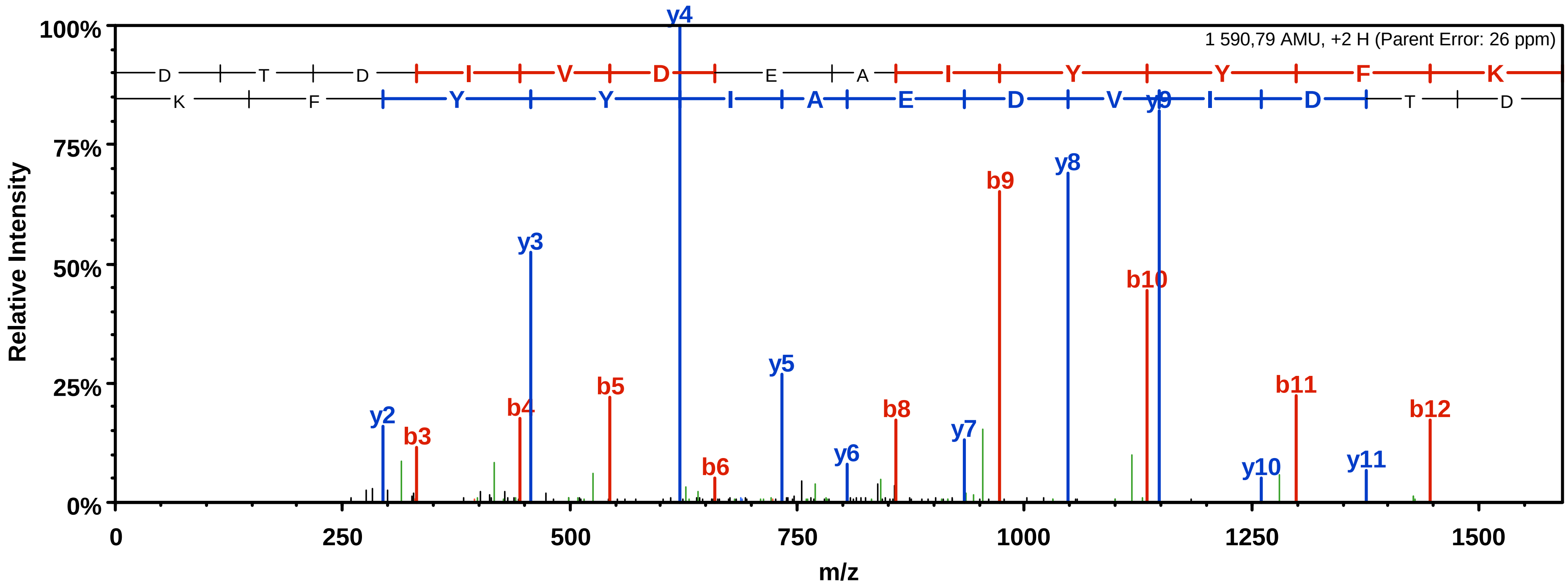

Biological sample name	MS/MS sample name	Protein name	Peptide sequence	Mascot Ion score
**Castestatin_14**	F011566 (F013485)	Actin-related protein 2/3 complex subunit 3 - Homo sapiens (Human)	DTDIVDEAIYYFK	97,4

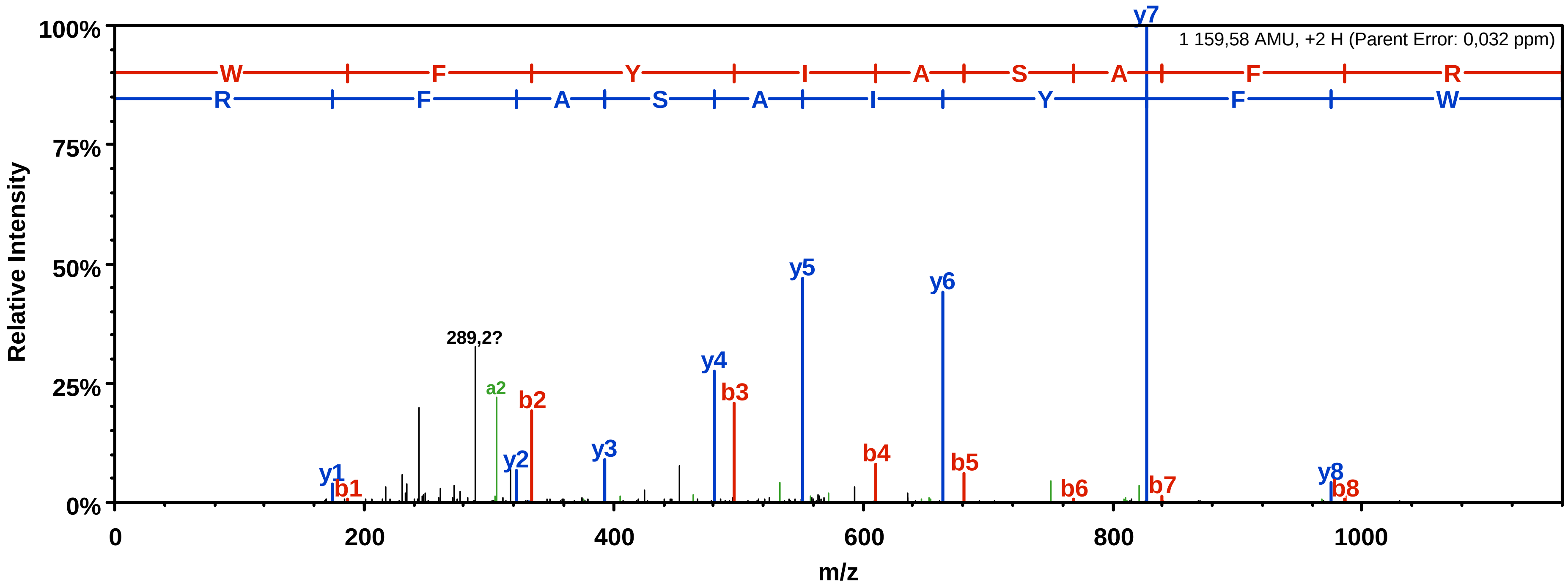

Biological sample name	MS/MS sample name	Protein name	Peptide sequence	Mascot Ion score
**Castestatin_11**	F011563 (F013479)	Alpha-1-acid glycoprotein 1 precursor - Homo sapiens (Human)	WFYIASAFR	54,1

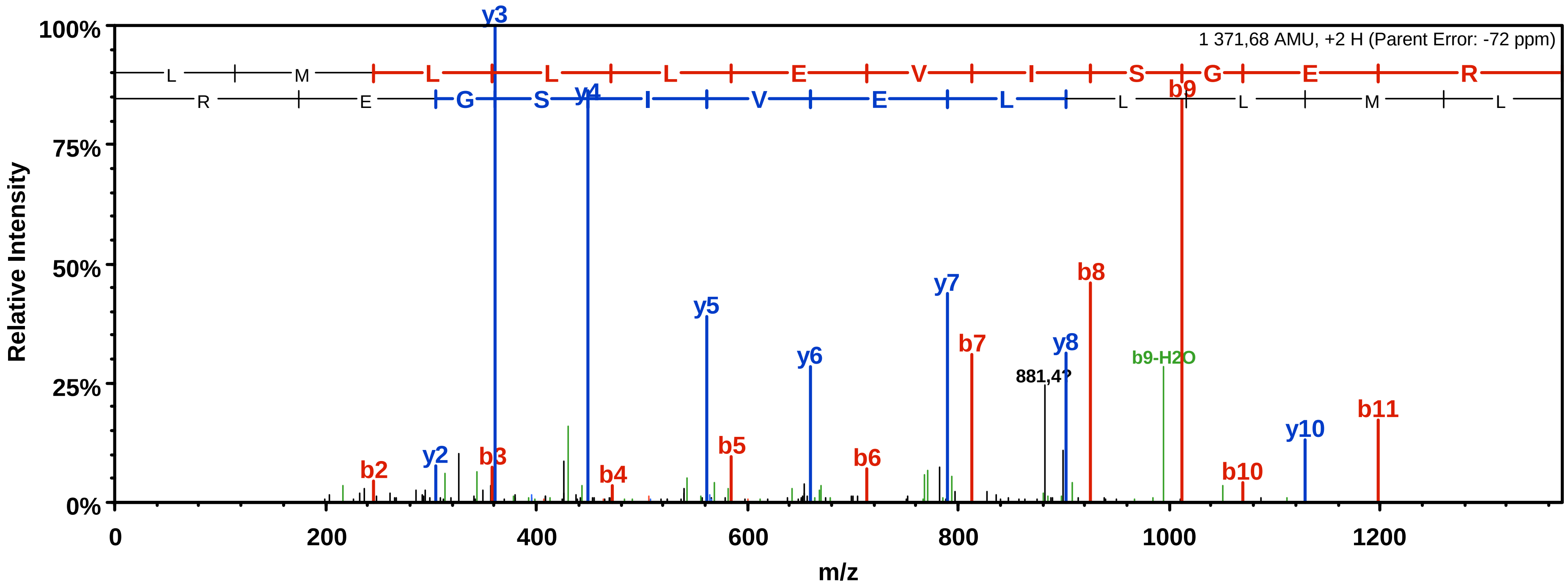

Biological sample name	MS/MS sample name	Protein name	Peptide sequence	Mascot Ion score
**Castestatin_16**	F011568 (F013490)	Alpha-actinin-1 - Homo sapiens (Human)	LMLLLEVISGER	72,9

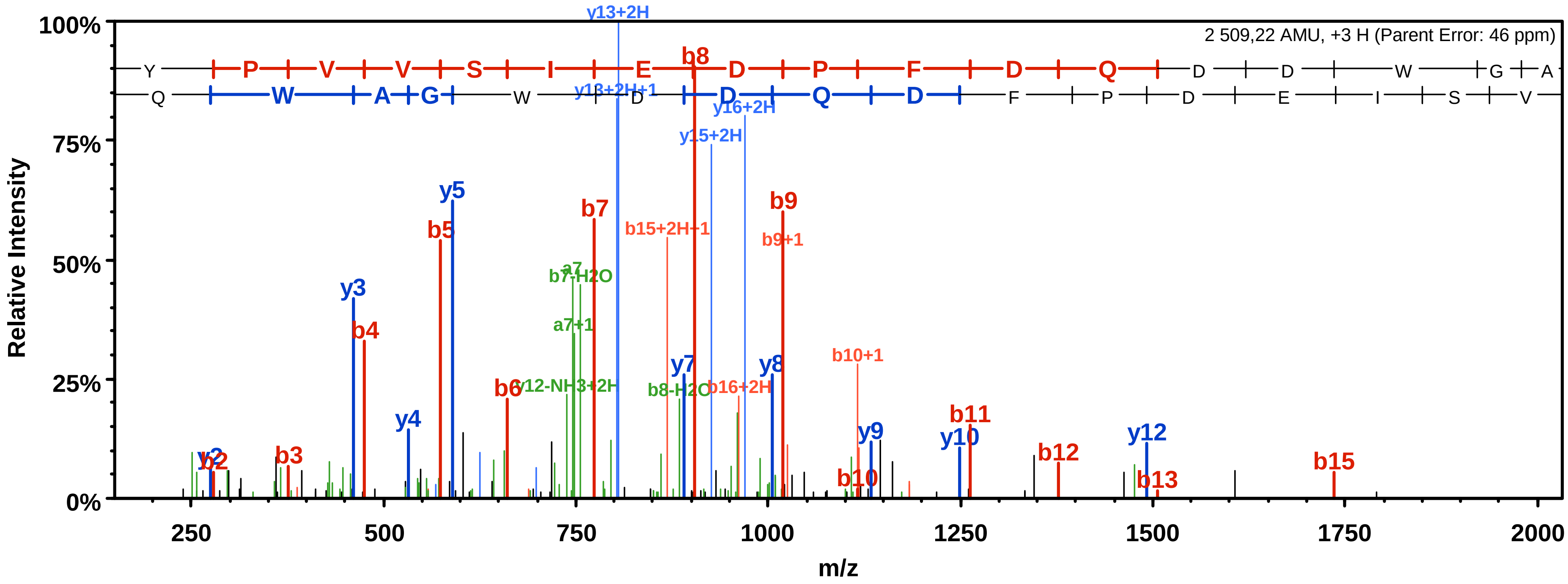

Biological sample name	MS/MS sample name	Protein name	Peptide sequence	Mascot Ion score
**Castestatin_14**	F011566 (F013485)	Alpha-enolase - Homo sapiens (Human)	DYPVVSIEDPFDQDDWGAWQK	84,6

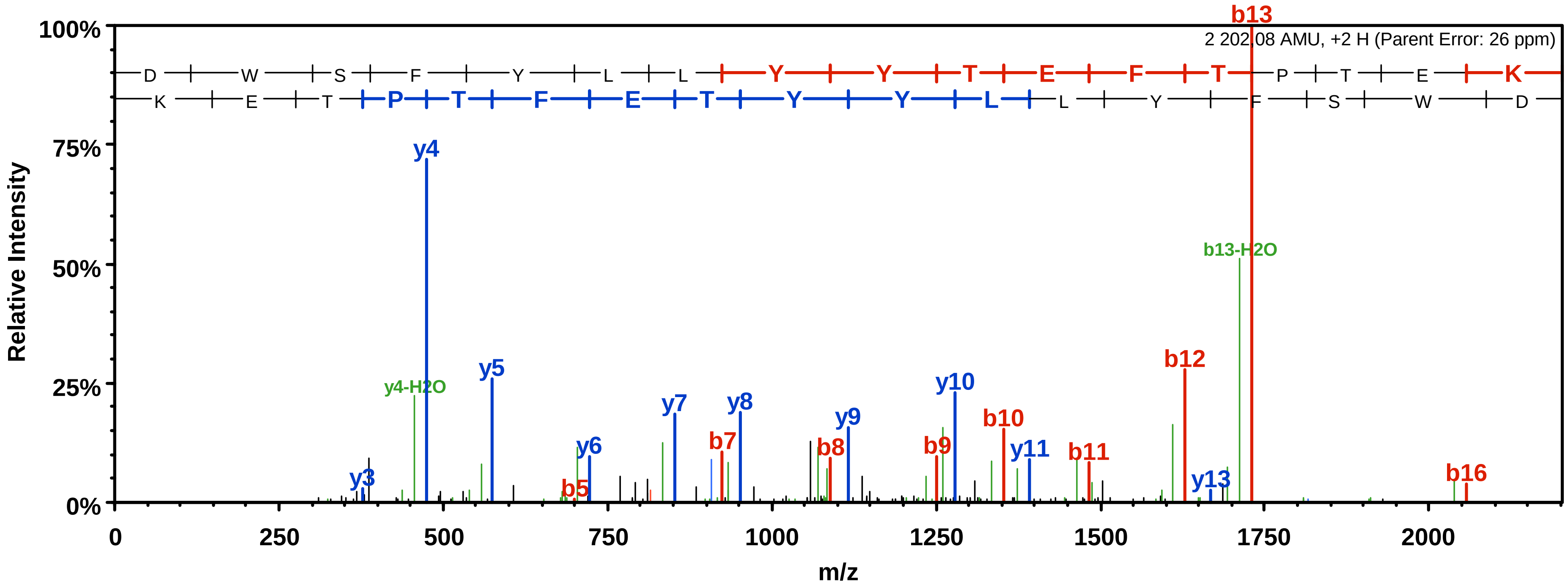

Biological sample name	MS/MS sample name	Protein name	Peptide sequence	Mascot Ion score
**Castestatin_11**	F011563 (F013479)	Beta-2-microglobulin precursor [Contains: Beta-2-microglobulin form pI 5.3] - Homo sapiens (Human)	DWSFYLLYYTEFTPTEK	62,4

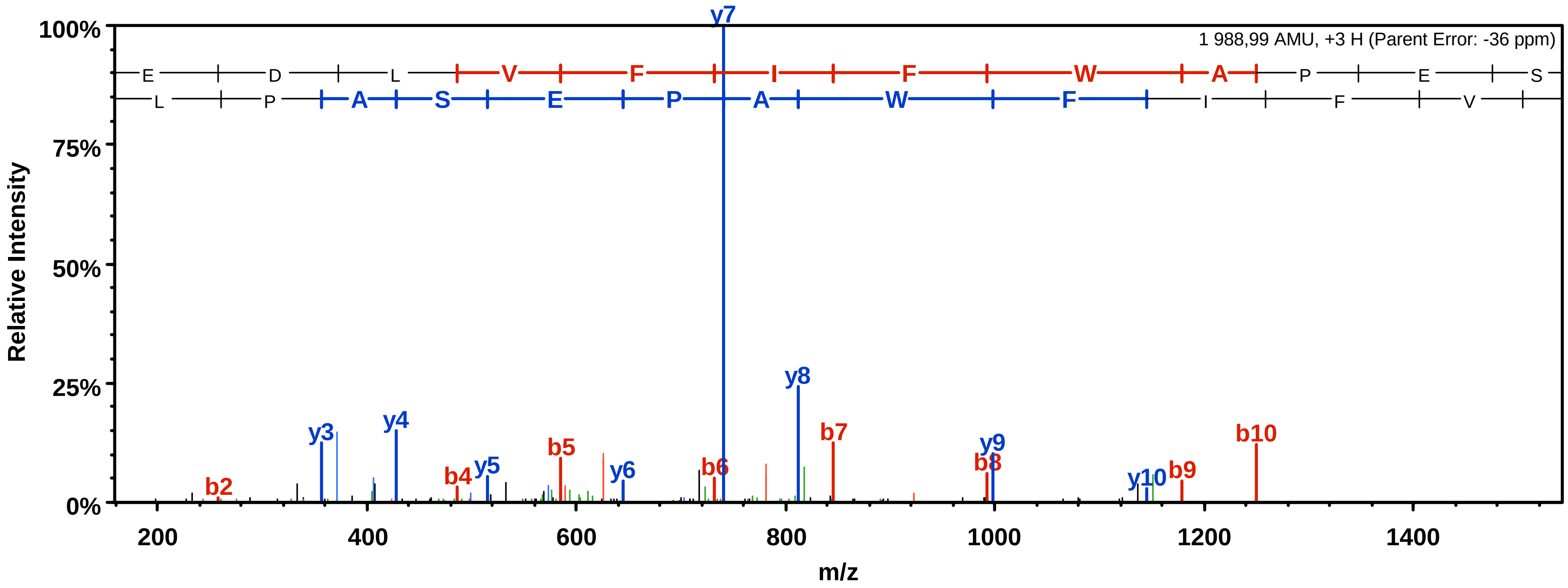

Biological sample name	MS/MS sample name	Protein name	Peptide sequence	Mascot Ion score
**Castestatin_12**	F011564 (F013481)	Cofilin-1 - Homo sapiens (Human)	KEDLVFIFWAPESAPLK	56,9

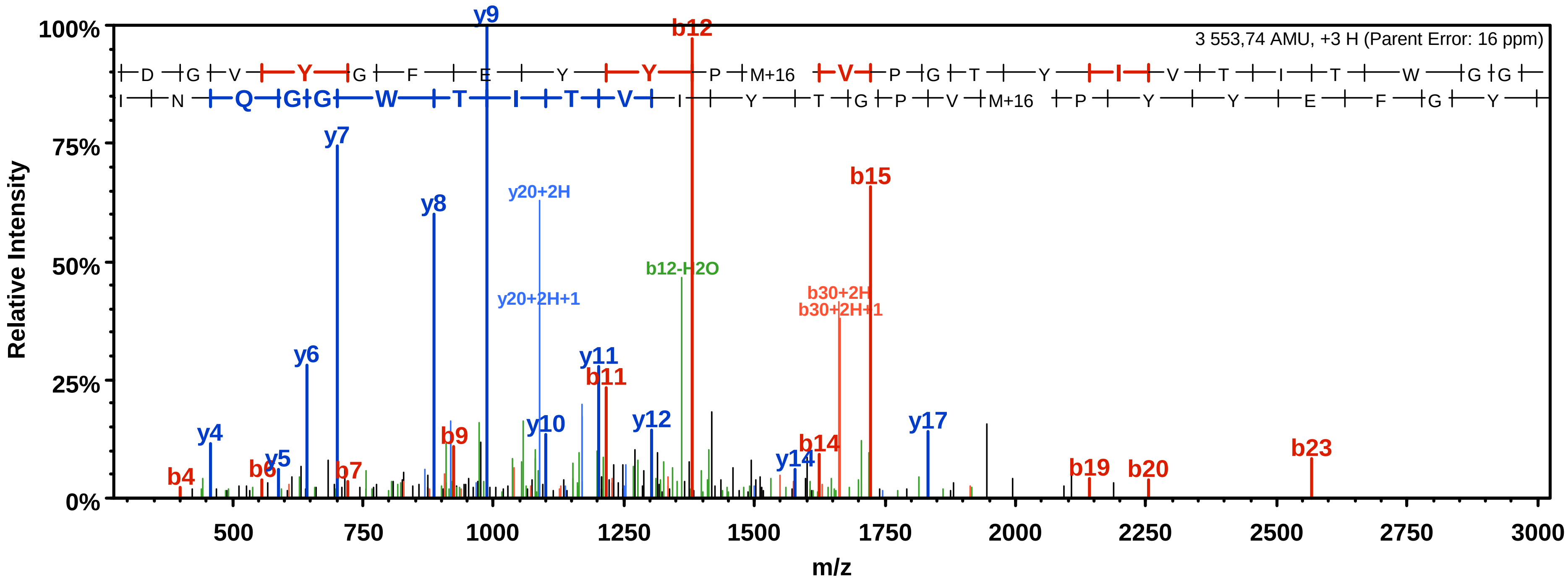

Biological sample name	MS/MS sample name	Protein name	Peptide sequence	Mascot Ion score
**Castestatin_11**	F011563 (F013479)	Filamin-A - Homo sapiens (Human)	DLGDGVYGFEYYPMVPGTYIVTITW GGQNIGR	63,9

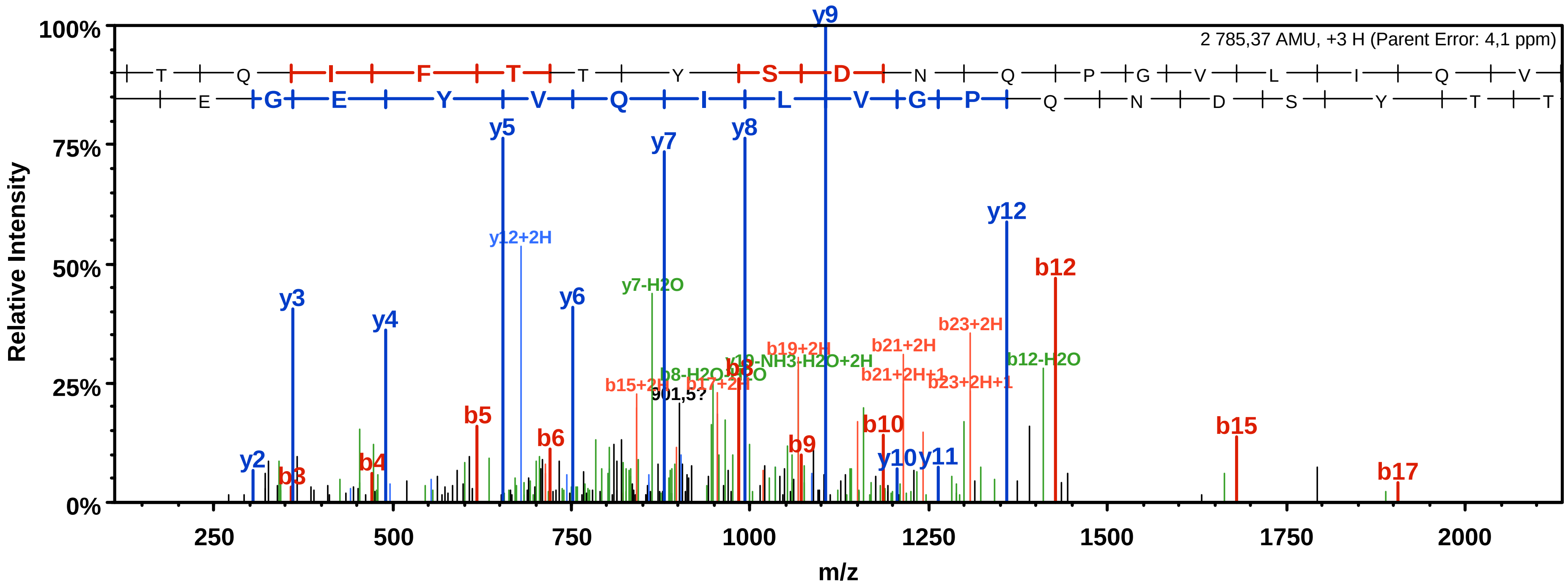

Biological sample name	MS/MS sample name	Protein name	Peptide sequence	Mascot Ion score
**Castestatin_13**	F011565 (F013483)	Heat shock 70 kDa protein 1 - Homo sapiens (Human)	QTQIFTTYSDNQPGVLIQVYEGER	69,3

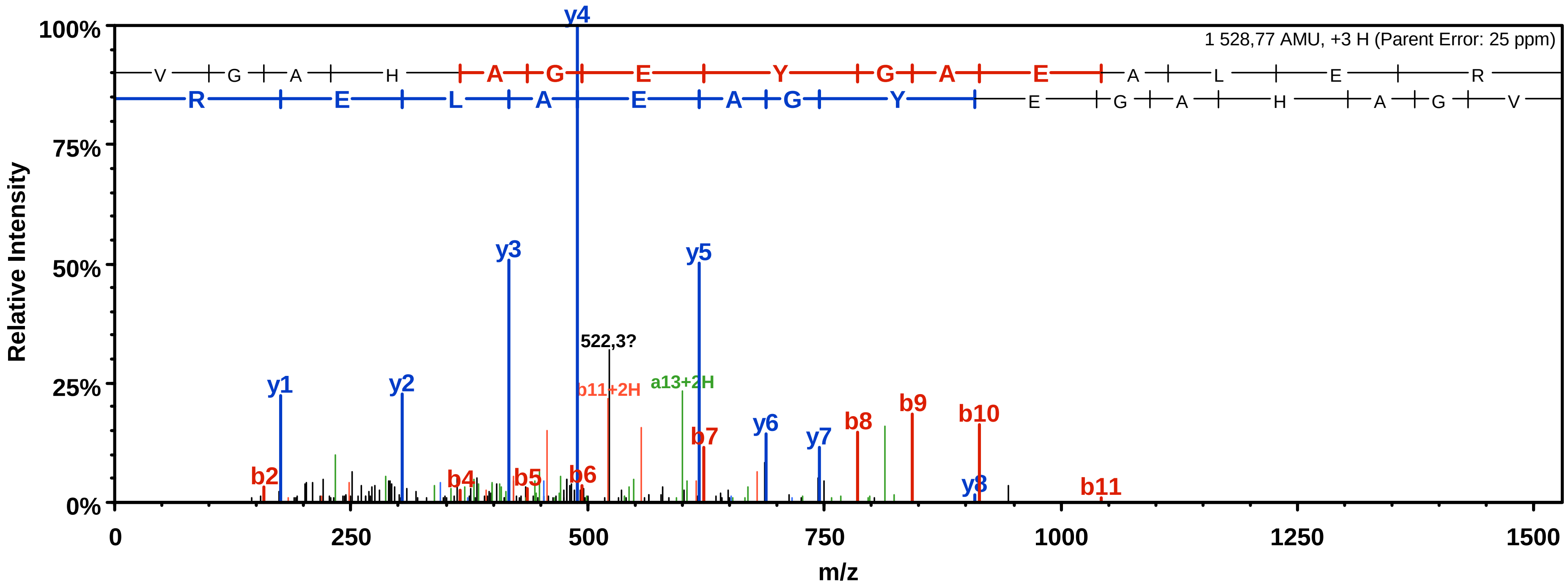

Biological sample name	MS/MS sample name	Protein name	Peptide sequence	Mascot Ion score
**Castestatin_8**	F011560 (F013472)	Hemoglobin subunit alpha - Homo sapiens (Human)	VGAHAGEYGAEALER	56,6

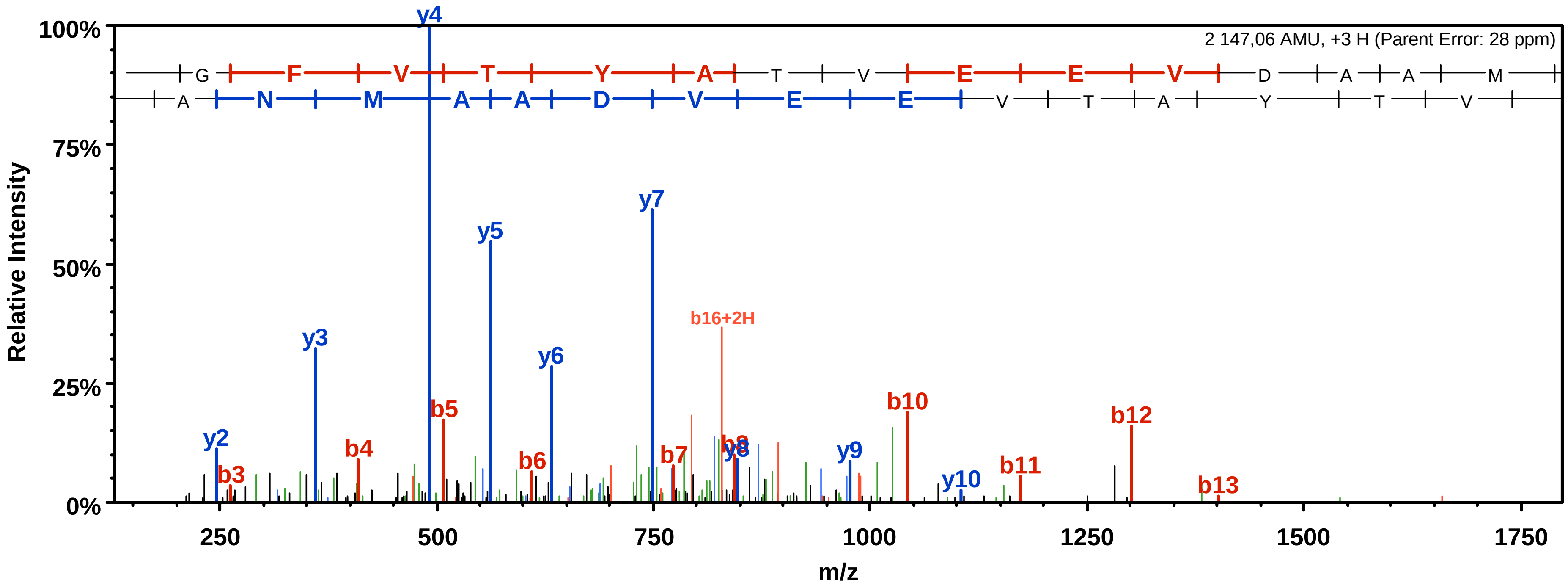

Biological sample name	MS/MS sample name	Protein name	Peptide sequence	Mascot Ion score
**Castestatin_11**	F011563 (F013479)	Heterogeneous nuclear ribonucleoprotein A1 - Homo sapiens (Human)	GFGFVTYATVEEVDAAMNAR	64,4

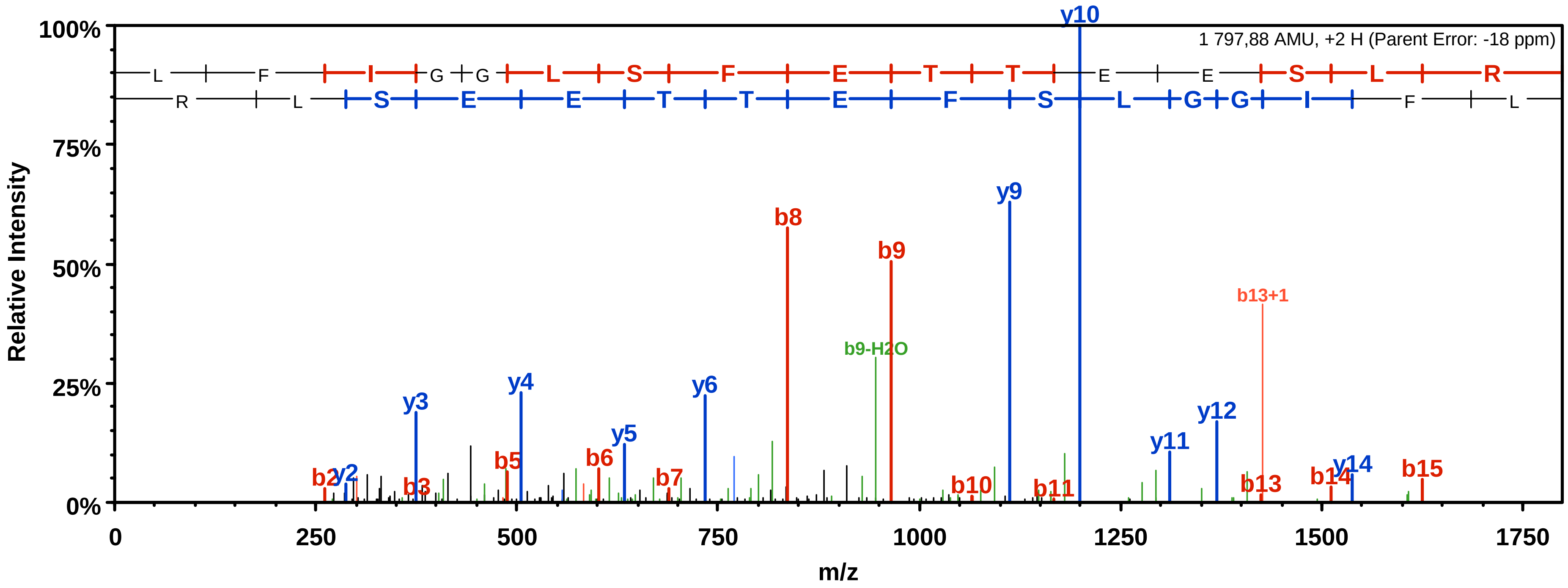

Biological sample name	MS/MS sample name	Protein name	Peptide sequence	Mascot Ion score
**Castestatin_12**	F011564 (F013481)	Heterogeneous nuclear ribonucleoproteins A2/B1 - Homo sapiens (Human)	LFIGGLSFETTEESLR	113

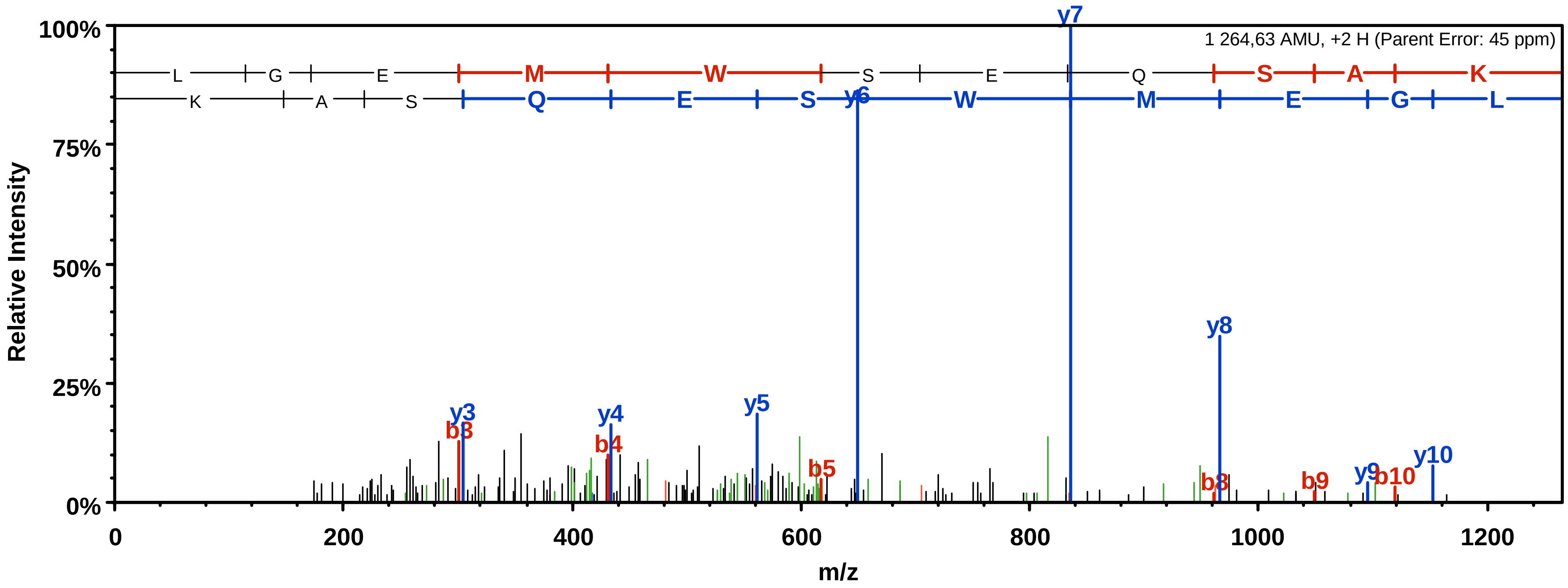

Biological sample name	MS/MS sample name	Protein name	Peptide sequence	Mascot Ion score
**Castestatin_10**	F011562 (F013477)	High mobility group protein B2 - Homo sapiens (Human)	LGEMWSEQSAK	57,1

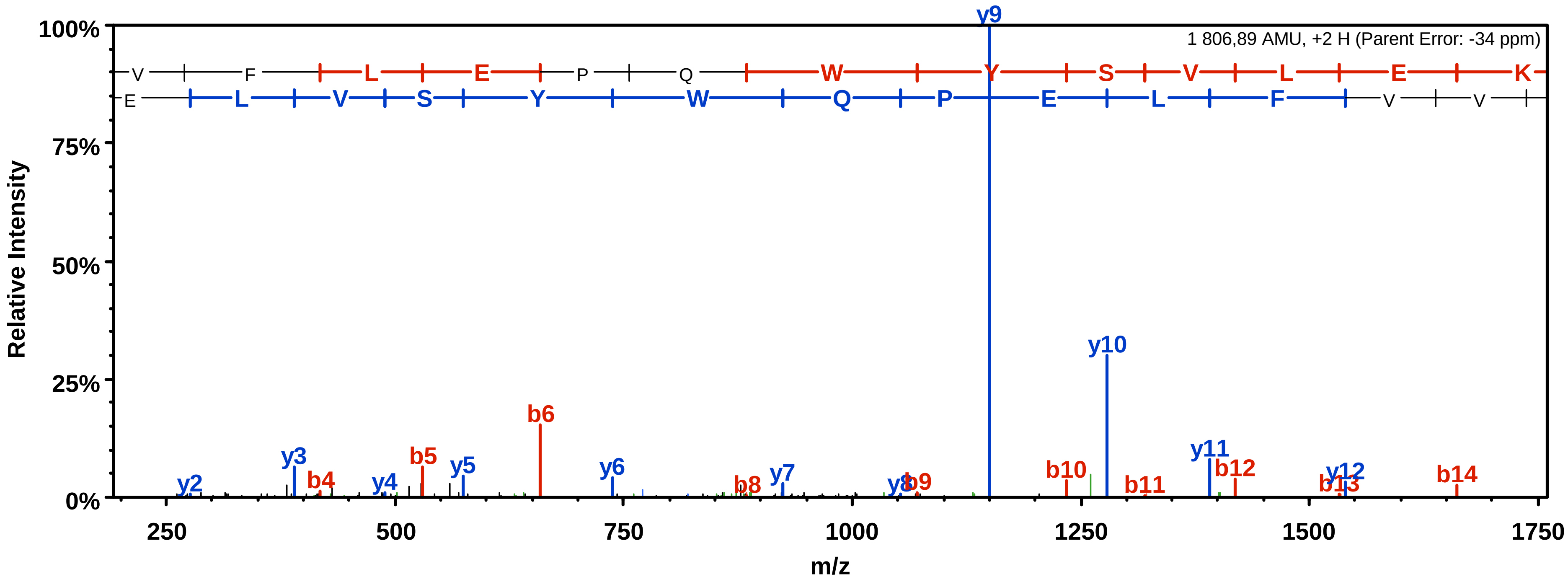

Biological sample name	MS/MS sample name	Protein name	Peptide sequence	Mascot Ion score
**Castestatin_11**	F011563 (F013479)	Low affinity immunoglobulin gamma Fc region receptor III-B precursor - Homo sapiens (Human)	AVVFLEPQWYSVLEK	60,5

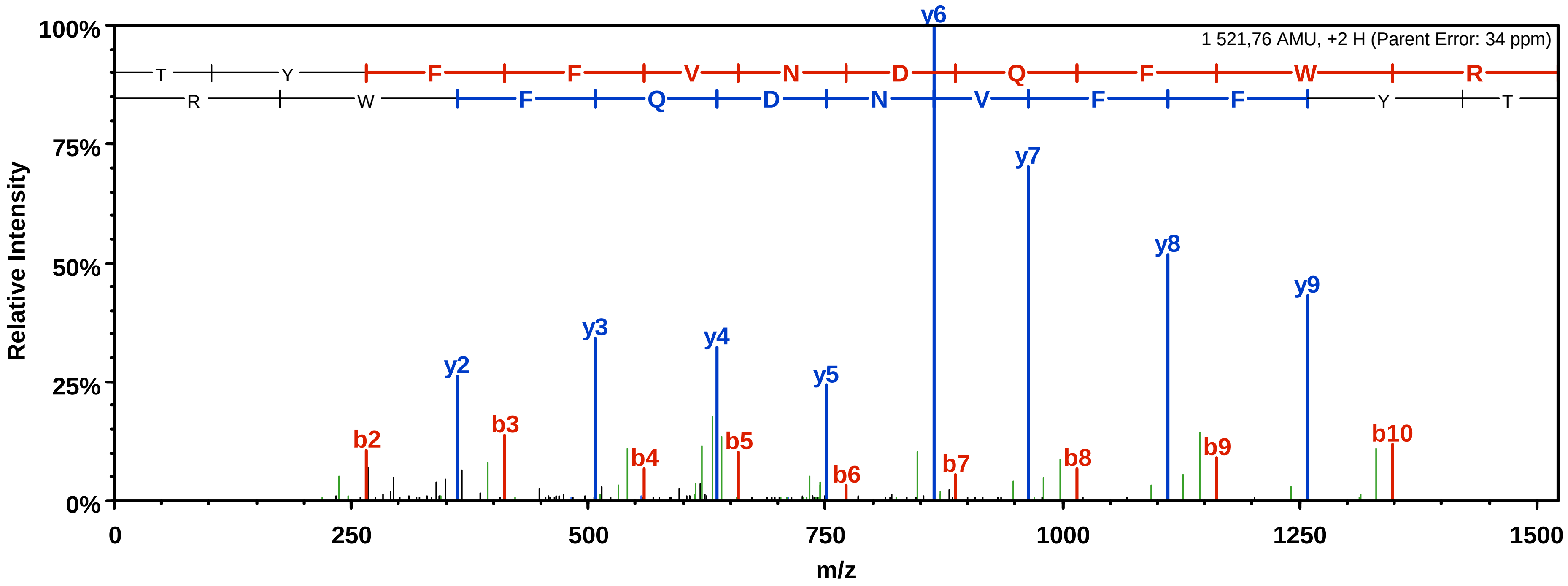

Biological sample name	MS/MS sample name	Protein name	Peptide sequence	Mascot Ion score
**Castestatin_15**	F011567 (F013488)	Neutrophil collagenase precursor - Homo sapiens (Human)	TYFFVNDQFWR	84,7

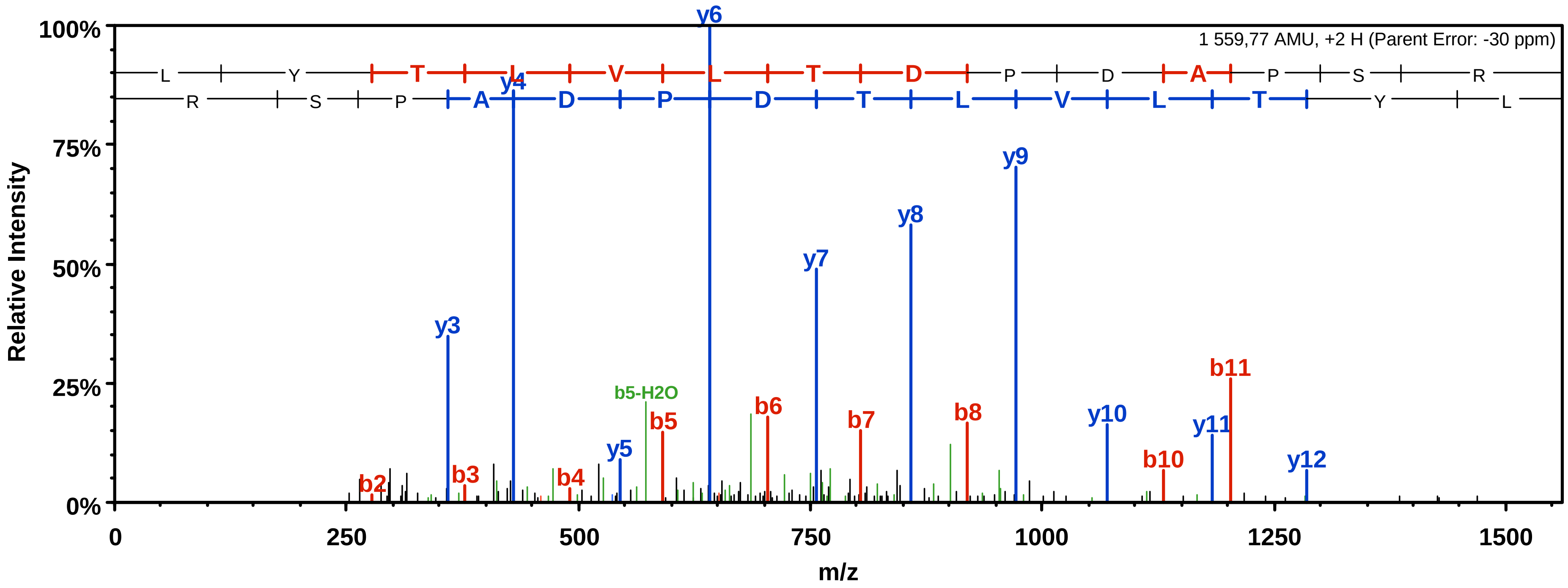

Biological sample name	MS/MS sample name	Protein name	Peptide sequence	Mascot Ion score
**Castestatin_11**	F011563 (F013479)	Phosphatidylethanolamine-binding protein 1 - Homo sapiens (Human)	LYTLVLTDPDAPSR	83,3

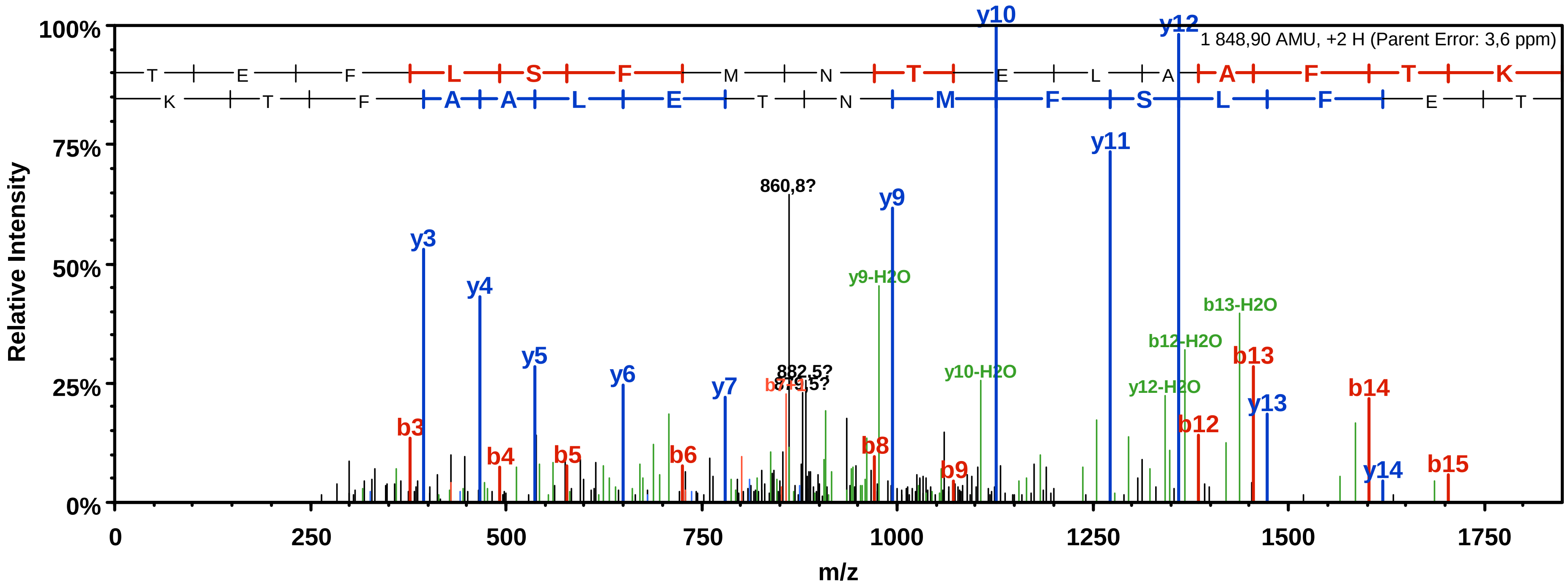

Biological sample name	MS/MS sample name	Protein name	Peptide sequence	Mascot Ion score
**Castestatin_14**	F011566 (F013485)	Protein S100-A11 - Homo sapiens (Human)	TEFLSFMNTELAAFTK	66,8

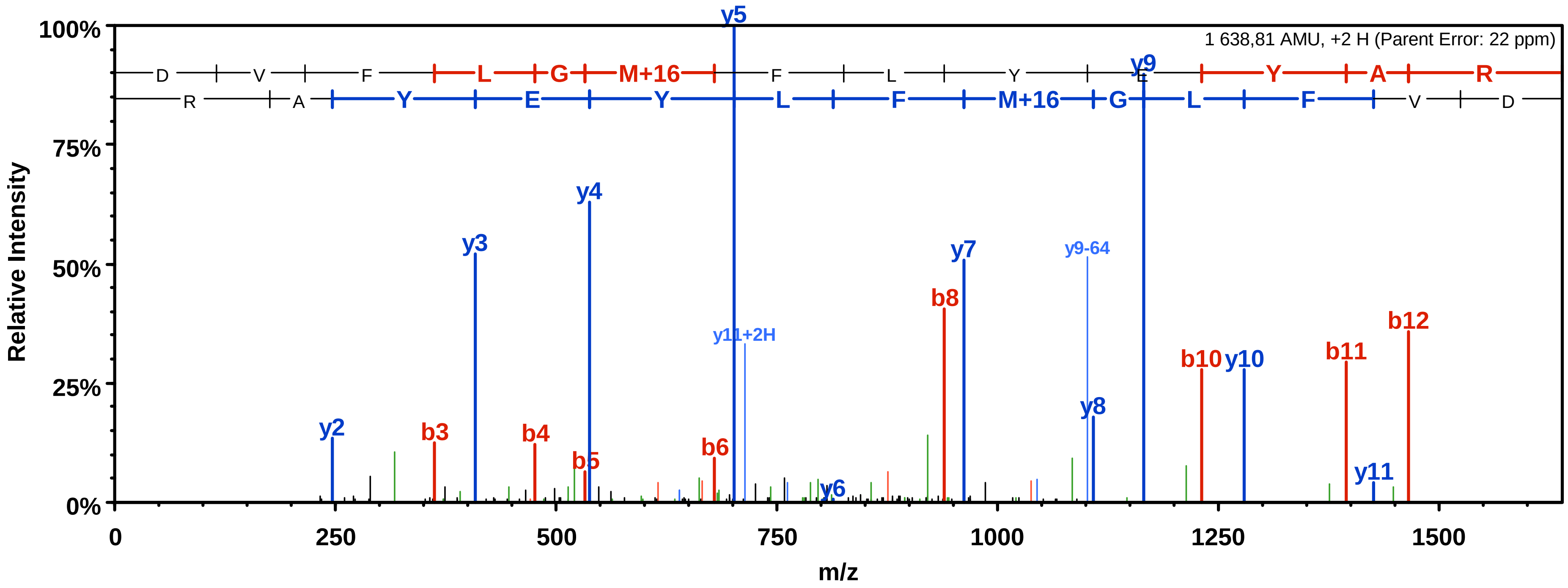

Biological sample name	MS/MS sample name	Protein name	Peptide sequence	Mascot Ion score
**Castestatin_13**	F011565 (F013483)	Serum albumin precursor - Homo sapiens (Human)	DVFLGMFLYEYAR	77,1

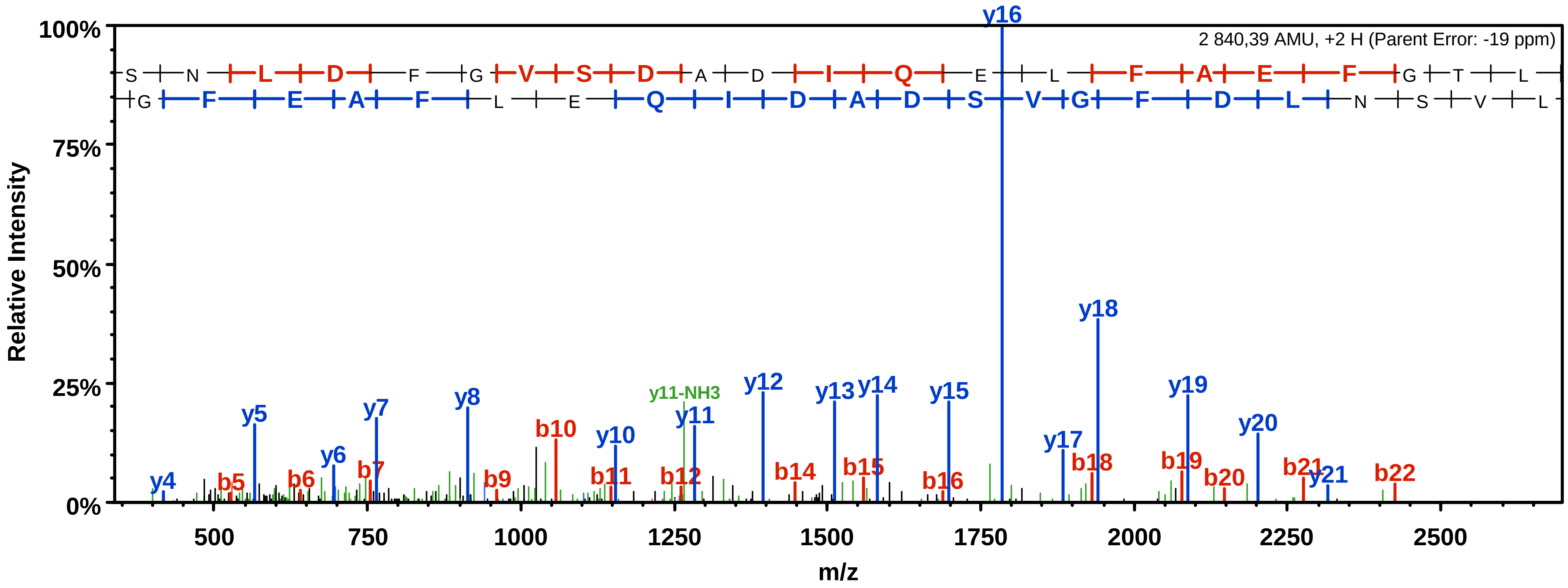

Biological sample name	MS/MS sample name	Protein name	Peptide sequence	Mascot Ion score
**Castestatin_12**	F011564 (F013481)	THO complex subunit 4 - Homo sapiens (Human)	LLVSNLDFGVSDADIQELFAEFGTLK	137

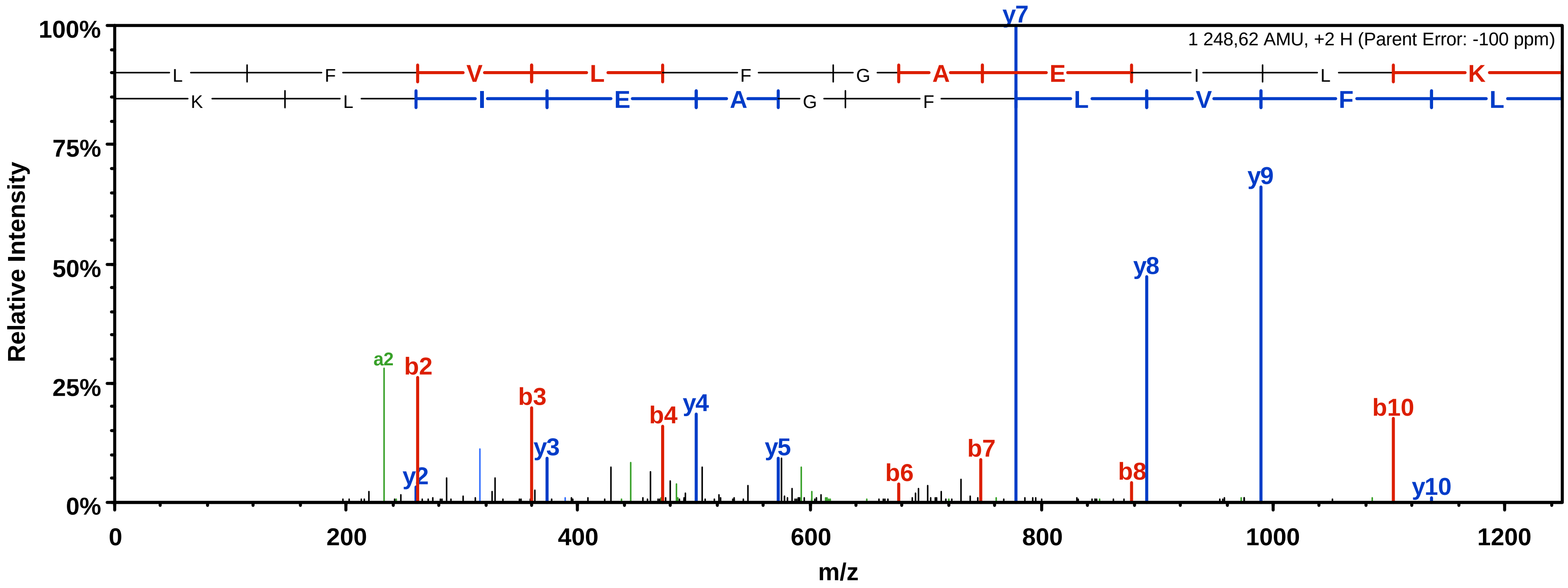

Biological sample name	MS/MS sample name	Protein name	Peptide sequence	Mascot Ion score
**Castestatin_16**	F011568 (F013490)	Transaldolase - Homo sapiens (Human)	LFVLFGAEILK	59,1

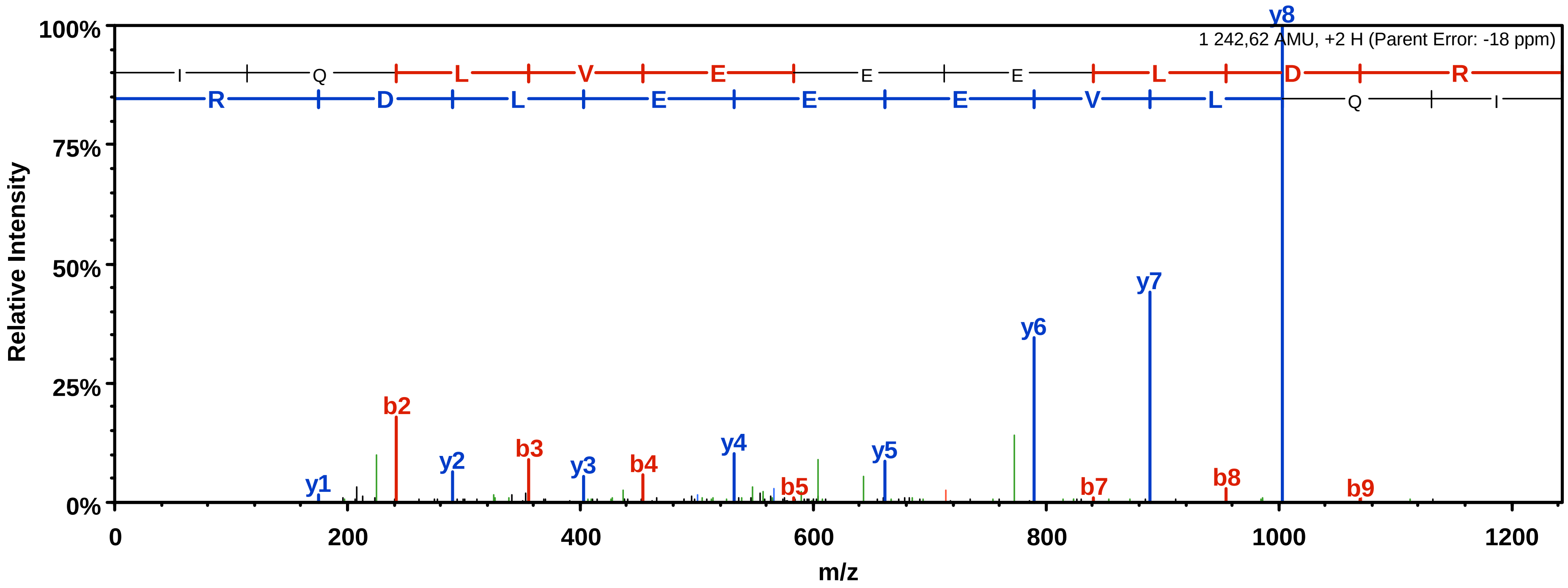

Biological sample name	MS/MS sample name	Protein name	Peptide sequence	Mascot Ion score
**Castestatin_13**	F011565 (F013483)	Tropomyosin alpha-3 chain - Homo sapiens (Human)	IQLVEEELDR	60,2

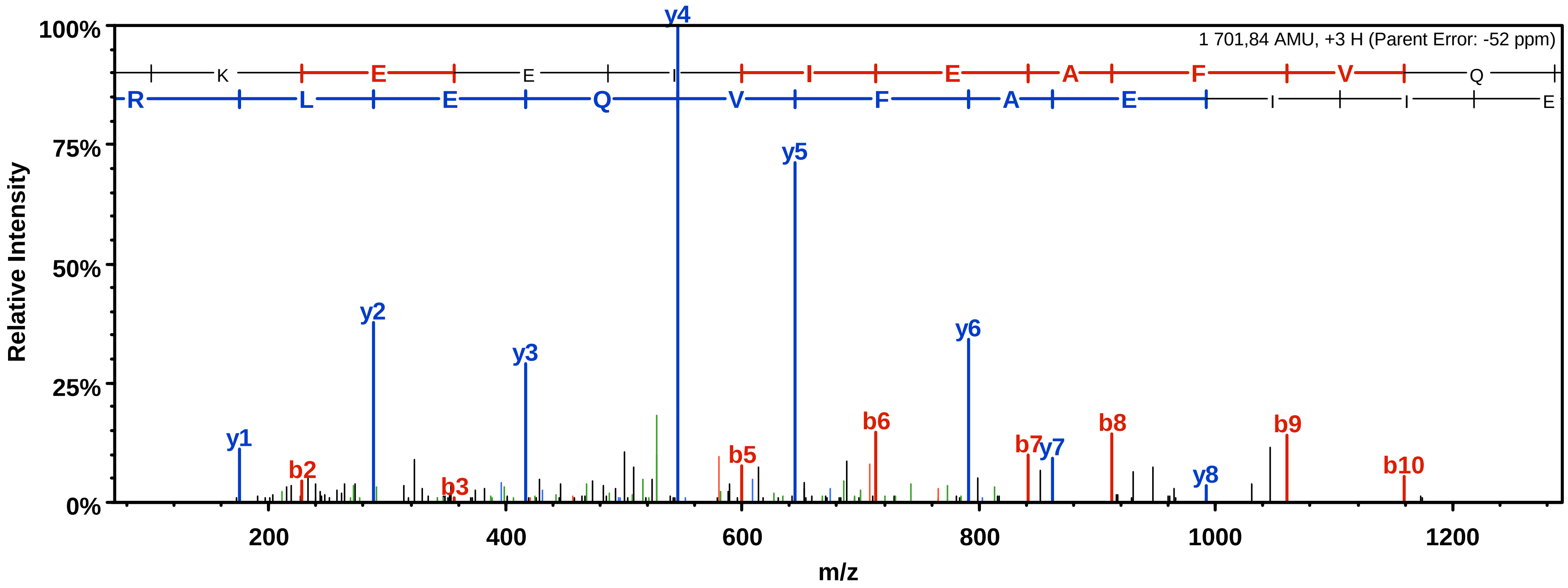

Biological sample name	MS/MS sample name	Protein name	Peptide sequence	Mascot Ion score
**Castestatin_14**	F011566 (F013485)	Vasodilator-stimulated phosphoprotein - Homo sapiens (Human)	VKEEIIEAFVQELR	56,3

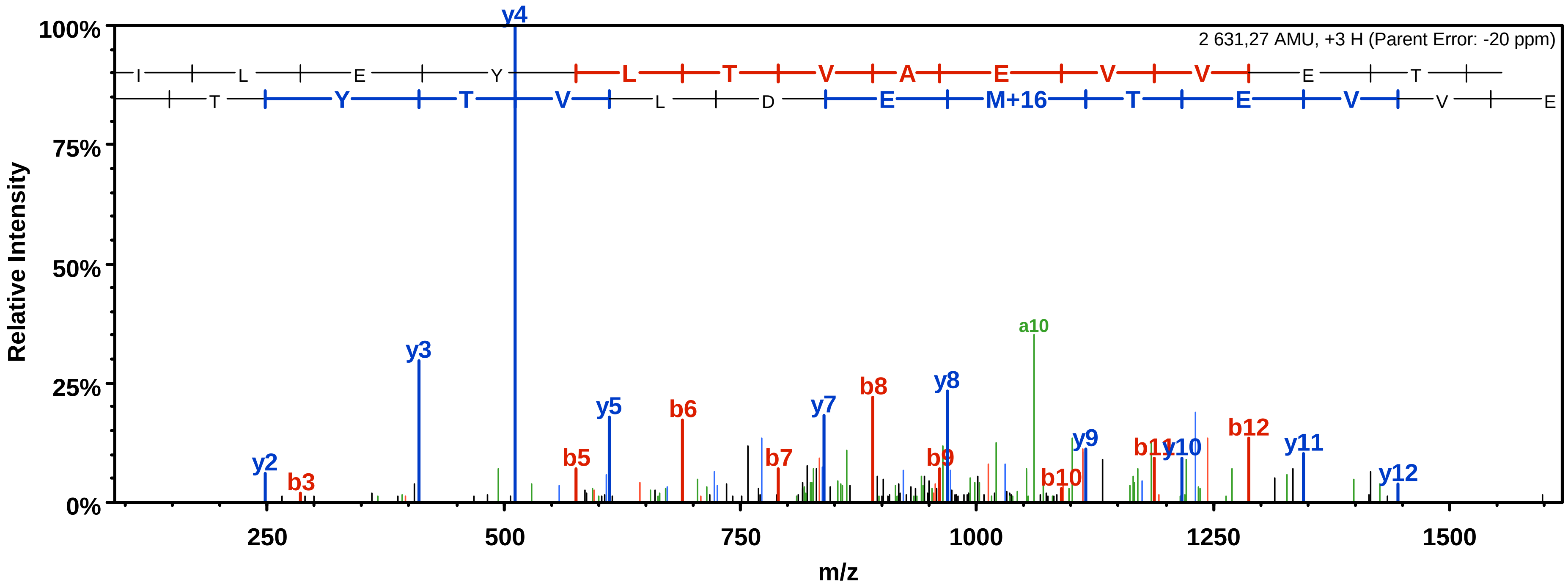

Biological sample name	MS/MS sample name	Protein name	Peptide sequence	Mascot Ion score
**Castestatin_16**	F011568 (F013490)	Vinculin - Homo sapiens (Human)	GILEYLTVAEVVETMEDLVTYTK	61,9
